# Marine natural products from sponges (Porifera) of the order Dictyoceratida (2013 to 2019); a promising source for drug discovery†

**DOI:** 10.1039/d0ra04408c

**Published:** 2020-09-21

**Authors:** Enas Reda Abdelaleem, Mamdouh Nabil Samy, Samar Yehia Desoukey, Miaomiao Liu, Ronald J. Quinn, Usama Ramadan Abdelmohsen

**Affiliations:** Department of Pharmacognosy, Faculty of Pharmacy, Minia University 61519 Minia Egypt usama.ramadan@mu.edu.eg; Griffith Institute for Drug Discovery, Griffith University Brisbane Queensland 4111 Australia; Department of Pharmacognosy, Faculty of Pharmacy, Deraya University Universities Zone, 61111 New Minia City Minia Egypt

## Abstract

Marine organisms have been considered an interesting target for the discovery of different classes of secondary natural products with wide-ranging biological activities. Sponges which belong to the order Dictyoceratida are distinctly classified into 5 families: Dysideidae, Irciniidae, Spongiidae, Thorectidae, and Verticilliitidae. In this review, compounds isolated from Dictyoceratida sponges were discussed with their biological potential within the period 2013 to December 2019. Moreover, analysis of the physicochemical properties of these marine natural products was investigated and the results showed that 78% of the compounds have oral bioavailability potential. This review highlights sponges of the order Dictyoceratida as exciting source for discovery of new drug leads.

## Abstract 2

Marine organisms have been considered an interesting target for the discovery of different classes of secondary natural products with wide-ranging biological activities.

## Graphical abstract

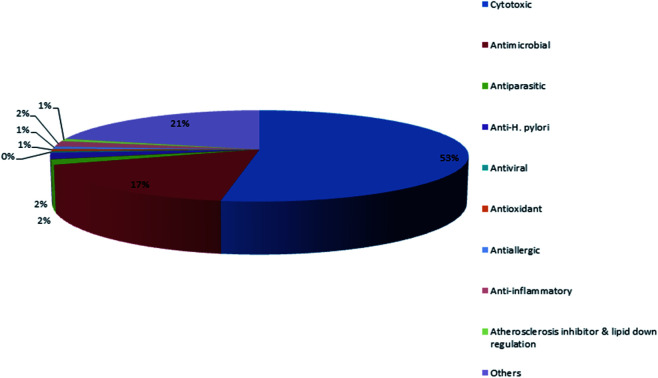

## 1. Introduction

Marine life forms are widely variable, including sponges, corals, ascidians, gorgonians, sea pens, algae, fungi and marine associated micro-organisms which are considered important sources for the discovery of structurally diverse and bioactive secondary metabolites.^1^ According to the World Porifera Database,^2^ the order Dictyoceratida has been divided into 5 distinct families: Dysideidae, Irciniidae, Spongiidae, Thorectidae, and Verticilliitidae. The family Verticilliitidae has been reclassified recently into this order and contains two genera. Each family possesses its distinctive characteristics such as the fine collagenous filaments in the irciniids, the homogeneous skeletal fibers of spongiids, the eurypylous choanocyte chambers in dysideids and the opposite of these characteristics in the thorectids (as examples the diplodal choanocyte chambers, pithed and laminated fibers and absence of fine filaments).^3^ There are two reasons which attract us to discuss this order of sponges. The first reason is the abundant geographical distribution where they were reported from the waters of 31 countries. The major contributors were Korea, Japan, Australia, China, Papua New Guinea and Indonesia. The second reason is the chemical richness and diversity of their metabolites. The order Dictyoceratida (Phylum Porifera, Class Demospongiae, Order Dictyoceratida) has contributed over 20% of new secondary metabolites which were previously derived from all sponges, making it the highest producer among all the sponge orders. In contrast, the orders Haplosclerida, Poecilosclerida, Halichondrida, and Astrophorida contributed with 14.2%, 14%, 10.7% and 9.2%, respectively. The order Lithistida only contributed with 5.5%.^4^ Mehbub *et al.* published a valuable review on the secondary metabolites isolated from the order Dictyoceratida and their biological activities during the period from 2001–2012.^4^ This provoked us to establish our review as a part 2 during the period from 2013–2019. The compounds were reported to exhibit different biological activities including cytotoxic, antimicrobial, antiparasitic, anti-*H. pylori*, antiviral, antioxidant, antiallergic, anti-inflammatory, inhibition of atherosclerosis and other activities.

**Fig. 1:**
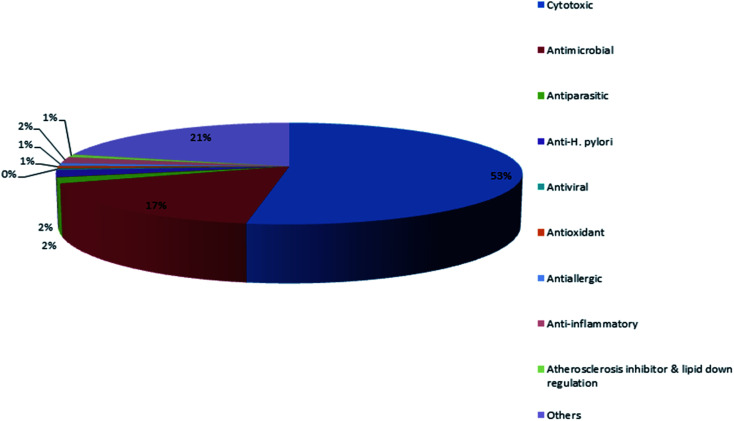
Different biological activities of metabolites isolated from Dictyoceratida sponges.

These results agreed with the previous reported review where the major detected two bioactivities exhibited by metabolites were the cytotoxic activity followed by the antimicrobial activity Fig. 1.

In this review, we report all published data regarding secondary metabolites isolated from Dictyoceratida sponges and their biological activities within the period 2013 to Dec 2019 (Table S1†). In addition, we highlighted the most bioactive compounds with activity less than or equal to 5 μM or 5 μg mL^−1^.

## 2. Potent bioactive compounds isolated from Dictyoceratida sponges within 2013–2019

### 2.1 Potent bioactive compounds isolated from family Thorectidae

Within 2013–2019, 195 new compounds and 189 known compounds were isolated from different genera. Three new alkaloids hyrtioerectines D, E & F (1–3) were isolated from a *Hyrtios* sp. and showed antimicrobial activity. Also, hyrtioerectines D (1) and F (3) were more active than hyrtioerectine E (2) with 45% and 42% of inhibition in a DPPH free radical scavenging activity test.^5^ Another two new alkaloids (4, 5) were isolated from the same sponge. Hyrtimomine A (4) showed *in vitro* cytotoxic activity against human epidermoid carcinoma KB cells and muline leukemia L1210 cells with IC_50_ value of 3.1 μg mL^−1^ and 4.2 μg mL^−1^, respectively.^6^ Hyrtimomines A (4) and B (5) showed antimicrobial activities against *Candida albicans* (IC_50_: 1.0 μg mL^−1^, each) and *C. neoformans* (IC_50_: 2.0 and 4.0 μg mL^−1^, respectively). Also, hyrtimomine A (4) exhibited an antifungal activity against *Aspergillus niger* (IC_50_: 4.0 μg mL^−1^).^7^

Hyrtimomine D (6), a bisindole alkaloid from *Hyrtios* sp. was reported to exhibit an inhibitory activity against *C. albicans* and *Cryptococcus neoformans* (IC_50_: 4 μg mL^−1^) and showed inhibitory activity against *Staphylococcus aureus* with MIC value of 4 μg mL^−1^.^6^ Hyrtimomine I (7) showed antifungal effect against *C. neoformans* (IC_50_: 4.0 μg mL^−1^).^7^

**Fig. 2:**
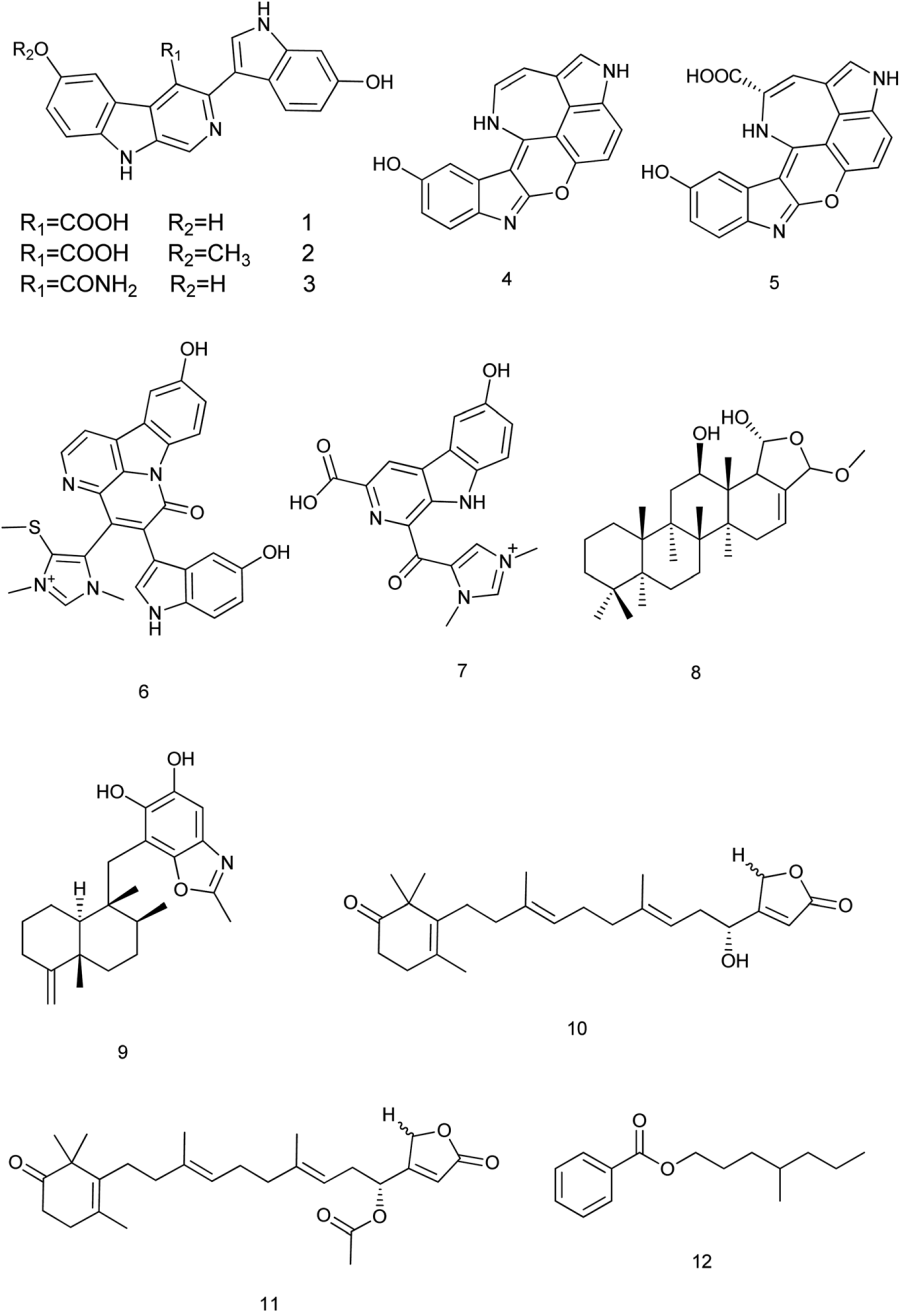
Structures of compounds (1–12).

A new scalarane sesterterpene, the hemiacetal of 12-deacetyl-*cis*-24α,25α-dimethoxyscalarin (8) from *Hyrtios* sp. showed inhibition of TDP-43 binding to its DNA target strand and stimulated its cytoplasmic translocation and its tendency to aggregate. This could be considered as a chemical exploration in the study of the TDP-43 aggregation state and its cellular localization.^8^ Nakijinol G (9), a new meroterpenoid from *Hyrtios* sp. was evaluated for its protein tyrosine phosphatase (PTP1B) inhibitory and cytotoxic activities. Results showed a significant PTP1B inhibition with an IC_50_ value of 4.8 μM, where no cytotoxicity against four human cancer cell lines was detected.^9^ From the sponge *H. communis*, thorectidaeolide A (10) and 4-acetoxythorectidaeolide A (11), two new sesterterpenes were isolated and reported to be highly potent inhibitors of hypoxia (1% O_2_)-induced HIF-1 activation with IC_50_ values of 3.2 and 3.5 μM, respectively.^10^ A new alkyl benzoate, 4′-methylheptyl benzoate (12) isolated from *H. erectus* displayed significant cytotoxic activity against breast adenocarcinoma (MCF-7) with IC_50_ value of 2.4 μM (ref. 11) Fig. 2.

A new mero sesquiterpene, 19-methoxy-9,15-ene-puupehenol (13) was active at 1.78 μM on scavenger receptor-Class B type 1 HepG2 (SR-B1 HepG2) stable cell lines. So, it can be considered a target for treating atherosclerosis disease.^12^ Petrosaspongiolactam A (14), a new sesterterpene lactam isolated from *Petrosaspongia* sp. was reported to inhibit of TDP-43 binding tendency to its DNA target strand and was more active than petrosaspongiolactam B.^8^

From the sponge *Smenospongia aurea*, smenamides A (15) and B (16) were isolated and showed potent cytotoxic activity at nanomolar concentrations against lung cancer Calu-1 cells, where compound (15) exerted its activity through a clear pro-apoptotic mechanism. The IC_50_ values for smenamides A and B were 8 nM and 49 nM, respectively. This makes smenamides promising leads for antitumor drug design.^13^ Toward the synthesis of smenamide A (15), a study reported the synthesis of two stereoisomers named *ent*-smenamide A and 16-*epi*-smenamide. This study also established the previously unknown relative and absolute configurations of smenamide A (15).^14^ Another study discussed the synthesis of 16-*epi*-analogue of smenamide A and eight analogues in the 16-*epi* series to determine the role of the structure activity relationship features. The synthetic analogues were tested on multiple myeloma (MM) cell lines and the results showed that the configuration at C-16 slightly affects the activity, since the 16-*epi*-smenamide A was still active at nanomolar concentrations. Interestingly, it has been found that the analogue which contains the pyrrolinone terminus, was inactive while the analogue which composed of the intact C12–C27 portion, retained its activity, even though its EC_50_ value was 1000 times smaller compared with the parent 16-*epi*-smenamide A. In addition, this analogue was found to be able to block the cell cycle at the *G*_0_/*G*_1_ phase.^15^

Also from the same species (*S. aurea*), smenothiazoles A (17) & B (18) were isolated and showed potent cytotoxic activity at nanomolar concentrations on four different cell lines (Calu-1 and LC31 lung-cancer cells, A2780 ovarian cancer cells and MCF7 breast cancer cells). The tumor cells were treated for 48 h with different concentrations of the two compounds. After this time, the cells appeared elongated and flattened under the optical microscope. Also, some cells showed small vacuoles in their cytoplasm. All this indicated cell death. These morphological changes were observed at concentrations ≥50 nM. Moreover, at 100 nM, all cells in the suspension were dead. In contrast, the morphology of treated cells was similar to that of untreated cells at concentrations of 1, 10 and 30 nM.^16^ Later in 2017, smenothiazole A (17) was isolated from the sponge *Consortium plakortis* symbiotica–*Xestospongia deweerdtae*. The anti-tuberculosis activity of smenothiazole A (17) was *in vitro* evaluated against *Mycobacterium tuberculosis* H37Rv and the results showed that smenothiazole A (17) exhibited significant activity with MIC value of 4.1 μg mL^−1^. Synthesis and subsequent biological screening of a dechlorinated analogue of smenothiazole A, revealed that the chlorine atom is necessary for the anti-TB activity.^17^ Also in 2017, total synthesis of smenothiazole A (17) was reported. In this reaction, silastannation, Stille reaction and carefully controlled desilylchlorination were appointed as key steps to construct the unique polyketide acid fragments. The optimized reaction conditions avoided the migration of 2,5-diene to a 2,4-conjugated system.^18^

A new sesquiterpene (+)-5-*epi*-20-*O*-ethylsmenoquinone (19) was reported from *S. aurea* and *S. cerebriformis*. This compound exhibited inhibition tumor growth at 0.75 and 1.5 μM against SW480 (IC_50_ = 3.24 μM) and HCT116 cells (IC_50_ = 2.95 μM), respectively.^19^ The antiproliferative activity of smenolactones A, C and D (20–22), polyketides isolated from *S. aurea*, were evaluated against three tumor cell lines. The antiproliferative activity against MCF-7 breast adenocarcinoma cells was observed at low-micromolar (1 μM) or sub-micromolar concentrations.^20^ From *S. cerebriformis*, a new naphtoquinone smenocerone B (23) was reported and significantly exhibited cytotoxic activity against hepatocellular carcinoma (HepG-2), promyelocytic leukemia (HL-60) and breast carcinoma (MCF-7) human cancer cells with IC_50_ values of 3.2 ± 0.2, 4.0 ± 0.7 and 4.1 ± 0.8 μg mL^−1^, respectively.^21^

**Fig. 3:**
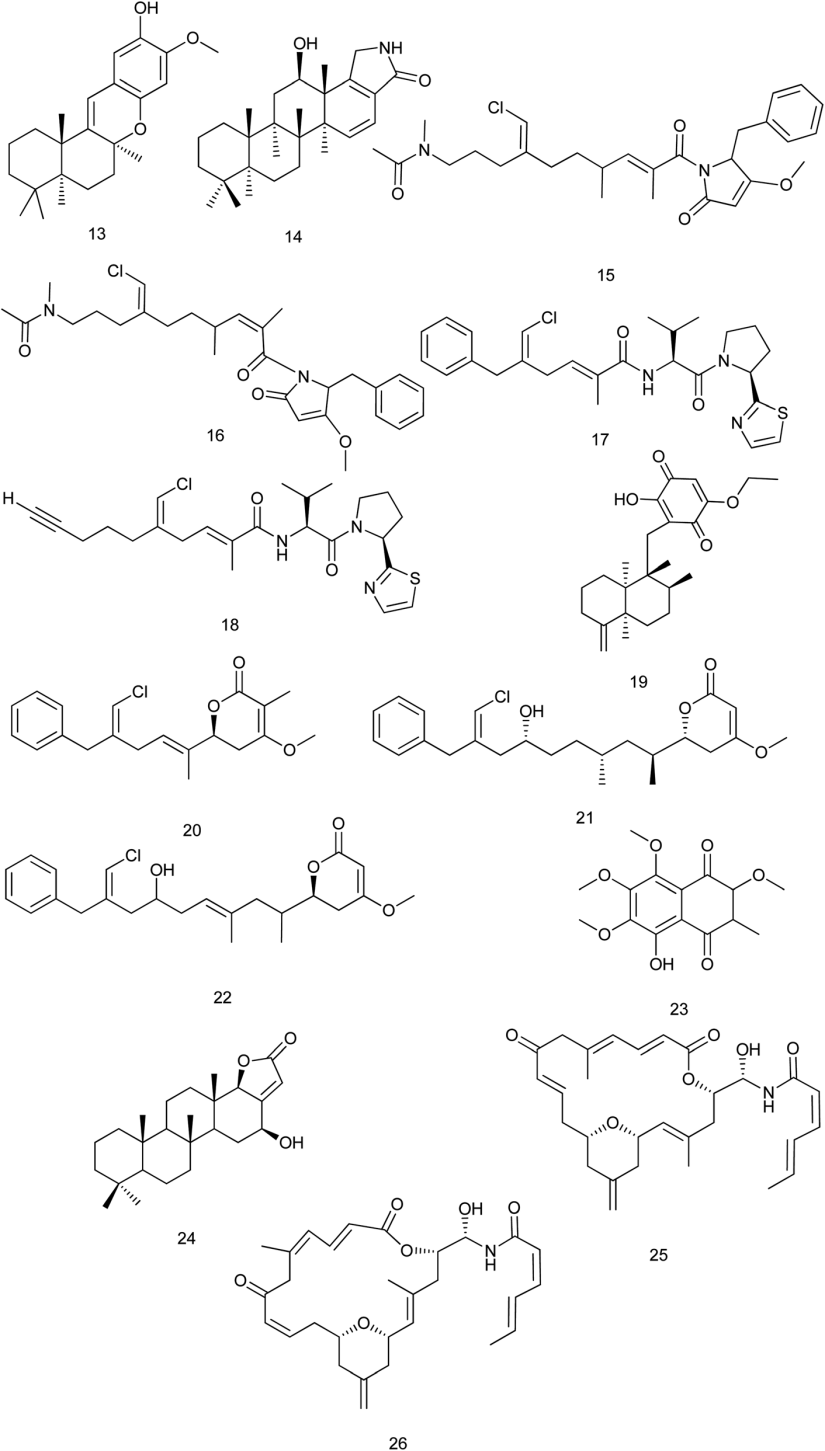
Structures of compounds (13–26).

From the marine sponge *Scalarispongia* sp., four new scalarane sesterterpenoids were isolated. Cytotoxicity was evaluated against six human cancer cell lines and only compound (24) exhibited a significant cytotoxicity against breast (MDA-MB-231) cell line with (GI_50_ values down to 5.2 μM).^22^ Zampanolides B and C (25, 26) are new macrolides which were isolated from *Cacospongia mycofijiensis*. They were found to have a potent nanomolar cytotoxic effect toward the HL-60 cell line with IC_50_ of 3.3 and 3.8 nM, respectively^23^Fig. 3.

From *Phyllospongia lamellosa*, phyllospongins A, C, D and E (27–30) were isolated and investigated for their cytotoxic activity against human cancer cell lines (HePG-2, MCF-7, and HCT-116). All exhibited significant cytotoxicity towards the three cell lines with IC_50_ down to 2.14 mg mL^−1^ except phyllospongin B. Also, they were evaluated for their antimicrobial activity against some Gram-positive and Gram-negative strains. Results showed that phyllospongins D and E significantly inhibited the growth of *S. aureus* with IC_50_ of 1.9 and 2.5 μg mL^−1^ and *Bacillus subtilis* with IC_50_ values of 1.7 and 3.3 μg mL^−1^, respectively.^24^

Two new homoscalarane sesterterpenes (31) and (32) were isolated from a sponge, closely similar to *Carteriospongia* sp., designated NB-04-06-17 displayed submicromolar antiproliferative effect against the A2780 ovarian cell line with IC_50_ values of 0.26 and 0.28 μM, respectively.^25^ From the same species, 12β-(3′β-hydroxybutanoyloxy)-20,24-dimethyl-24-oxo-scalara-16-en-25-al (33) and 12β-(3′β-hydroxypentanoyloxy)-20,24-dimethyl-24-oxo-scalara-16-en-25-al (34) were isolated. They exhibited significant cytotoxic activity against different cancer cell lines (leukemia, lymphoma, oral, colon and breast cancer cell lines) with an IC_50_ range of 0.01 to 3.17 μg mL^−1^. The results suggested that the proapoptotic effect of (33, 34) was mediated through the inhibition of Hsp90 and topoisomerase activities.^26^ A new scalarane sesterterpene, carteriofenone D (35), was isolated from *C. foliascens* and exhibited significant cytotoxicity against P388, HT-29, and A549 cell lines with the IC_50_ values of 0.96, 1.43 and 3.72 μM, respectively. This compound was also found to display brine shrimp lethality towards *Artemia salina* with an LC_50_ value of 5.80 μM. Also, it exhibited antifouling activity against the larval settlement of the barnacle *Balanus amphitrite* with an IC_50_ value of 2.50 μM.^27^

**Fig. 4:**
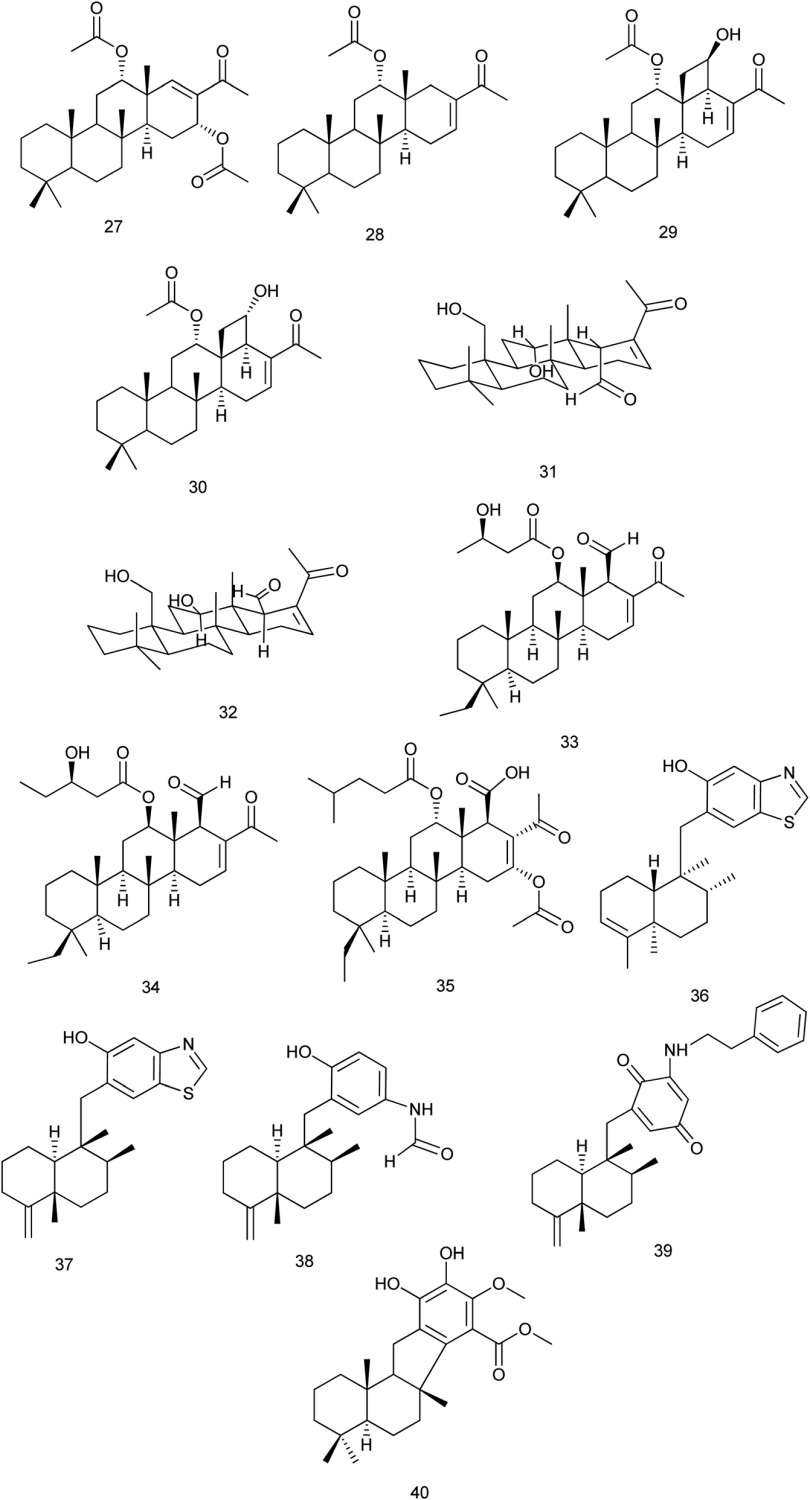
Structures of compounds (27–40).

Anti-inflammatory evaluation of compounds dactylospongins A, B and D (36), (37), (38), dysidaminone N (39), and 19-*O*-methylpelorol (40), which were isolated from *Dactylospongia* sp., showed inhibitory effect on the production of IL-6, IL-1β, IL-8, and PEG2 with IC_50_ values of 5.1–9.2 μM, respectively^28^Fig. 4.

5-*epi*-Nakijiquinones S, Q, T, U and N, new sesquiterpene aminoquinones (41–45) were isolated from *Dactylospongia metachromia* and showed a significant cytotoxic activity against the mouse lymphoma cell line L5178Y with IC_50_ values ranging from 1.1 to 3.7 μM. 5-*epi*-Nakijinols C and D (46, 47) exhibited the strongest inhibitory effect against ALK, FAK, IGF1-R, SRC, VEGF-R2, Aurora-B, MET wt, and NEK6 kinases (IC_50_: 0.97–8.62 μM).^29^

From the marine sponge *Fascaplysinopsis reticulate*, two new tryptophan derived alkaloids 6-bromo-8,10-dihydro-isoplysin A (48) & 5,6-dibromo-8,10-dihydro-isoplysin A (49) were reported. These compounds exhibited an antimicrobial activity towards *Vibrio natrigens* with MIC values of 0.01 and 1 μg mL^−1^, respectively.^30^ According to ref. 31, a new bromotyrosine-derived alkaloid subereamolline D (50) was isolated from the same species together with other new alkaloids. Among them, subereamolline D exhibited cytotoxic activity against Jurkat cell lines with IC_50_ value of 0.88 μM.

**Fig. 5:**
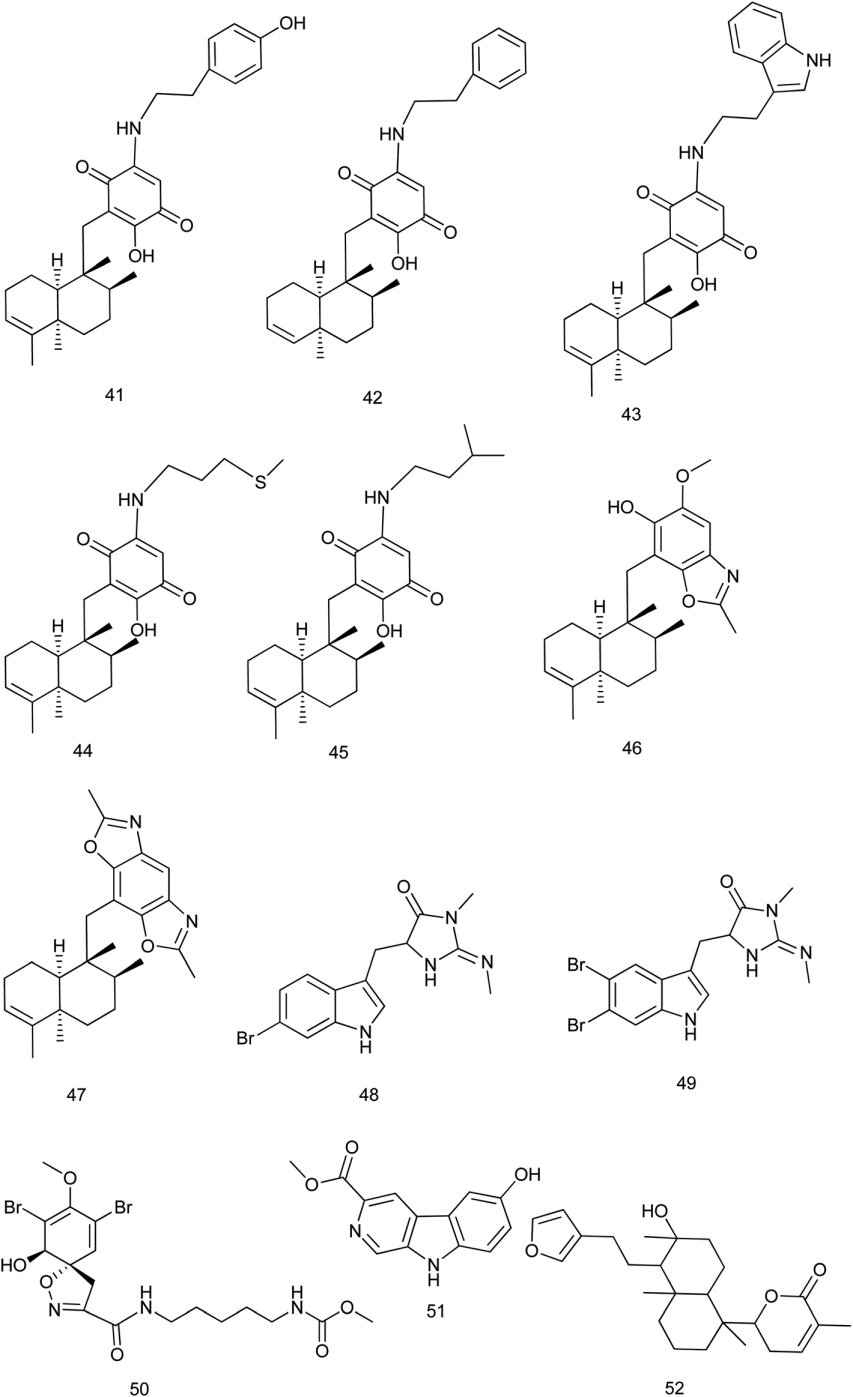
Structures of compounds (41–52).

From the Indonesian marine sponge *Luffariella variabilis*, a new β-carboline alkaloid, variabine B (51) was isolated. Variabine B was proven to inhibit chymotrypsin-like activity of the proteasome and Ubc13 (E2) and Uev1A interaction with IC_50_ values of 4 and 5 μg mL^−1^, respectively.^32^ From the same species, 6-(5-(2-(furan-3-yl)ethyl)-6-hydroxy-1,4*a*,6-trimethyldecahydronaphthalen-1-yl)-3-methyl-5,6-dihydro-2*H*-pyran-2-one (52), a new sesterterpene was isolated and evaluated for its cytotoxic activity against cultured NBT-T2 cells through MTT assay. This compound showed a significant cytotoxic activity at IC_50_ of 1.0 μM (ref. 33) Fig. 5.

### 2.2 Potent bioactive compounds isolated from family Dysideidae

During 2013–2019, 73 new and 47 known compounds were reported from different genera of family Dysideidae. From *Dysidea avara*, dysiquinol D (53), a new sesquiterpene quinol, and (5*S*,8*S*,9*R*,10*S*)-19-ethoxyneoavarone (54), a new sesquiterpene quinone were isolated. They exhibited modest cytotoxicity against human myeloma cancer cell line NCI-H929 with IC_50_ values of 2.81 and 2.77 μM, respectively. In addition, dysiquinol D (53) also showed inhibitory activity against NF-kB with IC_50_ value of 0.81 μM, which was more potent compared to the positive control rocaglamide.^34^ A new nitrogenous azirine derivative compound, debromoantazirine (55) was isolated from *Dysidea* sp. Evaluation of its cytotoxic activity showed high cytotoxicity against NBT-T2 cells with IC_50_ value of 4.7 μg mL^−1^.^35^

**Fig. 6:**
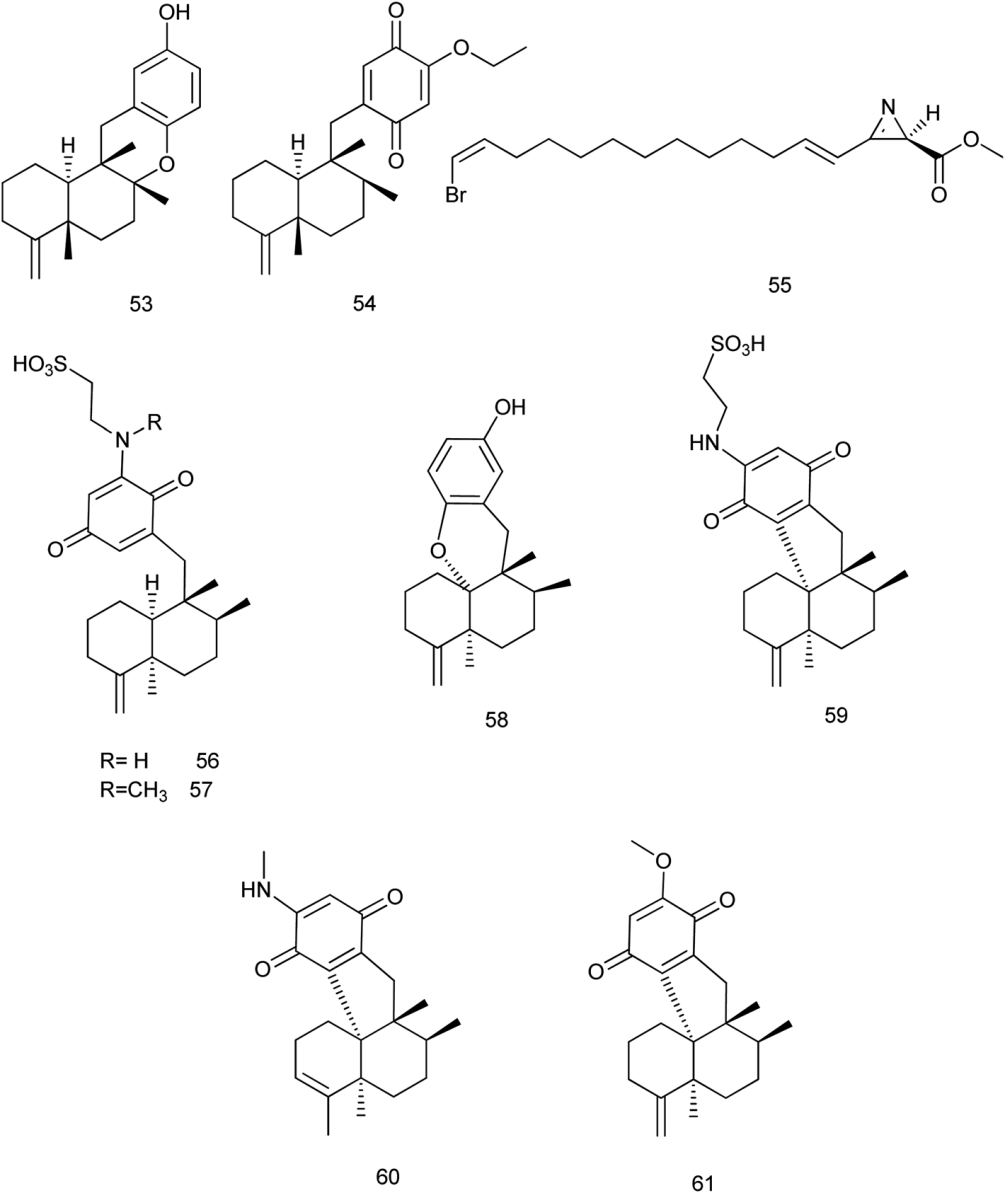
Structures of compounds (53–61).

Six new meroterpenoids (56–61), were isolated from *Dysidea* sp. Aureol B (58) exhibited modest cytotoxic activity against K562 cell line with IC_50_ value of 4.8 μM where, melemeleone C (56) exhibited modest activity against A549 cell line with IC_50_ value of 4.2 μM. Other meroterpenoids (57, 59–61) showed significant cytotoxicity against the two cell lines with range of (0.7–4.9 μM)^36^Fig. 6.

The chemical investigation of the marine sponge *Dysidea* sp. afforded eight new sesquiterpene derivatives. Among them, smenospongimine (62) which is a sesquiterpene aminoquinone showed a potent inhibitory effect against *B. subtilis* (6633), *S. aureus* (25 923) and *Escherichia coli* (25 922) with MIC values of 3.125, 3.125 and 6.25 μg mL^−1^, respectively.^37^

From the same species, two new sterols dysiroids A and B (63, 64) were isolated and reported for their antibacterial activities against different bacterial strains. Dysiroid A exhibited its activity against *S. aureus* ATCC 29213 and *Enterococcus faecalis* ATCC 29212 with MIC of 4 μg mL^−1^ where, dysiroid B exhibited its inhibitory effect against *S. aureus* ATCC 29213 and ATCC 43300 and *E. faecalis* ATCC 29212 with the same MIC value.^38^ Chemical investigations of the South China Sea marine sponge *D. fragilis* revealed the isolation of thirteen new sesquiterpene aminoquinones dysidaminones A–M. Among these compounds, dysidaminones C (65), E (66), and J (67) showed cytotoxic activity against human NCI-H929 myeloma, HepG2 hepatoma, and SK-OV-3 ovarian cancer cell lines with IC_50_ values of 0.45–5.25 μM. In addition, dysidaminones J (67) and H (68) exhibited cytotoxicity against mouse B16F10 melanoma with IC_50_ values of 2.45 and 1.43 μM, respectively. These four cytotoxic compounds also exhibited NF-kB inhibitory effect with IC_50_ values of 0.11–0.27 μM.^39^ Later in 2018, dysidaminone H (68) was investigated for its cytoprotective effect. It was examined against hydrogen peroxide (H_2_O_2_)-induced oxidative injury in human keratinocyte cell line. The results showed that dysidaminone H resisted H_2_O_2_ induced decline of cell viability through inhibition of the accumulation of ROS and suggested that dysidaminone H might be the candidate therapeutic drug for skin diseases which caused by oxidative injury.^40^

**Fig. 7:**
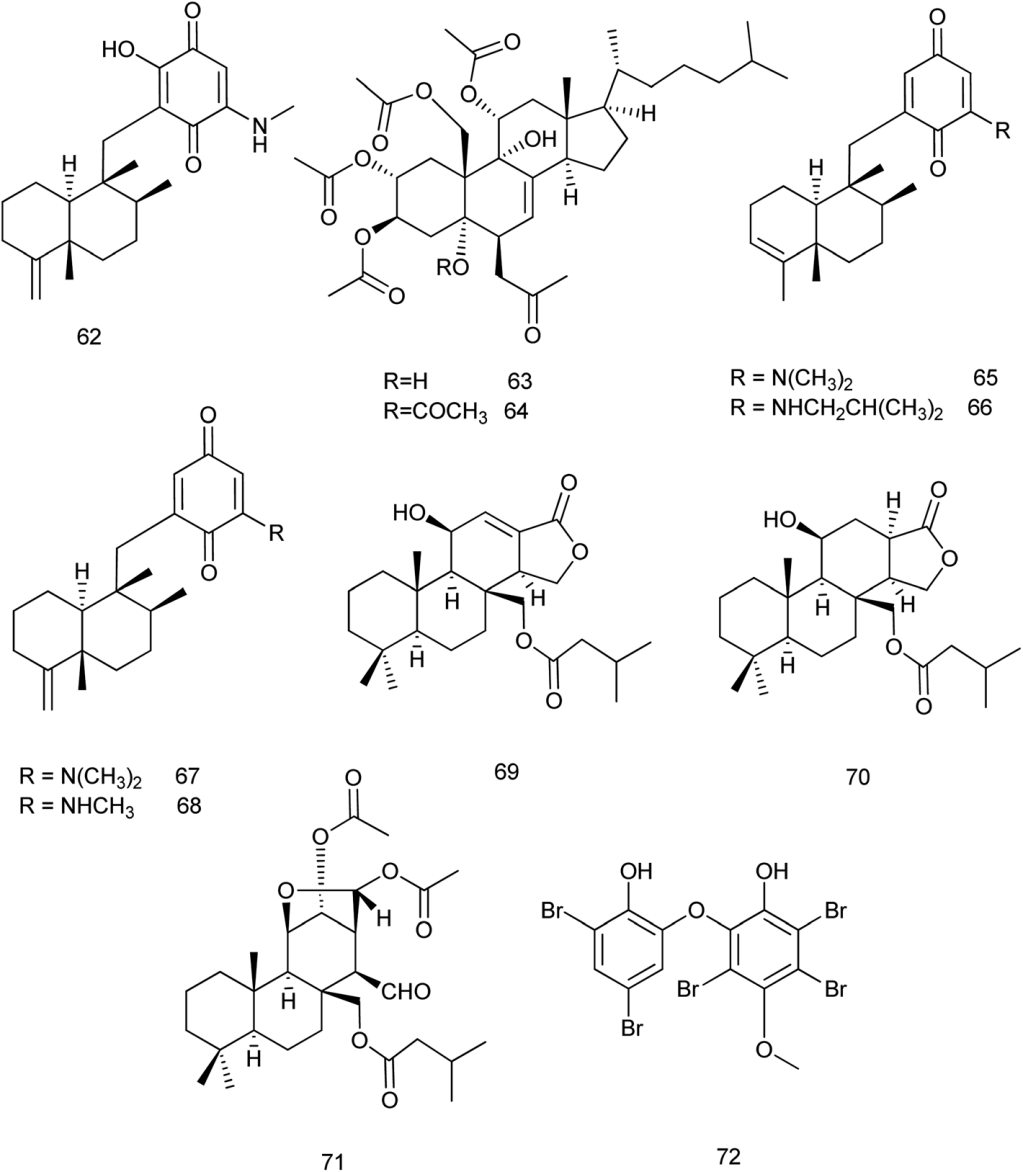
Structures of compounds (62–72).

Chemical analysis of the sponge *Dysidea* cf. *arenaria* revealed the presence of three new diterpenes which were evaluated for their cytotoxic effects. The cytotoxicity of compounds 69, 70 and 71 against NBT-T2 cells was evaluated and their IC_50_ values were 3.1, 1.9 and 3.1 μM, respectively.^41^ One new methoxy-poly brominated compound (72) was isolated together with other known polybrominated compounds from *Lamellodysidea* sp. This compound exhibited a potent antimicrobial activity with low-to sub-microgram mL^−1^ minimum inhibitory concentrations against four strains of *S. aureus* and *E. faecium* with IC_50_ values of 0.39–3.1 μg mL^−1^ (ref. 42) Fig. 7.

### 2.3 Potent bioactive compounds isolated from family Spongiidae

Reviewing published data within 2013–2019 revealed that 70 new and 36 known compounds were reported from the family Spongiidae. The new sesquiterpene quinone, 20-demethoxy-20-methylaminodactyloquinone D (73), that was isolated from *Spongia pertusa* Esper; showed CDK-2 affinity with a *K*_d_ value of 4.8 μM. It was considered as the first example of a sesquiterpene quinone derived from a marine source with CDK-2 affinity which requires further functional investigations on CDK-2.^43^

From *Hippospongia* sp., a new sesterterpene named hippospongide C (74) was isolated. Cytotoxic activity was evaluated against different cell lines and the results showed that hippospongide C exhibited a significant cytotoxic activity against T-47D and K562 cell lines with IC_50_ values of 4.1 and 2.9 μg mL^−1^, respectively.^44^ Hippolachnin A (75), a polyketide with an unprecedented carbon skeleton of a four-membered ring, was isolated from the South China Sea sponge *H. lachne*. It was reported that hippolachnin A exhibited potent antifungal activity against three different pathogenic fungi, *Cryptococcus neoformans*, *Trichophyton rubrum*, and *Microsporum gypseum*, with a MIC value of 0.41 μM for each fungus.^45^ Later in 2017, synthetic method of (±)-hippolachnin A was reported.^46^ Also, a diversifiable and scalable synthesis of (±)-hippolachnin A was definitely described according to Thiocarbonyl Ylide Chemistry.^47^

In 2018, synthesis of hippolachnin A was reported taking into consideration structure–activity relationship studies. This study revealed that, in contrast to initial studies, hippolachnin A and several structural analogues lack activity against pathogenic fungi.^48^ Also, a unified strategy was reported for divergent synthesis of different types of polyketides. This strategy enabled total synthesis of the enantioselective form of (+)-hippolachnin A based on a series of bio-inspired, rationally designed transformations.^49^ In 2019, synthesis of the racemic (±)-hippolachnin, which serves as a platform for the synthesis of bioactive analogues was reported. Biological testing of this synthetic material did not confirm the previously reported antifungal activity of hippolachnin A but resulted in a moderate activity against nematodes and microbes.^50^

Also, from *H. lachne*, a pair of enantiomeric sesterterpenoids, (−) & (+) hippolide J (76 and 77), were isolated. The evaluation of the antifungal activity of (−) and (+) enantiomers revealed that both showed potent antifungal activity against three strains of hospital-acquired pathogenic fungi, namely, *C. albicans* SC5314, *C. glabrata* 537, and *Trichophyton rubrum* Cmccftla, with IC_50_ values of 0.125–0.25 μg mL^−1^.^51^

**Fig. 8:**
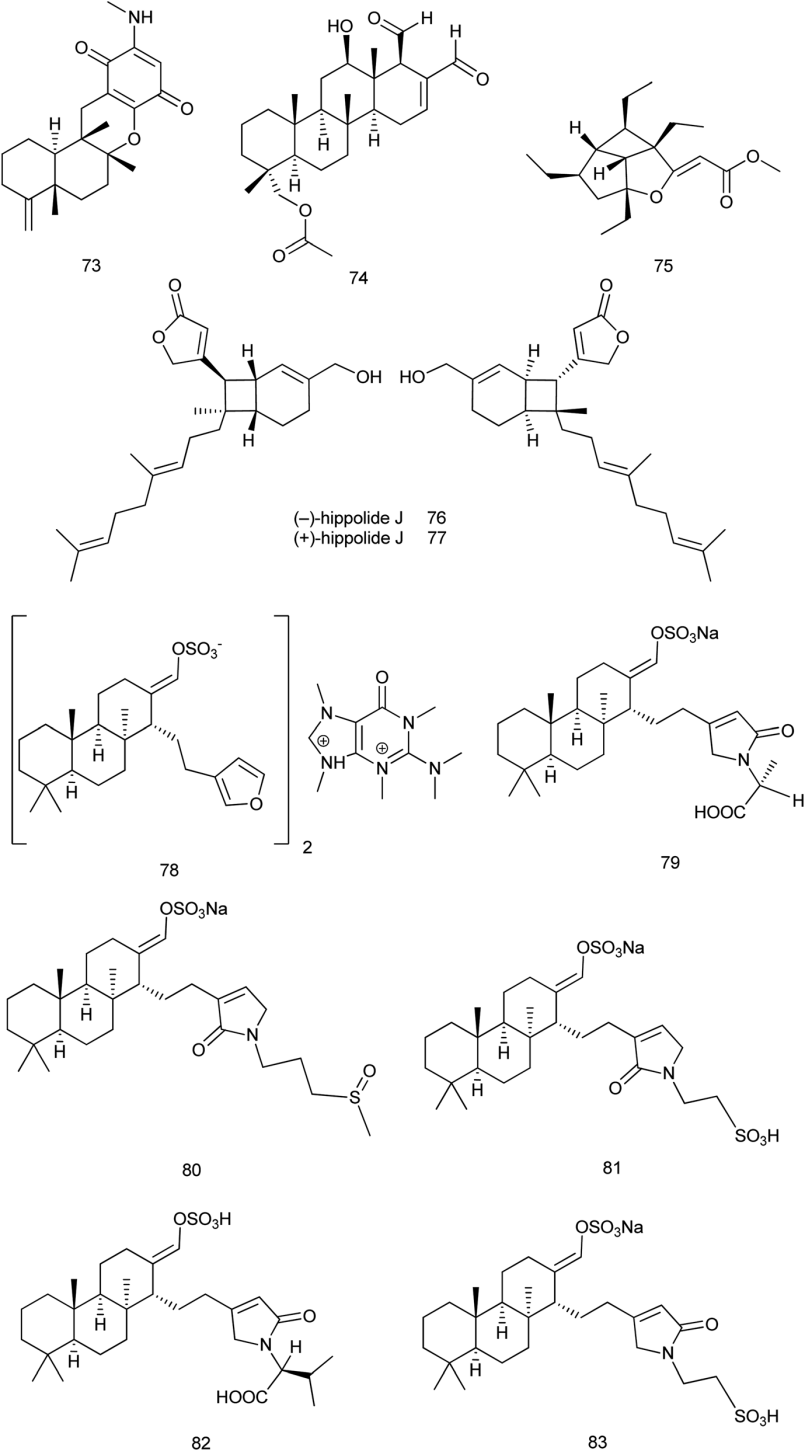
Structures of compounds (73–83).

From *Coscinoderma* sp., six new sesterterpenes (78) and coscinolactam C–G (79–83) classified as suvanines were isolated. All suvanines exhibited significant cytotoxic activities against the K562 cell line with IC_50_ range of 1.9–4.6 μM and all of them except coscinolactam D (80) showed potent activity towards the A549 cell line with IC_50_ range of 1.1–3.5 μM. Additionally, the new suvanine salt (78) had significant antibacterial activity towards *B. subtilis* (ATCC 6633) and *Salmonella enterica* (ATCC14028) with IC_50_ values of 0.78 μg mL^−1^ (ref. 52) Fig. 8.

### 2.4 Potent bioactive compounds isolated from family Irciniidae

New and known compounds which were isolated within 2013–2019 from the family Irciniidae are nearly equal (35 and 32, respectively). A new 9,11-secosterol (84) with the 2-ene-1,4-dione moiety was isolated from the marine sponge *Ircinia* sp. and this compound was evaluated for its antibacterial activity against different bacterial strains. The most significant activity was detected against *Micrococcus lutes* ATCC 9341 with the IC_50_ value of 3.1 μg mL^−1^.^53^ From the sponge *Ircinia felix*, a new 24-homoscalarane sesterterpenoid, felixin F (85) was isolated. The cytotoxicity of felixin F against the proliferation of a limited type of tumor cell lines was evaluated and the results showed modest cytotoxicity towards the leukemia K562, MOLT-4, and SUP-T1 cell lines with IC_50_ values of 1.27, 2.59 and 3.56 μM, respectively.^54^

**Fig. 9:**
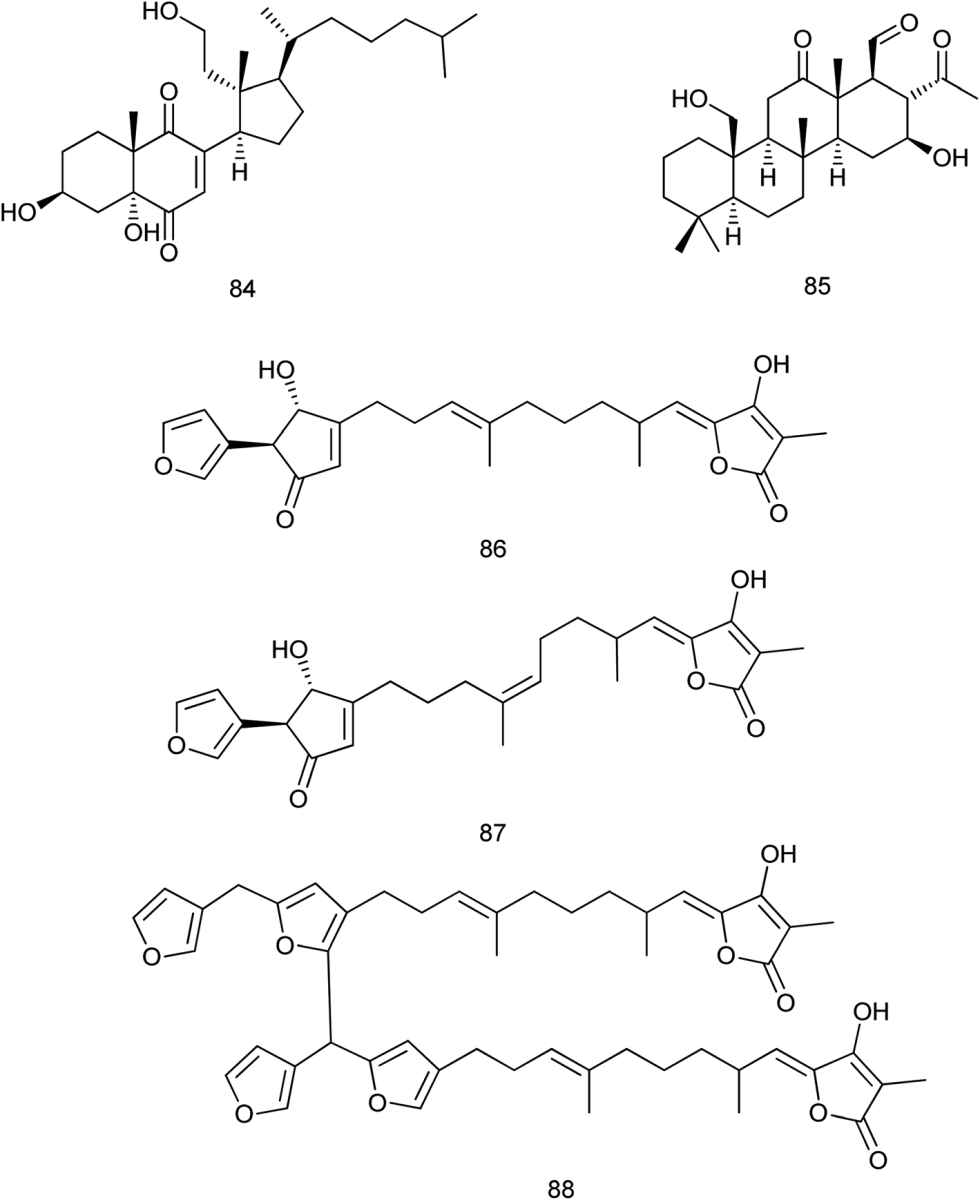
Structures of compounds (84–88).

Three new furanosesterterpene tetronic acids, sulawesins A–C (86–88), were isolated from a *Psammocinia* sp. marine sponge. These isolated compounds were proved to inhibit the deubiquitinating enzyme USP7, which could be considered as an emergent target of cancer therapy, with IC_50_ values in the range of 2.7–4.6 μM (ref. 55) Fig. 9.

It is noteworthy that no available researches were traced concerning the fifth family (Verticilliitidae) of this order. Additionally, several compounds were produced as artifacts (89–100) through intended reaction or unintended procedures.

Two derivatives α-16-methoxyfuroscalarol and β-16-methoxyfuroscalarol (89, 90) were obtained through the unintended chemical transformation of furoscalarol which was isolated from *Scalarispongia* sp. It could be postulated that the slightly acidic character of silica easily induced the formation of an oxocarbenium, which was followed by nucleophilic conjugate addition of methanol. The formation of an α-product (89) was likely favored because of the approach of the nucleophile toward the pseudoaxial direction for maximum overlap with the p-orbital.^56^ Another three sesquiterpenes (91–93) were isolated as solvent-generated artifacts from *Spongia pertusa* Esper because the extraction was processed with ethanol as a solvent.^43^

On the other hand, four ester derivatives [acetyl (94), butyryl (95), hexanoyl (96), and benzoyl (97)] were prepared from 2-(30,50-dibromo-20-methoxyphenoxy)-3,5-dibromophenol and their activities were evaluated against PTP1B and two cancer cell lines in order to investigate the structure–activity relationship features. Although compounds 94–97 exhibited potent inhibitory activities against PTP1B with IC_50_ range of (0.62–0.97 μM), cytotoxicity against HCT-15 and Jurkat cells was observed as a similar efficacy to that of the parent compound.^57^

**Fig. 10:**
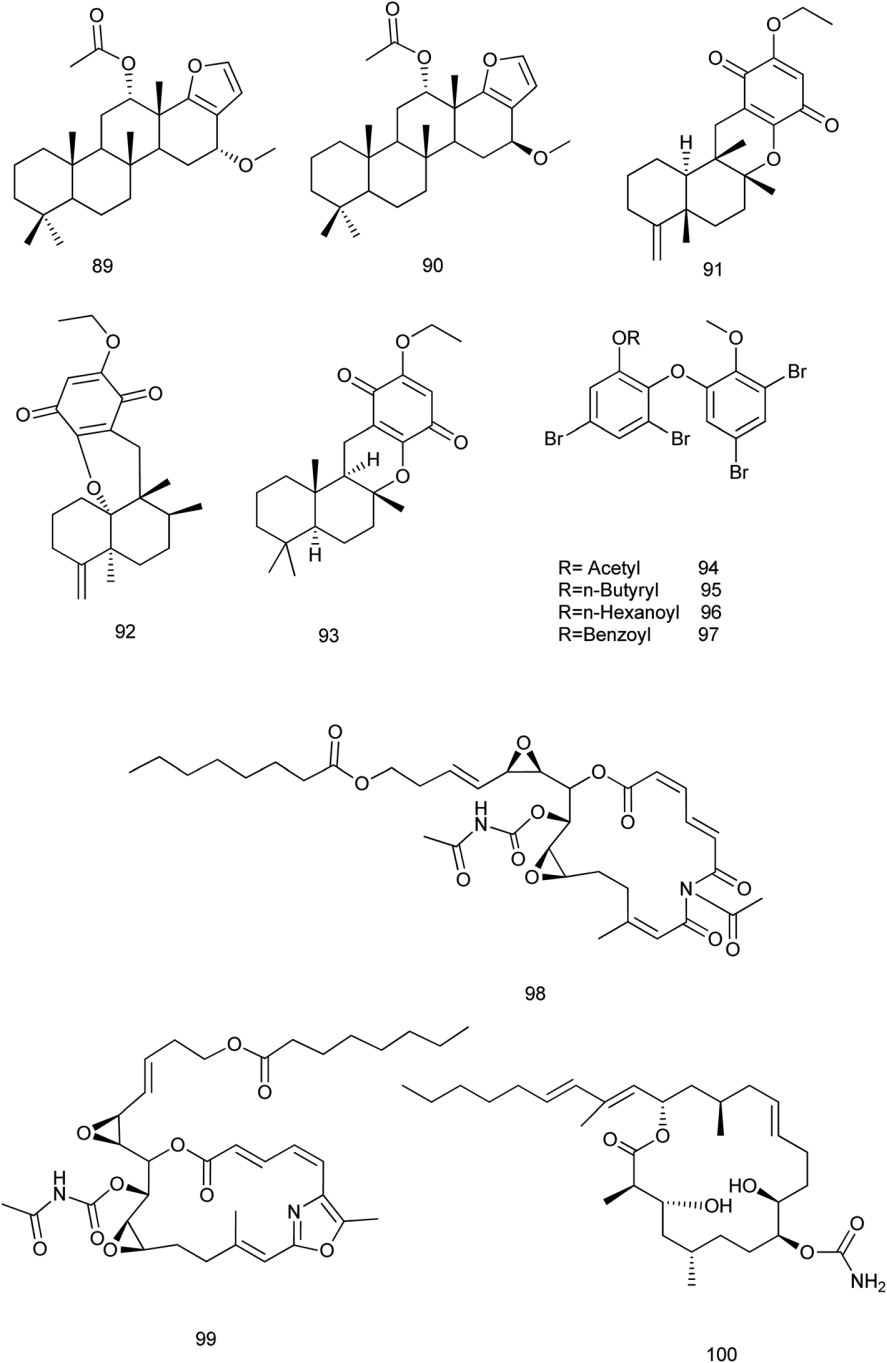
Structures of compounds (89–100).

Concerning *Fascaplysinopsis* sp., three new cytotoxic sponge derived nitrogenous macrolides salarin A (98), salarin C (99) and tulearin A (100), were prepared and evaluated as inhibitors of K562 leukemia cells. A preliminary structure–activity relationship studies revealed that the most sensitive functional groups were the 16,17-vinyl epoxide in both salarins, the triacylamino group in salarin A and the oxazole in salarin C (less sensitive). Regioselectivity of reactions was also found for tulearin A^58^Fig. 10.

Later in 2015, salarin C was evaluated for its activity against chronic myeloid leukemia (CML) cells, especially after their incubation in atmosphere at 0.1% oxygen. Salarin C induced mitotic cycle arrest, apoptosis and DNA damage. It also concentration-dependently inhibited the maintenance of stem cell potential in cultures in low oxygen of either CML cell lines or primary cells.^59^ In 2017, the synthesis of the eastern section of salarin C was reported employing a halogen dance reaction to assemble the trisubstituted oxazole moiety. The synthesis was inspired according to Kashman's hypothesis on the biomimetic oxidation of salarin C to salarin A.^60^

In 2018, a study toward the total synthesis of salarin C was reported. This strategy based on the synthesis of dideoxysalarin C as a potential intermediate for the total synthesis of the marine macrolide salarin C. The macrolactone core of dideoxysalarin C was assembled by Suzuki coupling between alkyl iodide and vinyl iodide and Shiina macrolactonization as key steps in the total synthesis. All macrocyclic intermediates during the synthesis were found to be of limited stability.^61^ Also, the synthesis of the macrocyclic core of salarin C was described using two epoxides being replaced by alkene moieties. In the key step, ring-closing metathesis exclusively afforded the (E)-product. Additionally, the trisubstituted oxazole unit embedded in the 17-membered ring underwent photo-oxidation on treatment with singlet oxygen, giving macrocyclic trisacylamines.^62^

Concerning tulearin A, according to ref. 63 an application to the total synthesis of tulearin A was reported. This application depended on the reaction of lactones with the reagent generated *in situ* from CCl4 and PPh3 in a Wittig-type fashion to give *gem*-dichloro-olefin derivatives. Such compounds can undergo reductive alkylation on treatment with organolithium reagents RLi to furnish acetylene derivatives. The reaction can be enhanced with either Cu(acac)_2_ or Fe(acac)_3_/1,2-diaminobenzene. Additionally, two alkynol derivatives were prepared using this way from readily accessible lactone precursors served as the key transformation steps for the total synthesis of the cytotoxic marine derived macrolides tulearin A and C Fig. 10.

## 3. Analysis of drug-like physicochemical properties

**Fig. 11:**
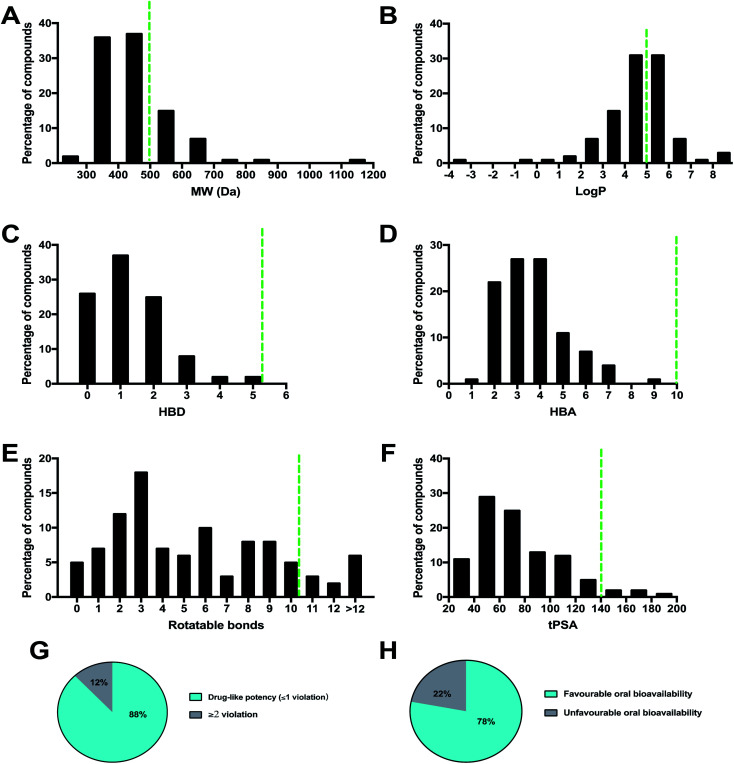
Analysis of drug-like physicochemical properties.

A necessary requirement for a drug is acceptable aqueous solubility and intestinal permeability. These properties are necessary but do not discriminate drugs from non-drugs.^64^ By calculating the physico-chemical properties, it is possible to predict the oral bioavailability of each compound. Lipinski's rule of 5 (Ro5) considers orally active compounds and defines four simple physico-chemical parameter ranges (molecular weight ≤ 500, log *P* ≤ 5, hydrogen bond donor ≤ 5, hydrogen bond acceptor ≤ 10) associated with 90% of orally active drugs that have achieved phase II clinical status. If a compound fails the Ro5, there is a high probability that oral activity problems will be encountered. However, passing the Ro5 is no guarantee that a compound is drug-like. Topological polar surface area (tPSA) is an additional descriptor that has been shown to correlate with passive molecular transport through membranes, and therefore allows prediction of transport properties of drugs. It was shown that PSA ≤ 140 Å and number of rotatable bonds ≤ 10 is an efficient and selective criterion for a drug-like molecule. The physico-chemical properties (MW, log *P*, HBD, HBA, rotatable bond, and tPSA) of the 100 MNP compounds in this review were calculated using the Instant JChem [Instant JChem 17.10.0, 2017 ChemAxon Ltd. (http://www.chemaxon.com)] of the molecules and projected onto a drug-like cutoff threshold of Lipinski's rules and Veber's oral bioavailability rule Fig. 11.

The molecular weight profile of the 100 MNP showed a wide range from 234.34 to 1138.58 Da. The majority of compounds (72%) are distributed between 300 Da and 500 Da Fig. 11A. Among the higher molecular weight compounds, clusters were observed around 600 Da and 700 Da, exemplified by tulearin A (100) and Dysiroid A (63) with MW of 535.77 and 690.87 Da, respectively. However, about 25% of compounds had a molecular weight greater than 500 Da, compound 78 (*N*,*N*-dimethyl-1,3-dimethylherbipoline salt) with molecular weight of 1138.58.

The log *P* histogram of the 100 MNP followed a normal distribution with the maximum around 4–6 and 58% of the compounds had a favorable log *P* value less than 5 Fig. 11B. Notably, compounds with large molecular weights were also found to show high log *P* values, such as sulawesin C with MW 819 and log *P* 11.06. Surprisingly, regarding to calculated HBA and HBD, all of 100 compounds introduced in this review were within the Lipinski-compliant region. The distribution of HBD Fig. 11C and HBA Fig. 11D showed a similar pattern. Overall, 45 of the 100 compounds did not violate Lipinski's rule of five, and 88 compounds had one violation or less. Around 78% of the compounds showed favorable oral bioavailability properties Fig. 11E.

## 4. Conclusion

**Fig. 12:**
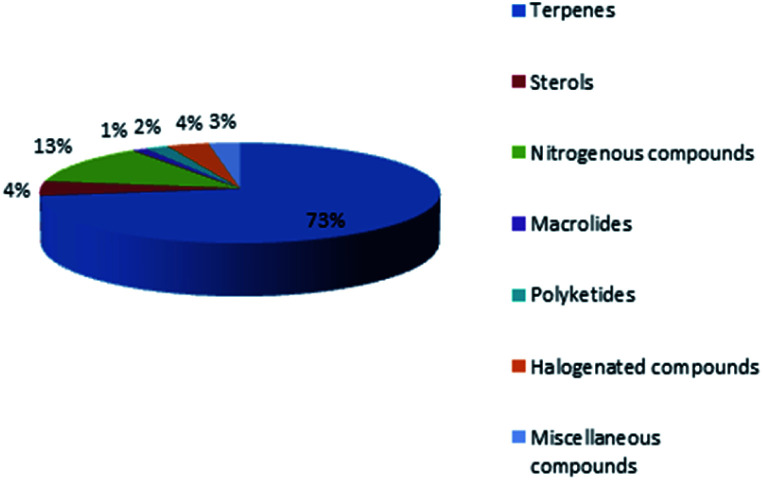
Different chemical classes of metabolites isolated from Dictyoceratida sponges.

**Fig. 13:**
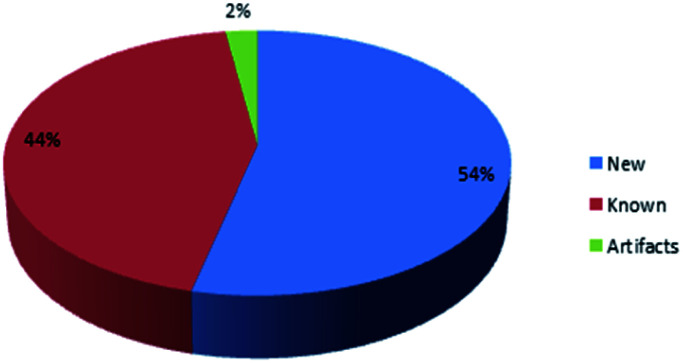
Percentages of new, known compounds and artifacts isolated from Dictyoceratida sponges.

Marine sponges and other marine lifes form have a very important role in drug discovery due to the diverse range of bioactive compounds which are isolated from different environmental and geographic conditions. The order Dictyoceratida (Phylum Porifera, Class Demospongiae, Order Dictyoceratida) consists of five families Thorectidae, Dysideidae, Spongiidae, Irciniidae and Verticilliitidae. Several diverse classes of secondary metabolites were reported within 2013–2019 period. Terpenes were the major reported chemical class consisting of 73% including simple terpenes, diterpenes, sesquiterpenes, sesterterpenes and other terpenes. The second major class was nitrogenous compounds consisting of 13% including alkaloids, alkaloids related and other nitrogenous compounds Fig. 12. These different classes can be summarized as 54% new isolated compounds compared to 44% known compounds Fig. 13.

**Fig. 14:**
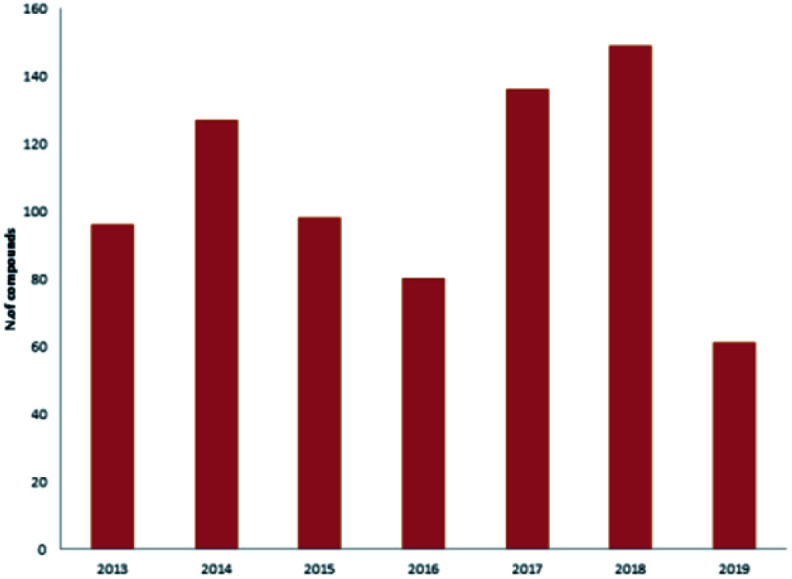
Number of compounds isolated from Dictyoceratida sponges per year.

**Fig. 15:**
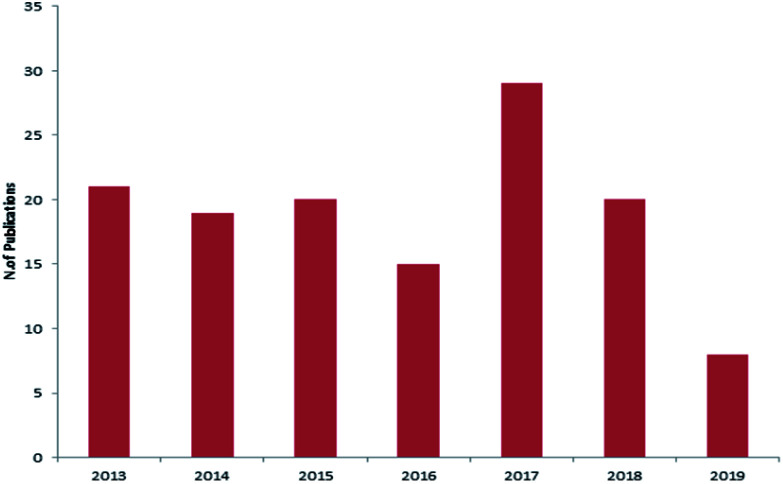
Number of publications per year.

Reviewing the published data and following the number of isolated compounds per year, Fig. 14 and 15 showed that 149 compounds were isolated in 2018 and published in 20 scientific papers whereas 136 compounds were isolated in 2017 and published in 29 papers.

**Fig. 16:**
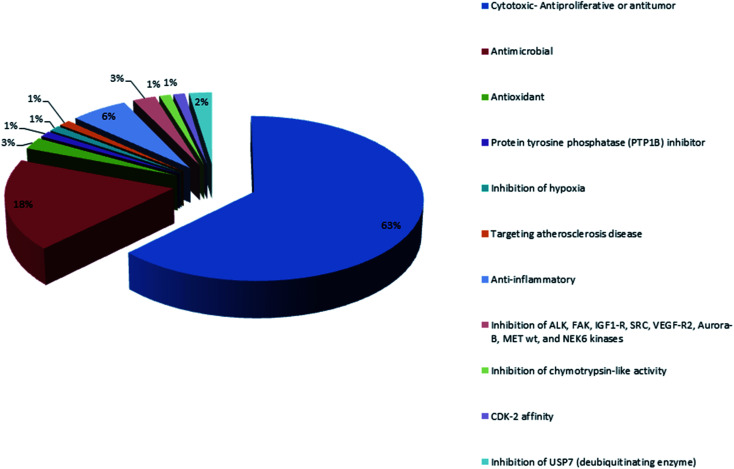
Biological activities of orally bioavailable compounds.

Analysis of the physicochemical properties of these 100 MNPs showed that 45 of the 100 compounds did not violate Lipinski's rule of five, and 88 compounds had one violation or less. The majority of compounds (72%) are distributed between 300 Da and 500 Da and 58% of the compounds had a favorable log *P* value less than 5. Interestingly, 78% of the compounds have favorable oral bioavailability properties for different biological activities which are demonstrated in Fig. 16. The majority of orally bioavailable compounds were reported to have cytotoxic, antitumor or anti-proliferative activities. The second major activity was the antimicrobial one. The increasing number of isolated bioactive compounds can provoke researchers for more investigations and studies of sponges belonging to the order Dictyoceratida.

## Conflicts of interest

There are no conflicts to declare.

## Supplementary Material

RA-010-D0RA04408C-s001
