# Supplementary material for: Marine natural products from sponges (Porifera) of the order Dictyoceratida (2013 to 2019); a promising source for drug discovery
Source: RSC Adv. 2020 Sep 21;10(57):34959–76. doi: 10.1039/d0ra04408c (PMC9056847; doi:10.1039/d0ra04408c)
Supplement: RA-010-D0RA04408C-s001 [file RA-010-D0RA04408C-s001.pdf]

Electronic Supplementary Material (ESI) for RSC Advances.
This journal is © The Royal Society of Chemistry 2020

Supplementary material

Marine natural products from sponges (Porifera) of the order Dictyoceratida (2013 to 2019); a promising source for drug discovery

Enas Reda Abdelaleem[a], Mamdouh Nabil Samy[a], Samar Yehia Desoukey[a], Miaomiao Liu[b], Ronald J Quinn[b], Usama Ramadan Abdelmohsen[a,c*]

[a]Department of Pharmacognosy, Faculty of Pharmacy, Minia University, 61519Minia, Egypt.

[b]Griffith Institute for Drug Discovery, Griffith University Brisbane, Queensland, 4111Australia

[c]Department of Pharmacognosy, Faculty of Pharmacy, Deraya University, Universities Zone, 61111 New Minia City, Minia, Egypt

Table (S1): Secondary metabolites isolated from dictyoceratida sponges and their biological tested activities (2013 to December 2019):

Compound	Class	Bioactivity	*Source*	Collection site	Ref	State
**A- Family: Thorectidae**						
Hyrtioerectine D	Nitrogenous compound	Antimicrobial, Antioxidant and cytotoxic	*Hyrtios sp.*	The Red Sea	[1]	New
Hyrtioerectine E	Nitrogenous compound	Antimicrobial, Antioxidant and cytotoxic	*Hyrtios sp.*	The Red Sea	[1]	New
Hyrtioerectine F	Nitrogenous compound	Antimicrobial, Antioxidant and cytotoxic	*Hyrtios sp.*	The Red Sea	[1]	New
Hyrtimomine A	Nitrogenous compound	Cytotoxic activity	*Hyrtios sp.*	Kerama Islands, Okinawa	[2]	New
Hyrtimomine B	Nitrogenous compound	NA	*Hyrtios sp.*	Kerama Islands, Okinawa	[2]	New
Hyrtimomine C	Nitrogenous compound	NA	*Hyrtios sp.*	Kerama Islands, Okinawa	[2]	New
Hyrtimomine D	Nitrogenous compound	Antimicrobial activity	*Hyrtios sp.*	Kerama Islands, Okinawa	[3]	New
Hyrtimomine E	Nitrogenous compound	Antimicrobial activity	*Hyrtios sp.*	Kerama Islands, Okinawa	[3]	New
Hyrtimomine F	Nitrogenous compound	Antimicrobial activity	*Hyrtios sp.*	Kerama Islands, Okinawa	[4]	New
Hyrtimomine G	Nitrogenous compound	Antimicrobial activity	*Hyrtios sp.*	Kerama Islands, Okinawa	[4]	New
Hyrtimomine H	Nitrogenous compound	Antimicrobial activity	*Hyrtios sp.*	Kerama Islands, Okinawa	[4]	New

Hyrtimomine I	Nitrogenous compound	Antimicrobial activity	*Hyrtios sp.*	Kerama Islands, Okinawa	[4]	New
Hyrtimomine J	Nitrogenous compound	Antimicrobial activity	*Hyrtios sp.*	Kerama Islands, Okinawa	[4]	New
Hyrtimomine K	Nitrogenous compound	Antimicrobial activity	*Hyrtios sp.*	Kerama Islands, Okinawa	[4]	New
Sesterstamide	Sesterterpene	Antileishmanial activity	*Hyrtios sp.*	Paracel Islands, China	[5]	New
(5bR,11aS,13R,13aS,13bR)-13-hydroxy-5b,8,8,11a,13a-pentamethyl-1,5,5a,6,7,7a,8,9,10,11,11a,11b,12,13,13a,13b-hexadecahydrochryseno[1,2-c]furan-3(5bH)-one	Sesterterpene	Cytotoxic activity	*Hyrtios sp.*	Paracel Islands, China	[5]	known
(4S,5bR,11aS,13R,13aS,13bR)-4,13-dihydroxy-5b,8,8,11a,13a-pentamethyl-5,5a,5b,6,7,7a,8,9,10,11,11a,11b,12,13,13a,13b-hexadecahydrochryseno[1,2-c]furan-1(4H)-one	Sesterterpene	Cytotoxic activity	*Hyrtios sp.*	Paracel Islands, China	[5]	known
16-epi-scalarafuran	Sesterterpene	Potent inhibitors of TDP-43 protein	*Hyrtios sp.*	Fiji Islands	[6]	New

Hemiacetal of 12-deacetyl-cis-24α,25α-dimethoxyscalarin	Sesterterpene	Potent inhibitors of TDP-43 protein	*Hyrtios sp.*	Fiji Islands	6	New
Scalarafuran	Sesterterpene	Potent inhibitors of TDP-43 protein	*Hyrtios sp.*	Fiji Islands	6	Known
			Petrosaspongia sp.	Fiji Islands	6	
			Hyrtios erectus	Similan Island, Thailand	7	
12-deacetyl-cis-24α,25α-dimethoxyscalarin	Sesterterpene	Potent inhibitors of TDP-43 protein	*Hyrtios sp.*	Fiji Islands	6	Known
12-deacetyl-trans-24α,25β-dimethoxyscalarin	Sesterterpene	Potent inhibitors of TDP-43 protein	*Hyrtios sp.*	Fiji Islands	6	Known
Heteronemin	Sesterterpene	Potent inhibitors of TDP-43 protein	*Hyrtios sp.*	Fiji Islands	6	Known
			Petrosaspongia sp.	Fiji Islands	6	
		Cytotoxic activity	*Hyrtios erectus*	The Red Sea Coast, Jeddah	8	
		Antitubercular & cytotoxic activities	*Hyrtios erectus*	The Red Sea, Egypt	9	
		Cytotoxic activity	*Hyrtios sp.*	Grand Island	10	
		Cytotoxic activity	*Hyrtios sp.*	Paracel Islands, China	5	
		Cytotoxic activity	*Hyrtios erectus*	The Red Sea, Egypt	11	

Heteronemin acetate	Sesterterpene	Potent inhibitors of TDP-43 protein	*Hyrtios sp.*	Fiji Islands	6	Known
12-Deacetyl-12-epi-deoxoscalarin	Sesterterpene	Potent inhibitors of TDP-43 protein	*Hyrtios sp.*	Fiji Islands	6	Known
12-epi-deoxoscalarin	Sesterterpene	Potent inhibitors of TDP-43 protein	*Hyrtios sp.*	Fiji Islands	6	Known
Scalarolide	Sesterterpene	Potent inhibitors of TDP-43 protein - cytotoxic activit	*Hyrtios sp.*	Fiji Islands	6	Known
		Anti-H. pylori& antitubercular activities	*Hyrtios erectus*	The Red Sea, Egypt	9	
			Hyrtios sp.	Paracel Islands, China	5	
		NA	*Hyrtios erectus*	The Red Sea, Egypt	11	
12-episcalaradial	Sesterterpene	Potent inhibitors of TDP-43 protein	*Hyrtios sp.*	Fiji Islands	6	Known

12-Deacetyl-12-*epi*-scalaradial	Sesterterpene	Potent inhibitors of TDP-43 protein - Anti-H. pylori-antitubercular-cytotoxic activities - Cytotoxic against hepatocellular carcinoma (HepG2), colorectal carcinoma (HCT-116) and breast adenocarcinoma cells (MCF-7).	*Hyrtios sp.*	Fiji Islands Red Sea	6	Known
			Hyrtios erectus	The Red Sea, Egypt	9	
			Hyrtios erectus	The Red Sea, Egypt	12	
12-Deacetyl-12,18-diepi-scalaradial	Sesterterpene	Potent inhibitors of TDP-43 protein	*Hyrtios sp.*	Fiji Islands	6	Known
Hyrtiosal	Sesterterpene	Potent inhibitors of TDP-43 protein	*Hyrtios sp.*	Fiji Islands	6	Known
			Petrosaspongia sp.	Fiji Islands	6	
Methyl diacarnoate A	Terpene	Potent inhibitors of TDP-43 protein	*Hyrtios sp.*	Fiji Islands	6	Known
Hyrtinadines C	Nitrogenous compound	Antimicrobial activity	*Hyrtios sp.*	Okinawa	13	New
Hyrtinadines D	Nitrogenous compound	Antimicrobial activity	*Hyrtios sp.*	Okinawa	13	New
Hyrtiolacton A	Meroterpene	NA	*Hyrtios sp.*	Yongxing island, South China Sea	14	New
Nakijinol F	Meroterpene	NA	*Hyrtios sp.*	Yongxing island, South China Sea	14	New

Nakijinol G	Meroterpene	Protein tyrosine phosphatase (PTP1B) inhibitory activity	*Hyrtios sp.*	Yongxing island, South China Sea	14	New
Nakijinol B	Meroterpene	NA	*Hyrtios sp.*	Yongxing island, South China Sea	14	Known
Dactyloquinone A	Meroterpene	NA	*Hyrtios sp.*	Yongxing island, South China Sea	14	Known
15α-Methoxypuupehenol	Merosesquiterpene	Cytotoxic due to antitumor effects against human glioblastoma (GBM) and breast cancer.	*Hyrtios sp.*	Haleiwa Trench, the North Shore of Oahu	15	Known
Hyrtiodoline A	Nitrogenous compound	Antitrypanosomal activity	*Hyrtios sp.*	Sharm el-Sheikh, Egypt.	16	New
4-Hydroxy-1H-indole-6-carboxylic acid methyl ester	Nitrogenous compound	NA	*Hyrtios sp.*	Sharm el-Sheikh, Egypt.	16	known
Dimer of indole-3-carbaldehyde	Nitrogenous compound	NA	*Hyrtios sp.*	Sharm el-Sheikh, Egypt.	16	known
3-Methylene hydroxy β-carboline	Nitrogenous compound	NA	*Hyrtios sp.*	Sharm el-Sheikh, Egypt.	16	known
2,3-Dibromo-4-hydroxybenzaldehyde	Halogenated compound	NA	*Hyrtios sp.*	Sharm el-Sheikh, Egypt.	16	known
Thorectidaeolide A	Sesterterpene	HIF-1 inhibitory activity	*Hyrtios communis*	The coast of Palau	17	New
4-Acetoxythorectidaeolide A	Sesterterpene	HIF-1 inhibitory activity	*Hyrtios communis*	The coast of Palau	17	New
Thorectidaeolide B	Sesterterpene	HIF-1 inhibitory activity	*Hyrtios communis*	The coast of Palau	17	New
Thorectidaeolide C	Sesterterpene	HIF-1 inhibitory activity	*Hyrtios communis*	The coast of Palau	17	New
Thorectidaeolide D	Sesterterpene	HIF-1 inhibitory activity	*Hyrtios communis*	The coast of Palau	17	New

Thorectidaeolide E	Sesterterpene	HIF-1 inhibitory activity	*Hyrtios communis*	The coast of Palau	[17]	New
Acantholide C	Sesterterpene	HIF-1 inhibitory activity	*Hyrtios communis*	The coast of Palau	[17]	Known
Acantholide B	Sesterterpene	HIF-1 inhibitory activity	*Hyrtios communis*	The coast of Palau	[17]	Known
16-Hydroxyluffariellolide	Sesterterpene	HIF-1 inhibitory activity	*Hyrtios communis*	The coast of Palau	[17]	Known
Acantholide A	Sesterterpene	HIF-1 inhibitory activity	*Hyrtios communis*	The coast of Palau	[17]	Known
Luffariellolide	Sesterterpene	HIF-1 inhibitory activity and antiproliferative activity	*Hyrtios communis*	The coast of Palau	[17]	Known
Hainanerectamine A	Nitrogenous compound	NA	*Hyrtios erecta.*	Hainan Province, China	[18]	New
Hainanerectamine B	Nitrogenous compound	Inhibitory activity against Aurora A	*Hyrtios erecta.*	Hainan Province, China	[18]	New
(−)-(1R*, 3R*)-6-hydroxy-1-(4-methylene-1H-imidazole)-2,3,4,9-tetrahydro-1H-β-carboline-3-carboxylic acid.	Nitrogenous compound	Inhibitory activity against Aurora A	*Hyrtios erecta.*	Hainan Province, China	[18]	Known
Hainanerectamine C	Nitrogenous compound	Inhibitory activity against Aurora A	*Hyrtios erecta.*	Hainan Province, China	[18]	New
5-Hydroxyindole-3-carbaldehyde	Nitrogenous compound	NA	*Hyrtios erecta.*	Hainan Province, China	[18]	Known
5-Hydroxyindole-3-carboxylic acid	Nitrogenous compound	NA	*Hyrtios erecta.*	Hainan Province, China	[18]	Known
5-Hydroxy-3-(2-hydroxyethyl)-indole	Nitrogenous compound	NA	*Hyrtios erecta.*	Hainan Province, China	[18]	Known
5-Hydroxytryptophan	Nitrogenous compound	NA	*Hyrtios erecta.*	Hainan Province, China	[18]	Known
Scalarinol	Sesterterpene	Cytotoxic activity	*Hyrtios erectus*	The Red Sea Coast, Jeddah	[8]	New
12-O-deacetyl-19-deoxyscalarin	Sesterterpene	Cytotoxic activity	*Hyrtios erectus*	The Red Sea Coast, Jeddah	[8]	known
5α, 8α-epidioxy-24-methylcholesta-6-en-3β-ol	Sterol	NA	*Hyrtios erectus*	The Red Sea Coast, Jeddah	[8]	known

12-Acetoxy,16-epi-hyrtiolide	Sesterterpene	Cytotoxic activity	*Hyrtios erectus*	The Red Sea, Egypt	11	New
		Anti-H. pylori-antitubercular-cytotoxic activities	*Hyrtios erectus*	The Red Sea, Egypt	9	
12 -Acetoxy,16 -methoxy,20 -hydroxy-17-scalaren-19,20-olide	Sesterterpene	Cytotoxic activity	*Hyrtios erectus*	The Red Sea, Egypt	11	New
12β,20-Dihydroxy-16β-acetoxy-17-scalaren-19,20-olide	Sesterterpene	Cytotoxic activity	*Hyrtios erectus*	The Red Sea, Egypt	11	known
Sesterstatin 7	Sesterterpene	Cytotoxic activity	*Hyrtios erectus*	The Red Sea, Egypt	11	known
		Anti-H. pylori-antitubercular-cytotoxic activities	*Hyrtios erectus*	The Red Sea, Egypt	9	
12-*epi*-24-deoxyscalarin	Sesterterpene	Cytotoxic activity	*Hyrtios erectus*	The Red Sea, Egypt	11	known
		Anti-H. pylori-antitubercular-cytotoxic activities	*Hyrtios erectus*	The Red Sea, Egypt	9	
Scalarolide acetate	Sesterterpene	Cytotoxic activity	*Hyrtios erectus*	The Red Sea, Egypt	11	known
		Anti-H. pylori-antitubercular-cytotoxic activities	*Hyrtios erectus*	The Red Sea, Egypt	9	
12-deacetyl-12,18-di-epi-scalaradial	Sesterterpene	Cytotoxic activity	*Hyrtios erectus*	The Red Sea, Egypt	11	known

24-Methoxypetrosaspongia C	Sesterterpene	Cytotoxic against hepatocellular carcinoma (HepG2), colorectal carcinoma (HCT-116) and breast adenocarcinoma cells (MCF-7).activity	*Hyrtios erectus*	The Red Sea, Egypt	12	New
		Anti-H. pylori-antitubercular-cytotoxic activity	*Hyrtios erectus*	The Red Sea, Egypt	9	
Sesterstatin 3	Sesterterpene	Cytotoxic against hepatocellular carcinoma (HepG2), colorectal carcinoma (HCT-116) and breast adenocarcinoma cells (MCF-7) activity.	*Hyrtios erectus*	The Red Sea, Egypt	12	Known
		Anti-H. pylori-antitubercular-cytotoxic activity			9	
12-Deacetyl-12,18-di-*epi*-scalaradial	Sesterterpene	Cytotoxic against hepatocellular carcinoma (HepG2), colorectal carcinoma (HCT-116) and breast adenocarcinoma cells (MCF-7) activity	*Hyrtios erectus*	The Red Sea, Egypt	12	Known
		Anti-H. pylori-antitubercular-cytotoxic activity	*Hyrtios erectus*	The Red Sea, Egypt	9	
19 Acetylsesterstatin 3	Sesterterpene	Anti-H. pylori-antitubercular	*Hyrtios erectus*	The Red Sea, Egypt	9	Known
12*β*,20*α*-Dihydroxy-16*β*-acetoxy-17-scalaren-19,20-olide	Sesterterpene	Anti-H. pylori-antitubercular-cytotoxic activities	*Hyrtios erectus*	The Red Sea, Egypt	9	Known
12-*O*-Acetyl-16-*O*-methylhyrtiolide	Sesterterpene	Anti-H. pylori-antitubercular-cytotoxic activities	*Hyrtios erectus*	The Red Sea, Egypt	9	Known

12*β*-Acetoxy,16*β*-methoxy,20*α*-hydroxy-17-scalaren-19,20-olide	Sesterterpene	Anti-H. pylori-antitubercular-cytotoxic activities	*Hyrtios erectus*	The Red Sea, Egypt	9	Known
12-*O*-Deacetyl-12,19-di-*epi*-scalarin	Sesterterpene	Anti-H. pylori & antitubercular activities	*Hyrtios erectus*	The Red Sea, Egypt	9	New
Smenotronic acid	Sesquiterpene	Antimalarial activity	*Hyrtios erectus*	Chuuk Island, Federated States of Micronesia	19	Known
Erectusolide B	Sesterterpene	NA	*Hyrtios erectus*	Similan Island, Thailand	7	New
Erectusolide C	Sesterterpene	NA	*Hyrtios erectus*	Similan Island, Thailand	7	New
Erectusolide D	Sesterterpene	Cytotoxic activity	*Hyrtios erectus*	Similan Island, Thailand	7	New
Seco-manoalide-25-methyl ether	Sesterterpene	NA	*Hyrtios erectus*	Similan Island, Thailand	7	New
Erectusfuranone A	Miscellaneous compound	Cytotoxic activity	*Hyrtios erectus*	Similan Island, Thailand	7	New
Erectusfuranone B	Miscellaneous compound	NA	*Hyrtios erectus*	Similan Island, Thailand	7	New
(6Z)-neomanoalide-24-acetate	Sesterterpene	Cytotoxic activity	*Hyrtios erectus*	Similan Island, Thailand	7	Known
24-O-Methylmanoalide	Sesterterpene	Cytotoxic activity	*Hyrtios erectus*	Similan Island, Thailand	7	Known
Luffariolide B	Sesterterpene	Cytotoxic activity	*Hyrtios erectus*	Similan Island, Thailand	7	Known
Manoalide	Sesterterpene	Cytotoxic activity	*Hyrtios erectus*	Similan Island, Thailand	7	Known
(6E)- and (6Z)-neomanoalide	Sesterterpene	Cytotoxic activity	*Hyrtios erectus*	Similan Island, Thailand	7	Known

Seco-manoalide	Sesterterpene	Cytotoxic activity	*Hyrtios erectus*	Similan Island, Thailand	7	Known
Cavernosine	Sesterterpene	Cytotoxic activity - Antimicrobial activity	*Hyrtios erectus*	Similan Island, Thailand	7	Known
			Fasciospongia cavernosa		20	
12-Acetylscalarolide	Sesterterpene	Cytotoxic activity	*Hyrtios erectus*	Similan Island, Thailand	7	Known
12-epi-O-deacetyl-19- deoxyscalarin	Sesterterpene	Cytotoxic activity	*Hyrtios erectus*	Similan Island, Thailand	7	Known
12-epi-scalarin	Sesterterpene	Cytotoxic activity	*Hyrtios erectus*	Similan Island, Thailand	7	Known
12-O-deacetyl-12-epi-scalarin	Sesterterpene	Cytotoxic activity	*Hyrtios erectus*	Similan Island, Thailand	7	Known
5-hydroxy-1H-indole-3-carbaldehyde	Nitrogenous compound	NA	*Hyrtios erectus*	Similan Island, Thailand	7	Known
Hyrtiosine A	Nitrogenous compound	NA	*Hyrtios erectus*	Similan Island, Thailand	7	Known
Variabine B	Nitrogenous compound	Cytotoxic activity	*Hyrtios erectus*	Similan Island, Thailand	7	Known
		Inhibition of chymotrypsin-like activity of the proteasome and Ubc13 (E2)–Uev1A interaction	*Luffariella variabilis*	North Sulawesi, Indonesia	21	
4’-Methylheptyl benzoate	Miscellaneous compound	Cytotoxic activity	*Hyrtios erectus*	Red Sea coast, Jeddah, Saudi Arabia	22	New

3-β-Hydroxycholest-5-en-7-one	Oxysterol	Cytotoxic activity	*Hyrtios erectus*	Red Sea coast, Jeddah, Saudi Arabia	22	Known
1H-indole-3-carbaldehyde	Nitrogenous compound	NA	*Hyrtios erectus*	Red Sea coast, Jeddah, Saudi Arabia	22	Known
Tryptoline	Nitrogenous compound	NA	*Hyrtios erectus*	Red Sea coast, Jeddah, Saudi Arabia	22	Known
6-Hydroxy-1,2,3,4-tetrahydro-9H-β-carboline-3-carboxylic acid	Nitrogenous compound	NA	*Hyrtios erectus*	Red Sea coast, Jeddah, Saudi Arabia	22	Known
Hyrtioreticulin B	Nitrogenous compound	NA	*Hyrtios erectus*	Red Sea coast, Jeddah, Saudi Arabia	22	Known
19-Methoxy-9,15-ene-puupehenol	Merosesquiterpene	Atherosclerosis inhibitor	*Hyrtios digitatus*	Australia	23	New
20-Methoxy-9,15-ene-puupehenol	Merosesquiterpene	Atherosclerosis inhibitor	*Hyrtios digitatus*	Australia	23	Known
Petrosaspongiolactam A	Sesterterpene	Potent inhibitors of TDP-43 protein	*Petrosaspongia species*	Fiji Islands	6	New
Petrosaspongiolactam B	Sesterterpene	Potent inhibitors of TDP-43 protein	*Petrosaspongia species*	Fiji Islands	6	New
Petrosaspongiolactam C	Sesterterpene	NA	*Petrosaspongia species*	Fiji Islands	6	New
Scalarolbutenolide	Sesterterpene	Potent inhibitors of TDP-43 protein	*Petrosaspongia species*	Fiji Islands	6	Known
Episcalarolbutenolide	Sesterterpene	Potent inhibitors of TDP-43 protein	*Petrosaspongia species*	Fiji Islands	6	Known
Smenamide A	Hybrid peptide/polyketide	Cytotoxic activity	*Smenospongia aurea*	Bahamas Islands	24	New
		NA	*Smenospongia conulosa*		25	
Smenamide B	Hybrid peptide/polyketide	Cytotoxic activity	*Smenospongia aurea*	Bahamas Islands	24	New
		NA	*Smenospongia conulosa*		25	

(E)-10-Benzyl-5,7-dimethylundeca-1,5,10-trien-4-ol	Miscellaneous compound	Cytotoxic activity	*Mixture of three sponges, Smenospongia aurea, S. cerebriformis, and Verongula rigida*	Florida	26	New
Smenothiazole A	Hybrid peptide/polyketide	Cytotoxic activity	*Smenospongia aurea*	Bahamas Islands	27	New
		NA	*Smenospongia conulosa*		25	
Smenothiazole B	Hybrid peptide/polyketide	Cytotoxic activity	*Smenospongia aurea*	Bahamas Islands	27	New
		NA	*Smenospongia conulosa*		25	
(−)-Nakijinol E	Sesquiterpene	NA	*Smenospongia aurea and S.cerebriformis*	Key Largo, Florida	28	New
(+)-5-epi-Nakijinol E	Sesquiterpene	Cytotoxic activity	*Smenospongia aurea and S.cerebriformis*	Key Largo, Florida	28	New
Mixture of nakijinone A and 5-epi-nakijinone A	Sesquiterpene	Cytotoxic activity	*Smenospongia aurea and S.cerebriformis*	Key Largo, Florida	28	New
(−)-Dactylospongenone E	Sesquiterpene	NA	*Smenospongia aurea and S.cerebriformis*	Key Largo, Florida	28	New
Mixture of 5-epi-dactylospongenones E and F	Sesquiterpene	NA	*Smenospongia aurea and S.cerebriformis*	Key Largo, Florida	28	New
(+)-5-epi-20-O-Ethylsmenoquinone	Sesquiterpene	Cytotoxic activity	*Smenospongia aurea and S.cerebriformis*	Key Largo, Florida	28	New
Smenopyrone	Miscellaneous compound	NA	*Smenospongia aurea*	The coast of Great Inagua (Bahamas Islands)	29	New
Smenolactone A	Polyketide	Antiproliferative activity	*Smenospongia aurea.*	Mayaguana Island, Bahamas	30	New
Smenolactone B	Polyketide	Antiproliferative activity	*Smenospongia aurea.*	Mayaguana Island, Bahamas	30	New
Smenolactone C	Polyketide	Antiproliferative activity	*Smenospongia aurea.*	Mayaguana Island, Bahamas	30	New
Smenolactone D	Polyketide	Antiproliferative activity	*Smenospongia aurea.*	Mayaguana Island, Bahamas	30	New

Conulothiazoles A	Hybrid peptide/polyketide	NA	*Smenospongia conulosa*	Bahamas Islands	[25]	New
Conulothiazoles B	Hybrid peptide/polyketide	NA	*Smenospongia conulosa*	Bahamas Islands	[25]	New
Smenocerone A	Miscellaneous compound	NA	*Smenospongia cerebriformis*	Eastern Sea of Quangtri, Vietnam	[31]	New
Smenocerone B	Miscellaneous compound	Cytotoxic activity towards lung carcinoma (LU-1), hepatocellular carcinoma (HepG-2), promyelocytic leukemia (HL-60), breast carcinoma (MCF-7), and melanoma (SK-Mel-2) human cancer cells	*Smenospongia cerebriformis*	Eastern Sea of Quangtri, Vietnam	[31]	New
Dactylospongenone A	Sesquiterpene	NA	*Smenospongia cerebriformis*	Eastern Sea of Quangtri, Vietnam	[31]	Known
Dactylospongenone B	Sesquiterpene	NA	*Smenospongia cerebriformis*	Eastern Sea of Quangtri, Vietnam	[31]	Known
Dactylospongenone C	Sesquiterpene	NA	*Smenospongia cerebriformis*	Eastern Sea of Quangtri, Vietnam	[31]	Known
Dactylospongenone D	Sesquiterpene	NA	*Smenospongia cerebriformis*	Eastern Sea of Quangtri, Vietnam	[31]	Known
Smenohaimien A	Sesquiterpene	Anti-inflammatory activity	*Smenospongia cerebriformis*	Vinhmoc, Quangtri	[32]	New
Smenohaimien B	Sesquiterpene	Anti-inflammatory activity	*Smenospongia cerebriformis*	Vinhmoc, Quangtri	[32]	New

Smenohaimien C	Sesquiterpene	Anti-inflammatory activity	*Smenospongia cerebriformis*	Vinhmoc, Quangtri	[32]	New
Smenohaimien D	Sesquiterpene	Anti-inflammatory activity	*Smenospongia cerebriformis*	Vinhmoc, Quangtri	[32]	New
Smenohaimien E	Sesquiterpene	Anti-inflammatory activity	*Smenospongia cerebriformis*	Vinhmoc, Quangtri	[32]	New
19-Hydroxy-polyfibrospongol B	Sesquiterpene	Anti-inflammatory activity	*Smenospongia cerebriformis*	Vinhmoc, Quangtri	[32]	Known
Polyfibrospongol B	Sesquiterpene	Anti-inflammatory activity	*Smenospongia cerebriformis*	Vinhmoc, Quangtri	[32]	Known
Smenohaimien F	Sesquiterpene	Cytotoxic activity	*Smenospongia cerebriformis*	Quang Tri, Vietnam	[33]	New
Neodactyloquinone	Sesquiterpene	NA	*Smenospongia cerebriformis*	Quang Tri, Vietnam	[33]	Known
Dactyloquinone C	Sesquiterpene	Cytotoxic activity (moderate)	*Smenospongia cerebriformis*	Quang Tri, Vietnam	[33]	Known
Dactyloquinone D	Sesquiterpene	Cytotoxic activity (moderate)	*Smenospongia cerebriformis*	Quang Tri, Vietnam	[33]	Known
Isoamijiol	Sesquiterpene	NA	*Smenospongia cerebriformis*	Quang Tri, Vietnam	[33]	Known
Amijiol	Sesquiterpene	NA	*Smenospongia cerebriformis*	Quang Tri, Vietnam	[33]	Known
Fasciospyrinadine	Sesquiterpene	NA	*Fasciospongia sp.*	Weizhou Island, Guangxi Autonomous Region, China.	[34]	New
Secomanoalide	Sesterterpene	Antimicrobial activity	*Fasciospongia cavernosa*	Not detected	[20]	known
4E,6E-dehydromanoalide	Sesterterpene	Antimicrobial activity	*Fasciospongia cavernosa*	Not detected	[20]	known
5-Hydroxyindole-3-glyoxylate methyl ester	Nitrogenous compound	Cytotoxic activity	*Scalarispongia sp.*	Dokdo island, Republic of Korea	[35]	New
Hyrtinadine B	Nitrogenous compound	Cytotoxic activity	*Scalarispongia sp.*	Dokdo island, Republic of Korea	[35]	New
Scalaridine A	Nitrogenous compound	Cytotoxic activity	*Scalarispongia sp.*	Dokdo island, Republic of Korea	[35]	New

Hyrtiosin A	Nitrogenous compound	Cytotoxic activity	*Scalarispongia sp.*	Dokdo island, Republic of Korea	35	Known
5-Hydroxytryprophol	Nitrogenous compound	Cytotoxic activity	*Scalarispongia sp.*	Dokdo island, Republic of Korea	35	Known
5-Hydroxy-1H-indole-3-carboxylic acid methyl ester	Nitrogenous compound	Cytotoxic activity	*Scalarispongia sp.*	Dokdo island, Republic of Korea	35	Known
5-Hydroxyindole-3- carbaldehyde	Nitrogenous compound	Cytotoxic activity	*Scalarispongia sp.*	Dokdo island, Republic of Korea	35	Known
Hyrtiosin B	Nitrogenous compound	Cytotoxic activity	*Scalarispongia sp.*	Dokdo island, Republic of Korea	35	Known
Hyrtinadine A	Nitrogenous compound	Cytotoxic activity	*Scalarispongia sp.*	Dokdo island, Republic of Korea	35	Known
Begonanline	Nitrogenous compound	Cytotoxic activity	*Scalarispongia sp.*	Dokdo island, Republic of Korea	35	Known
Scalarin	Sesterterpene	Cytotoxic activity	*Scalarispongia sp.*	Dokdo island, Republic of Korea	36	Known
12-*epi*-12-O-acetylscalarolide	Sesterterpene	Cytotoxic activity	*Scalarispongia sp.*	Dokdo island, Republic of Korea	36	Known
12-*O*-Acetyl-12,16-epi-scalarolbutenolide	Sesterterpene	Cytotoxic activity	*Scalarispongia sp.*	Dokdo island, Republic of Korea	36	Known
12*α*- Acetoxy-16β-hydroxyscalarolbutenolide	Sesterterpene	Cytotoxic activity	*Scalarispongia sp.*	Dokdo island, Republic of Korea	36	Known

16-*O*-Deacetyl-12,16-epi-scalarolbutanolide	Sesterterpene	NA	*Scalarispongia sp.*	Dokdo island, Republic of Korea	[36]	New
(4*S*,5b*R*,11a*S*,13a*S*,13b*R*)-5b,8,8,11a,13a-pentamethyl-2-oxo-2,4,5,5a,5b,6,7,7a,8,9,10,11,11a,11b,12,13,13a,13b-octadecahydrochryseno[1,2-b]furan-4-yl acetate	Sesterterpene	Cytotoxic activity	*Scalarispongia sp.*	Dokdo island, Republic of Korea	[36]	New
(4S,5b*R*,11a*S*,13a*S*,13b*R*)-4-hydroxy-5b,8,8,11a,13a-pentamethyl-5,5a,5b,6,7,7a,8,9,10,11,11a,11b,12,13,13a,13b-hexadecahydrochryseno[1,2-b]furan-2(*4H*)-one	Sesterterpene	Cytotoxic activity	*Scalarispongia sp.*	Dokdo island, Republic of Korea	[36]	New
(4a*S*,6*S*,6a*R*,10b*R*)-8-(hydroxymethyl)-1,1,4a,6a,10b-pentamethyl-9-oxo-1,2,3,4,4a,4b,5,6,6a,9,10,10a,10b,11,12,12a-hexadecahydrochrysen-6-yl acetate	Sesterterpene	Cytotoxic activity	*Scalarispongia sp.*	Dokdo island, Republic of Korea	[36]	New
Isoscalarafuran A	Sesterterpene	NA	*Scalarispongia sp.*	Dokdo (island), Republic of Korea	[37]	Known
Furoscalarol	Sesterterpene	NA	*Scalarispongia sp.*	Dokdo (island), Republic of Korea	[37]	Known
(5b*R*,11a*S*,13*S*,13a*R*)-5b,8,8,11a,13a-pentamethyl-4-oxo-4,5,5a,5b,6,7,7a,8,9,10,11,11a,11b,12,13,13a-hexadecahydrochryseno[1,2-b]furan-13-yl acetate	Sesterterpene	Cytotoxic activity	*Scalarispongia sp.*	Dokdo (island), Republic of Korea	[37]	Known
(5b*R*,11a*S*,13*S*,13a*S*)-5b,8,8,11a,13a-pentamethyl-4-oxo-4,5,5a,5b,6,7,7a,8,9,10,11,11a,11b,12,13,13a-hexadecahydro-1H-phenanthro[1,2-g]indol-13-yl acetate	Sesterterpene	Cytotoxic activity	*Scalarispongia sp.*	Dokdo (island), Republic of Korea	[37]	New
Scalaradial	Sesterterpene	Inhibitor of Transient Receptor Potential Melastatin 2 (TRPM2) Ion Channels	*Cacospongia sp.*	Pupukea, Beach Park, Oahu, Hawaii	[38]	Known

12-Deacetylscalaradial	Sesterterpene	Inhibitor of Transient Receptor Potential Melastatin 2 (TRPM2) Ion Channels	*Cacospongia sp.*	Pupukea, Beach Park, Oahu, Hawaii	38	Known
Cacolide A	Sesterterpene	NA	*Cacospongia sp.*	Australia	39	New
Cacolide B	Sesterterpene	NA	*Cacospongia sp.*	Australia	39	New
Cacolide C	Sesterterpene	NA	*Cacospongia sp.*	Australia	39	New
Cacolide D	Sesterterpene	NA	*Cacospongia sp.*	Australia	39	New
Cacolide E	Sesterterpene	NA	*Cacospongia sp.*	Australia	39	New
Cacolide F	Sesterterpene	NA	*Cacospongia sp.*	Australia	39	New
Cacolide G	Sesterterpene	NA	*Cacospongia sp.*	Australia	39	New
Cacolide H	Sesterterpene	NA	*Cacospongia sp.*	Australia	39	New
Cacolide I	Sesterterpene	NA	*Cacospongia sp.*	Australia	39	New
Cacolide J	Sesterterpene	NA	*Cacospongia sp.*	Australia	39	New
Cacolide K	Sesterterpene	NA	*Cacospongia sp.*	Australia	39	New
Cacolide L	Sesterterpene	NA	*Cacospongia sp.*	Australia	39	New
Cacolic acid A	Sesterterpene	NA	*Cacospongia sp.*	Australia	39	New
Cacolic acid B	Sesterterpene	NA	*Cacospongia sp.*	Australia	39	New
Cacolic acid C	Sesterterpene	NA	*Cacospongia sp.*	Australia	39	New
(+)-Cavernosine	Norditerpene	NA	*Cacospongia sp.*	Yong Xing Island	40	Known
(-)-Cavernosine	Norditerpene	NA	*Cacospongia sp.*	Yong Xing Island	40	Known
(+)-8,13-Secocavernosine	Norditerpene	NA	*Cacospongia sp.*	Yong Xing Island	40	Known
(-)-8,13-Secocavernosine	Norditerpene	NA	*Cacospongia sp.*	Yong Xing Island	40	Known

(+)-8,13-Secoepicavernosine	Norditerpene	NA	*Cacospongia sp.*	Yong Xing Island	[40]	New
(-)-8,13-Secoepicavernosine	Norditerpene	NA	*Cacospongia sp.*	Yong Xing Island	[40]	New
(+)-Hippolide E	Sesterterpene	NA	*Cacospongia sp.*	Yong Xing Island	[40]	New
(-)-Hippolide E	Sesterterpene	NA	*Cacospongia sp.*	Yong Xing Island	[40]	New
(+)-(6*E*)-neomanoalide	Sesterterpene	NA	*Cacospongia sp.*	Yong Xing Island	[40]	New
(-)-(6*E*)-neomanoalide	Sesterterpene	NA	*Cacospongia sp.*	Yong Xing Island	[40]	New
(*3R,4R*)-14,18-Secoluffariolide C	Sesterterpene	NA	*Cacospongia sp.*	Yong Xing Island	[40]	New
Cacospongine A	C21 pyridine terpenoid	NA	*Cacospongia sp.*	Yong Xing Island	[40]	New
Luffariolide C	Sesterterpene	NA	*Cacospongia sp.*	Yong Xing Island	[40]	known
(Z)-neomanoalide	Sesterterpene	NA	*Cacospongia sp.*	Yong Xing Island	[40]	known
Hippolide J	Sesterterpene	NA	*Cacospongia sp.*	Yong Xing Island	[40]	known
(−)-Zampanolide	Macrolide	Cytotoxic activity	*Cacospongia mycofijiensis*	Cathedral Cave, Tonga	[41]	Known
Zampanolide B	Macrolide	Cytotoxic activity	*Cacospongia mycofijiensis*	Cathedral Cave, Tonga	[41]	New
Zampanolide C	Macrolide	Cytotoxic activity	*Cacospongia mycofijiensis*	Cathedral Cave, Tonga	[41]	New
Zampanolide D	Macrolide	Cytotoxic activity	*Cacospongia mycofijiensis*	Cathedral Cave, Tonga	[41]	New
Zampanolides E	Macrolide	Cytotoxic activity	*Cacospongia mycofijiensis*	Cathedral Cave, Tonga	[41]	New
(−)-Dactylolide	Macrolide	Cytotoxic activity	*Cacospongia mycofijiensis*	Cathedral Cave, Tonga	[41]	Known

Thiopyrone CTP-431	Nitrogenous compound	NA	*Cacospongia mycofijiensis*	Fiji islands	42	New
Phyllospongin A	Sesterterpene	Cytotoxic activity &Antibacterial activity	*Phyllospongia lamellosa*	Shaab Saad area, Hurghada along the Red Sea Coast	43	New
Phyllospongin B	Sesterterpene	Cytotoxic activity &Antibacterial activity	*Phyllospongia lamellosa*	Shaab Saad area, Hurghada along the Red Sea Coast	43	New
Phyllospongin C	Sesterterpene	Cytotoxic activity &Antibacterial activity	*Phyllospongia lamellosa*	Shaab Saad area, Hurghada along the Red Sea Coast	43	New
Phyllospongin D	Sesterterpene	Cytotoxic activity &Antibacterial activity	*Phyllospongia lamellosa*	Shaab Saad area, Hurghada along the Red Sea Coast	43	New
Phyllospongin E	Sesterterpene	Cytotoxic activity &Antibacterial activity	*Phyllospongia lamellosa*	Shaab Saad area, Hurghada along the Red Sea Coast	43	New
12a-Acetoxy-20,24-dimethyl-25-norscalar-16-en-24-one	Sesterterpene	Cytotoxic activity &Antibacterial activity	*Phyllospongia lamellosa*	Shaab Saad area, Hurghada along the Red Sea Coast	43	Known
12a-Acetoxy-13b,18b-cyclobutan-20,24-dimethyl-24-oxoscalar-16-en-25b-ol	Sesterterpene	Cytotoxic activity &Antibacterial activity	*Phyllospongia lamellosa*	Shaab Saad area, Hurghada along the Red Sea Coast	43	Known
12a-Acetoxy-24,25-epoxy-24-hydroxy-20,24-dimethylscalarane	Sesterterpene	Cytotoxic activity &Antibacterial activity	*Phyllospongia lamellosa*	Shaab Saad area, Hurghada along the Red Sea Coast	43	Known

Scalardysin-A	Sesterterpene	Cytotoxic activity &Antibacterial activity	*Phyllospongia lamellosa*	Shaab Saad area, Hurghada along the Red Sea Coast	43	Known
2-(3′,5′-Dibromo-2′-methoxyphenoxy)-3,5-dibromophenol	Halogenated compound	Antibacterial activity	*Phyllospongia papyracea*	The natural products repository of the National Cancer Institute (NCI)	44	Known
Phyllactone A	Sesterterpene	Cytotoxic activity	*Phyllospongia papyrecea*	Sangihe Island, Indonesia	45	Known
Phyllactone B	Sesterterpene	Cytotoxic activity	*Phyllospongia papyrecea*	Sangihe Island, Indonesia	45	Known
Phyllactone D	Sesterterpene	Cytotoxic activity	*Phyllospongia papyrecea*	Sangihe Island, Indonesia	45	Known
Phyllactone E	Sesterterpene	Cytotoxic activity	*Phyllospongia papyrecea*	Sangihe Island, Indonesia	45	Known
Phyllactone F	Sesterterpene	Cytotoxic activity	*Phyllospongia papyrecea*	Sangihe Island, Indonesia	45	Known
Phyllactone G	Sesterterpene	Cytotoxic activity	*Phyllospongia papyrecea*	Sangihe Island, Indonesia	45	Known
12*α*, 24-Dihydroxy-20, 24-dimethyl-15, 17-scalaradien-25,24-olide	Sesterterpene	Cytotoxic activity	*Phyllospongia papyrecea*	Sangihe Island, Indonesia	45	Known
Phyllactone H	Sesterterpene	Cytotoxic activity	*Phyllospongia papyrecea*	Sangihe Island, Indonesia	45	New
12*α*-Acetoxy-22-hydroxy-24-methyl-24-oxoscalar-16-en-25-al	Sesterterpene	Antiproliferative activity	*Carteriospongia sp.*	The coast of Nosy Be, Madagascar	46	Known
16*β*,22-Dihydroxy-24-methyl-24-oxoscalaran-25,12β-olactone	Sesterterpene	NA	*Carteriospongia sp.*	The coast of Nosy Be, Madagascar	46	Known
12*α*,22-Dihydroxy-24-methyl-24-oxoscalar-16-en-25α-al	Sesterterpene	Antiproliferative activity	*Closely similar species to Carteriospongia sp.designated NB-04-06-17*	The coast of Nosy Be, Madagascar	46	New
12*β*,22-Dihydroxy-24-methyl-24-oxoscalar-16-en-25β-al	Sesterterpene	Antiproliferative activity	*Closely similar species to Carteriospongia sp.designated NB-04-06-17*	The coast of Nosy Be, Madagascar	46	New

12β,22-Dihydroxy-24-oxo-24-homoscalara-16,25(26)-diene	Sesterterpene	Antiproliferative activity	*Closely similar species to Carteriospongia sp.designated NB-04-06-17*	The coast of Nosy Be, Madagascar	46	New
12β,22,24ε-Trihydroxy-24-methylscalar-17-ene-18,24-carbolactone	Sesterterpene	Antiproliferative activity	*Closely similar species to Carteriospongia sp.designated NB-04-06-17*	The coast of Nosy Be, Madagascar	46	New
Carteriofenone A	Sesterterpene	Cytotoxic activity (Insignificant)	*Carteriospongia foliascens*	Dongluoxigu Island, the South China Sea	47	New
Carteriofenone B	Sesterterpene	Cytotoxic activity (Insignificant)	*Carteriospongia foliascens*	Dongluoxigu Island, the South China Sea	47	New
Carteriofenone C	Sesterterpene	Cytotoxic activity (Insignificant)	*Carteriospongia foliascens*	Dongluoxigu Island, the South China Sea	47	New
Carteriofenone D	Sesterterpene	Cytotoxic activity	*Carteriospongia foliascens*	Dongluoxigu Island, the South China Sea	47	New
Carteriofenone E	Sesterterpene	Cytotoxic activity	*Carteriospongia foliascens*	Dongluoxigu Island, the South China Sea	47	New
Carteriofenone F	Sesterterpene	Cytotoxic activity	*Carteriospongia foliascens*	Dongluoxigu Island, the South China Sea	47	New
Carteriofenone G	Sesterterpene	Cytotoxic activity	*Carteriospongia foliascens*	Dongluoxigu Island, the South China Sea	47	New

Carteriofenone H	Sesterterpene	Cytotoxic activity	*Carteriospongia foliascens*	Dongluoxigu Island, the South China Sea	47	New
Carteriofenone I	Sesterterpene	Cytotoxic activity	*Carteriospongia foliascens*	Dongluoxigu Island, the South China Sea	47	New
Carteriofenone J	Sesterterpene	Cytotoxic activity	*Carteriospongia foliascens*	Dongluoxigu Island, the South China Sea	47	New
Carteriofenone K	Sesterterpene	Cytotoxic activity	*Carteriospongia foliascens*	Dongluoxigu Island, the South China Sea	47	New
Phyllofenone B	Sesterterpene	Cytotoxic activity	*Carteriospongia foliascens*	Dongluoxigu Island, the South China Sea	47	known
Phyllofenone E	Sesterterpene	Cytotoxic activity	*Carteriospongia foliascens*	Dongluoxigu Island, the South China Sea	47	known
12*β*-(3'*β*-Hydroxybutanoyloxy)-20,24-dimethyl-24-oxo-scalara-16-en-25-al	Sesterterpene,	Antileukemic activity	*Carteriospongia sp.*	The coast of Tai-tung, Taiwan	48	New
12*β*-(3'*β*-Hydroxypentanoyloxy)-20,24-dimethyl-24-oxo-scalara-16-en-25-al	Sesterterpene,	Antileukemic activity	*Carteriospongia sp.*	The coast of Tai-tung, Taiwan	48	New
2-Tetraprenil-1,4-benzochinone	Meroditerpene	Antileukemic activity	*Carteriospongia sp.*	The coast of Tai-tung, Taiwan	48	Known

Puupehenol	Meroterpene	Antioxidant - Antimicrobial	*Dactylospongia sp.*	Hawaiian island of Maui	49	New
Puupehenone	Meroterpene	Antioxidant - Antimicrobial	*Dactylospongia sp.*	Hawaiian island of Maui	49	Known
Dactylospongin A	Sesquiterpene	Anti-inflammatory activity	*Dactylospongia sp*	Xisha Island of South China Sea	50	New
Dactylospongin B	Sesquiterpene	Anti-inflammatory activity	*Dactylospongia sp*	Xisha Island of South China Sea	50	New
Dactylospongin C	Sesquiterpene	NA	*Dactylospongia sp*	Xisha Island of South China Sea	50	New
Dactylospongin D	Sesquiterpene	Anti-inflammatory activity	*Dactylospongia sp*	Xisha Island of South China Sea	50	New
ent-Melemeleone B	Sesquiterpene	Anti-inflammatory activity	*Dactylospongia sp*	Xisha Island of South China Sea	50	known
Melemeleone C	Sesquiterpene	NA	*Dactylospongia sp*	Xisha Island of South China Sea	50	New

Melemeleone D	Sesquiterpene	NA	*Dactylospongia sp*	Xisha Island of South China Sea	50	New
Melemeleone E	Sesquiterpene	NA	*Dactylospongia sp*	Xisha Island of South China Sea	50	New
Dysidaminone N	Sesquiterpene	Anti-inflammatory activity	*Dactylospongia sp*	Xisha Island of South China Sea	50	New
19-*O*-Methylpelorol	Sesquiterpene	Cytotoxic activity - Anti-inflammatory activity	*Dactylospongia sp*	Xisha Island of South China Sea	50	New
Pelorol	Sesquiterpene	NA	*Dactylospongia sp*	Xisha Island of South China Sea	50	known
			Dactylospongia elegans	The island of Ambon, Indonesia	51	
		Antimalarial activity	*Hyrtios erectus*	Chuuk Island, Federated States of Micronesia	19	
17-O-Acetylavarol	Sesquiterpene	NA	*Dactylospongia sp*	Xisha Island of South China Sea	50	known
20-*O*-Acetylavarol	Sesquiterpene	NA	*Dactylospongia sp*	Xisha Island of South China Sea	50	known
20-*O*-Acetylneoavarol	Sesquiterpene	NA	*Dactylospongia sp*	Xisha Island of South China Sea	50	known

5-*epi*-nakijiquinone S	Sesquiterpene	Cytotoxic activity protein kinase inhibitory activity	*Dactylospongia metachromia*	Ambon islands, Indonesia	52	New
5-epinakijiquinone Q	Sesquiterpene	Cytotoxic activity protein kinase inhibitory activity	*Dactylospongia metachromia*	Ambon islands, Indonesia	52	New
		Antimicrobial activity	*Dactylospongia elegans*	Ambon islands, Indonesia	51	
5-epinakijiquinone T	Sesquiterpene	Cytotoxic activity protein kinase inhibitory activity	*Dactylospongia metachromia*	Ambon islands, Indonesia	52	New
5-epi-nakijiquinone U	Sesquiterpene	Cytotoxic activity protein kinase inhibitory activity	*Dactylospongia metachromia*	Ambon islands, Indonesia	52	New
5-epi-nakijiquinone N	Sesquiterpene	Cytotoxic activity protein kinase inhibitory activity	*Dactylospongia metachromia*	Ambon islands, Indonesia	52	New
5-epi-nakijinol C	Sesquiterpene	Protein kinase inhibitory activity	*Dactylospongia metachromia*	Ambon islands, Indonesia	52	New
5-epi-nakijinol D	Sesquiterpene	Protein kinase inhibitory activity	*Dactylospongia metachromia*	Ambon islands, Indonesia	52	New
18-Hydroxy-5-epihyrtiophenol	Sesquiterpene	Protein kinase inhibitory activity	*Dactylospongia metachromia*	Ambon islands, Indonesia	52	Known

1-*O*-13-Methyltetradecylglycerol	Miscellaneous compound	NA	*Dactylospongia metachromia*	Ambon islands, Indonesia	52	Known
Ilimaquinone	Sesquiterpene	Inhibits protein transport between successive Golgi cisternae by inducing the vesiculation of Golgi membranes - Antitumor (antiproliferative) at high concentration - antimicrobial activity	*Dactylospongia elegans*	The coast of Malaysia & Palau	53	Known
			Dactylospongia elegans	Sangihe Islands, North Sulawesi Province, Indonesia	54	
		Anti-inflammatory activity	*Smenospongia cerebriformis*	Vinhmoc, Quangtri	32	
		Antimalarial activity	*Hyrtios erectus*	Chuuk Island, Federated States of Micronesia	19	
5,8- Diepi-ilimaquinone	Sesquiterpene	HIF-1 activator - Antitumor	*Dactylospongia elegans*	The coast of Malaysia & Palau	53	New
4,5-Diepi-dactylospongiaquinone	Sesquiterpene	HIF-1 activator - Antitumor	*Dactylospongia elegans*	The coast of Malaysia & Palau	53	New

8-*epi*-Dactyloquinone B	Sesquiterpene	Antitumor	*Dactylospongia elegans*	The coast of Malaysia & Palau	[53]	New
10,17-*O*-Cyclo-4,5-diepi-dactylospongiaquinone	Sesquiterpene	Antitumor	*Dactylospongia elegans*	The coast of Malaysia & Palau	[53]	New
Cyclospongiacatechol	Sesquiterpene	Antitumor	*Dactylospongia elegans*	The coast of Malaysia & Palau	[53]	New
8-*epi*-ent-chromazonarol	Sesquiterpene	NA	*Dactylospongia elegans*	The coast of Malaysia & Palau	[53]	Known
Cyclospongiaquinone-1	Sesquiterpene	Antitumor	*Dactylospongia elegans*	The coast of Malaysia & Palau	[53]	Known
Cyclospongiaquinone-2	Sesquiterpene	Antitumor	*Dactylospongia elegans*	The coast of Malaysia & Palau	[53]	Known
19*R*-Dactyltronic acid	Sesquiterpene	Antibacterial activity	*Dactylospongia elegans*	Sanya, China	[55]	Known
19*S*-Dactyltronic acid	Sesquiterpene	Antibacterial activity	*Dactylospongia elegans*	Sanya, China	[55]	Known

Dictyoceratin C	Sesquiterpene	Antibacterial activity	*Dactylospongia elegans*	Sanya, China	[55]	Known
			Dactylospongia elegans	Sangihe Islands, North Sulawesi Province, Indonesia	[54]	
		Anti-inflammatory activity	*Smenospongia cerebriformis*	Vinhmoc, Quangtri	[32]	
Polyfibrospongol A	Sesquiterpene	Antibacterial activity	*Dactylospongia elegans*	Sanya, China	[55]	Known
		Anti-inflammatory activity	*Smenospongia cerebriformis*	Vinhmoc, Quangtri	[32]	
3 -Hydroxycholesta-5,8-dien-7-one	Sterol	Antibacterial activity	*Dactylospongia elegans*	Sanya, China	[55]	Known
Pregna-1,20-dien-3-one	Sterol	Antibacterial activity	*Dactylospongia elegans*	Sanya, China	[55]	Known
Eleganstone A	Diterpene	Weak antibacterial activity	*Dactylospongia elegans*	Yongxing Island, China	[56]	New
(1*R**,2*E*,4*R**,7*E*,10*S**,11*S**,12*R**)-10,18-Diacetoxydolabella-2,7-dien-6-one	Terpene	Weak antibacterial activity	*Dactylospongia elegans*	Yongxing Island, China	[56]	known
(1*R**,2*E*,4*R**,8*E*,10*S**,11*S**,12*R**)-10,18-diacetoxydolabella-2,8- dien-6-one	Diterpene	Weak antibacterial activity	*Dactylospongia elegans*	Yongxing Island, China	[56]	New
(1*R**,2*E*,4*R**,7*Z*,10*S**,11*S**,12*R**)-10,18-Diacetoxydolabella-2,7-dien-6-one	Diterpene	Weak antibacterial activity	*Dactylospongia elegans*	Yongxing Island, China	[56]	known
Dactylospongenone G	Drimane meroterpene	NA	*Dactylospongia elegans*	The island of Ambon, Indonesia	[51]	New
Dactylospongenone H	Drimane meroterpene	NA	*Dactylospongia elegans*	The island of Ambon, Indonesia	[51]	New
5-*epi*-ilimaquinone	Sesquiterpene	Cytotoxic activity	*Dactylospongia elegans*	The island of Ambon, Indonesia	[51]	known
5-*epi*-smenospongine	Sesquiterpene	NA	*Dactylospongia elegans*	The island of Ambon, Indonesia	[51]	known
5-*epi*-smenospongidine	Sesquiterpene	Cytotoxic activity	*Dactylospongia elegans*	The island of Ambon, Indonesia	[51]	known

Nakijiquinone D	Sesquiterpene	NA	*Dactylospongia elegans*	The island of Ambon, Indonesia	[51]	known
Smenospongine C	Sesquiterpene	NA	*Dactylospongia elegans*	The island of Ambon, Indonesia	[51]	known
Isospongiaquinone	Sesquiterpene	Cytotoxic activity	*Dactylospongia elegans*	The island of Ambon, Indonesia	[51]	known
Isosmenospongine	Sesquiterpene	Cytotoxic activity - Antimicrobial activity	*Dactylospongia elegans*	The island of Ambon, Indonesia	[51]	known
Nakijiquinone A	Sesquiterpene	Cytotoxic activity	*Dactylospongia elegans*	The island of Ambon, Indonesia	[51]	known
Nakijiquinone B	Sesquiterpene	NA	*Dactylospongia elegans*	The island of Ambon, Indonesia	[51]	known
Nakijiquinone C	Sesquiterpene	NA	*Dactylospongia elegans*	The island of Ambon, Indonesia	[51]	known
Nakijiquinone A	Sesquiterpene	Cytotoxic activity	*Dactylospongia elegans*	The island of Ambon, Indonesia	[51]	known
(3*S*,5*R*,9*R*,10*S*,13*R*,17*R*,20*R*,24*S*,22*E*)-ergosta-6,8,22-triene-3,25-diol	Sterol	Cytotoxic activity	*Dactylospongia elegans*	Xisha islands, China	[57]	New
(3*S*,5*R*,9*R*,10*S*,13*R*,17*R*,20*R*,24*S*,22*E*)-ergosta-6,8,22-triene-25-ol-3-sulfonate	Sterol	Cytotoxic activity	*Dactylospongia elegans*	Xisha islands, China	[57]	New
5α,8α-epidioxy-cholest-6-en-3β-ol	Sterol	NA	*Dactylospongia elegans*	Xisha islands, China	[57]	known
Popolohuanone G	Sesquiterpene	Anti-inflammatory activity	*Dactylospongia elegans*	Xisha islands, China	[58]	New
Popolohuanone H	Sesquiterpene	Anti-inflammatory activity	*Dactylospongia elegans*	Xisha islands, China	[58]	New

Popolohuanone I	Sesquiterpene	Anti-inflammatory activity	*Dactylospongia elegans*	Xisha islands, China	58	New
Popolohuanone B	Sesquiterpene	Anti-inflammatory activity	*Dactylospongia elegans*	Xisha islands, China	58	known
Popolohuanone C	Sesquiterpene	Anti-inflammatory activity	*Dactylospongia elegans*	Xisha islands, China	58	known
Nakijiquinone V	Merosesquiterpene	NA	*Dactylospongia elegans*	Sangihe Islands, North Sulawesi Province, Indonesia	54	New
Smenospongine	Merosesquiterpene	Antimicrobial activity	*Dactylospongia elegans*	Sangihe Islands, North Sulawesi Province, Indonesia	54	Known
20-demethoxy-20-isopentylaminodactyloquinone D	Sesquiterpene	NA	*Dactylospongia elegans*	Not detected	59	New
20-demethoxy-20-isobutylaminodactyloquinone D	Sesquiterpene	NA	*Dactylospongia elegans*	Not detected	59	New
19-methoxy-dictyoceratin-A	Sesquiterpene	Cytotoxic activity	*Dactylospongia elegans*	Not detected	59	New
(+)-Spiroreticulatine	Nitrogenous compound	Inhibitory activity on Interleukin-2 production but inactive against normal tumor cell lines	*Fascaplysinopsis reticulata*	The South China Sea	60	New

(−)-Spiroreticulatine	Nitrogenous compound	Inhibitory activity on Interleukin-2 production but inactive against normal tumor cell lines	*Fascaplysinopsis reticulata*	The South China Sea	60	New
Clavirolide H	Diterpene	Cytotoxic activity (Not significant)	*Fascaplysinopsis reticulata*	Xisha islands, China	61	New
Clavirolide D	Diterpene	NA	*Fascaplysinopsis reticulata*	Xisha islands, China	61	Known
Clavulactone	Diterpene	NA	*Fascaplysinopsis reticulata*	Xisha islands, China	61	Known
Cavernosine	C17 γ-lactone terpene	Cytotoxic activity	*Fascaplysinopsis reticulata*	Xisha islands, China	61	Known
Dihydrodysamide C	Nitrogenous compound	Cytotoxic activity	*Fascaplysinopsis reticulata*	Xisha islands, China	61	Known
(22*E*)-cholesta-4,6,8(14),22-tetraen-3-one	Sterol	Cytotoxic activity	*Fascaplysinopsis reticulata*	Xisha islands, China	61	Known
Cholesta-4,6,8(14)-trien-3-one	Sterol	NA	*Fascaplysinopsis reticulata*	Xisha islands, China	61	Known
(22*E*)-ergosta-4,6,8(14),22-tetraen-3-one	Sterol	NA	*Fascaplysinopsis reticulata*	Xisha islands, China	61	Known
(24*E*)- stigmasta-4,6,8(14),24(28)-tetraen-3-one	Sterol	Cytotoxic activity (Not significant)	*Fascaplysinopsis reticulata*	Xisha islands, China	61	Known
(24*S*)-ergosta-4,6,8(14)-trien-3-one	Sterol	Cytotoxic activity	*Fascaplysinopsis reticulata*	Xisha islands, China	61	Known
(22*E*)-stigmasta-4,6,8(14),22-tetraen-3-one	Sterol	Cytotoxic activity	*Fascaplysinopsis reticulata*	Xisha islands, China	61	Known
C(24)-epimeric mixture of stigmasta-4,6,8(14)-trien-3-one	Sterol	Cytotoxic activity	*Fascaplysinopsis reticulata*	Xisha islands, China	61	Known
6,60-bis-(debromo)-gelliusine F	Nitrogenous compound	Antimicrobial activity	*Fascaplysinopsis reticulata*	Mayotte	62	New
6-Bromo-8,1'-dihydro-isoplysin A	Nitrogenous compound	Antimicrobial activity	*Fascaplysinopsis reticulata*	Mayotte	62	New

5,6-Dibromo-8,1'-dihydro-isoplysin A	Nitrogenous compound	Antimicrobial activity	*Fascaplysinopsis reticulata*	Mayotte	62	New
8-Oxo-tryptamine	Nitrogenous compound	Moderate antimalarial activity	*Fascaplysinopsis reticulata*	Mayotte	62	Known
Tryptamine	Nitrogenous compound	Antimicrobial activity	*Fascaplysinopsis reticulata*	Mayotte	62	Known
(*E*)-6-Bromo-20-demethyl-30-N-methylaplysinopsin	Nitrogenous compound	Moderate antimalarial activity	*Fascaplysinopsis reticulata*	Mayotte	62	Known
(*Z*)-6-Bromo-20-demethyl-30-N-methylaplysinopsin	Nitrogenous compound	Moderate antimalarial activity	*Fascaplysinopsis reticulata*	Mayotte	62	Known
(+)& (−)Oxoaplysinopsin A	Nitrogenous compound	Cytotoxic activity & tyrosine phosphatase 1B (PTP1B) inhibition activity	*Fascaplysinopsis reticulata*	Xisha Island of South China Sea	63	New
(+)& (−)Oxoaplysinopsin B	Nitrogenous compound	Cytotoxic activity & tyrosine phosphatase 1B (PTP1B) inhibition activity	*Fascaplysinopsis reticulata*	Xisha Island of South China Sea	63	New
(+)& (−)Oxoaplysinopsin C	Nitrogenous compound	Cytotoxic activity & tyrosine phosphatase 1B (PTP1B) inhibition activity	*Fascaplysinopsis reticulata*	Xisha Island of South China Sea	63	New
(+)& (−)Oxoaplysinopsin D	Nitrogenous compound	Cytotoxic activity & tyrosine phosphatase 1B (PTP1B) inhibition activity	*Fascaplysinopsis reticulata*	Xisha Island of South China Sea	63	New

(+)& (−)Oxoaplysinopsin E	Nitrogenous compound	Cytotoxic activity & tyrosine phosphatase 1B (PTP1B) inhibition activity	*Fascaplysinopsis reticulata*	Xisha Island of South China Sea	63	New
(+)& (−)Oxoaplysinopsin F	Nitrogenous compound	Cytotoxic activity & tyrosine phosphatase 1B (PTP1B) inhibition activity	*Fascaplysinopsis reticulata*	Xisha Island of South China Sea	63	New
(+)& (−)Oxoaplysinopsin G	Nitrogenous compound	Cytotoxic activity & tyrosine phosphatase 1B (PTP1B) inhibition activity	*Fascaplysinopsis reticulata*	Xisha Island of South China Sea	63	New
(*Z*)-3′-Deimino-3′-oxoaplysinopsin	Nitrogenous compound	Cytotoxic activity & tyrosine phosphatase 1B (PTP1B) inhibition activity	*Fascaplysinopsis reticulata*	Xisha Island of South China Sea	63	Known
(*E*)-3′-Deimino-3′-oxoaplysinopsin	Nitrogenous compound	Cytotoxic activity & tyrosine phosphatase 1B (PTP1B) inhibition activity	*Fascaplysinopsis reticulata*	Xisha Island of South China Sea	63	Known
(*E*)-3-Indolylpropenoate	Nitrogenous compound	Cytotoxic activity & tyrosine phosphatase 1B (PTP1B) inhibition activity	*Fascaplysinopsis reticulata*	Xisha Island of South China Sea	63	Known

Indolyl-3-acetic acid methyl ester	Nitrogenous compound	Cytotoxic activity & tyrosine phosphatase 1B (PTP1B) inhibition activity	*Fascaplysinopsis reticulata*	Xisha Island of South China Sea	63	Known
3-Methoxycarbonylindole	Nitrogenous compound	Cytotoxic activity & tyrosine phosphatase 1B (PTP1B) inhibition activity	*Fascaplysinopsis reticulata*	Xisha Island of South China Sea	63	Known
3-Formylindole	Nitrogenous compound	Cytotoxic activity & tyrosine phosphatase 1B (PTP1B) inhibition activity	*Fascaplysinopsis reticulata*	Xisha Island of South China Sea	63	Known
			Hyrtios erectus	The Red Sea Coast, Jeddah	8	
3, 5-Dibromoverongiaquinol dimethyl ketal	Nitrogenous compound	Cytotoxic activity & tyrosine phosphatase 1B (PTP1B) inhibition activity	*Fascaplysinopsis reticulata*	Xisha Island of South China Sea	63	Known
Purealidin R	Nitrogenous compound	Cytotoxic activity & tyrosine phosphatase 1B (PTP1B) inhibition activity	*Fascaplysinopsis reticulata*	Xisha Island of South China Sea	63	Known
Aerothionin	Nitrogenous compound	Cytotoxic activity & tyrosine phosphatase 1B (PTP1B) inhibition activity	*Fascaplysinopsis reticulata*	Xisha Island of South China Sea	63	Known
Homoaerothionin	Nitrogenous compound	Cytotoxic activity & tyrosine phosphatase 1B (PTP1B) inhibition activity	*Fascaplysinopsis reticulata*	Xisha Island of South China Sea	63	Known
Subereamolline C	Nitrogenous compound	Cytotoxic activity & tyrosine phosphatase 1B (PTP1B) inhibition activity	*Fascaplysinopsis reticulata*	Xisha Island of South China Sea	63	New
Subereamolline D	Nitrogenous compound	Cytotoxic activity & tyrosine phosphatase 1B (PTP1B) inhibition activity	*Fascaplysinopsis reticulata*	Xisha Island of South China Sea	63	New

Luffalide A	Sesterterpene	NA	*Luffariella sp.*	The western coast of Pingtung county	64	New
Luffalide B	Sesterterpene	NA	*Luffariella sp.*	The western coast of Pingtung county	64	New
Luffalide C	Sesterterpene	NA	*Luffariella sp.*	The western coast of Pingtung county	64	New
Luffalide D	Sesterterpene	NA	*Luffariella sp.*	The western coast of Pingtung county	64	New
Luffalide E	Sesterterpene	Cytotoxic activity	*Luffariella sp.*	The western coast of Pingtung county	64	New
Luffalide F	Sesterterpene	NA	*Luffariella sp.*	The western coast of Pingtung county	64	New
Z-24-Acetoxyneomanoalide	Sesterterpene	NA	*Luffariella sp.*	The western coast of Pingtung county	64	Known

(*Z*)- neomanoalide 24,25-diacetate	Sesterterpene	Cytotoxic activity	*Luffariella sp.*	The western coast of Pingtung county	64	Known
Luffariellin B	Sesterterpene	Cytotoxic activity	*Luffariella sp.*	The western coast of Pingtung county	64	Known
25- Acetoxyluffariellin A	Sesterterpene	Cytotoxic activity	*Luffariella sp.*	The western coast of Pingtung county	64	Known
Cacofuran A	Diterpene	NA	*Luffariella sp.*	Cape Hedo, Okinawa	65	Known
Cacofuran B	Diterpene	NA	*Luffariella sp.*	Cape Hedo, Okinawa	65	Known
(3b*R*,7*S*,9a*S*)-3b,6,6,9a-tetramethyl-3b,4,5,5a,6,7,8,9,9a,9b,10,11-dodecahydrophenanthro[1,2-b]furan-7-ol	Diterpene	Cytotoxic activity	*Luffariella sp.*	Cape Hedo, Okinawa	65	New
(3b*R*,7*S*,9a*S*)-3b,6,6,9a-tetramethyl-3b,4,5,5a,6,7,8,9,9a,9b,10,11-dodecahydrophenanthro[1,2-b]furan-7-yl acetate	Diterpene	Cytotoxic activity	*Luffariella sp.*	Cape Hedo, Okinawa	65	New
Variabine A	Nitrogenous compound	Inhibition of chymotrypsin-like activity of the proteasome and Ubc13 (E2)–Uev1A interaction	*Luffariella variabilis*	North Sulawesi, Indonesia	21	New

5-((2*E*,6*E*)-7-((*Z*)-4-(3-(furan-3-yl)propylidene)tetrahydrofuran-2-yl)hepta-2,6-dien-1-yl)furan-3-carboxylic acid	Sesterterpene	Cytotoxic activity	*Luffariella variabilis*	Iriomote Island, Okinawa	66	New
6-(5-(2-(furan-3-yl)ethyl)-6-hydroxy-1,4a,6-trimethyldecahydronaphthalen-1-yl)-3-methyl-5,6-dihydro-2H-pyran-2-one	Sesterterpene	Cytotoxic activity	*Luffariella variabilis*	Iriomote Island, Okinawa	66	New
**B- Family: Dysideidae**						
5′-Monoacetylavarol	Sesquiterpene	NA	*Dysidea avara*	The Northwest Mediterranean sea	67	Known
(+)-Avarone	Sesquiterpene	Cytotoxic activity - NF-κB inhibiting activity - protein kinase inhibition - Insecticidal activity	*Dysidea avara*	Mediterranean Sea (Fethiye,Turkey)	68	known
(-)-3'-Methylaminoavarone	Sesquiterpene	Cytotoxic activity - NF-κB inhibiting activity - protein kinase inhibition - Insecticidal activity	*Dysidea avara*	Mediterranean Sea (Fethiye,Turkey)	68	known
(-)-4'-Methylaminoavarone	Sesquiterpene	Cytotoxic activity - NF-κB inhibiting activity	*Dysidea avara*	Mediterranean Sea (Fethiye,Turkey)	68	known

(-)-N-Methylmelemeleone-A	Sesquiterpene	Cytotoxic activity - protein kinase inhibition - Insecticidal activity	*Dysidea avara*	Mediterranean Sea (Fethiye,Turkey)	68	New
Dysideanone A	Sesquiterpene	NA	*Dysidea avara*	The south China sea	69	New
Dysideanone B	Sesquiterpene	Cytotoxic activity	*Dysidea avara*	The south China sea	69	New
Dysideanone C	Sesquiterpene	NA	*Dysidea avara*	The south China sea	69	New
Dysiquinol A	Sesquiterpene	Cytotoxic activity	*Dysidea avara*	The south China sea	70	New
Dysiquinol B	Sesquiterpene	Cytotoxic activity	*Dysidea avara*	The south China sea	70	New
Dysiquinol C	Sesquiterpene	Cytotoxic activity	*Dysidea avara*	The south China sea	70	New
Dysiquinol D	Sesquiterpene	Cytotoxic - NF-kB inhibitory activity	*Dysidea avara*	The south China sea	70	New
(5*S*,8*S*,9*R*,10*S*)-18-Ethoxyneoavarone	Sesquiterpene	Cytotoxic activity	*Dysidea avara*	The south China sea	70	New
(5*S*,8*S*,9*R*,10*S*)-19-Ethoxyneoavarone	Sesquiterpene	Cytotoxic activity	*Dysidea avara*	The south China sea	70	New

(5*R*,8*R*,9*S*,10*R*)-18-Ethoxyavarone	Sesquiterpene	Cytotoxic activity	*Dysidea avara*	The south China sea	70	New
(5*R*,8*R*,9*S*,10*R*)-19-Ethoxyavarone	Sesquiterpene	Cytotoxic activity	*Dysidea avara*	The south China sea	70	New
Avarol	Sesquiterpene	Cytotoxic activity	*Dysidea avara - .*	The south China sea	70	Known
		Cytotoxic activity - NF-κB inhibiting activity - protein kinase inhibition - Insecticidal activit	*Dysidea avara*	Mediterranean Sea (Fethiye,Turkey)	68	
			Dysidea avara		69	
		NA	*Dysidea avara*	The Northwest Mediterranean sea	67	
		Protein Tyrosine Phosphatase 1B Inhibitory Activity	*Dysidea sp*	Iriomote Island, Okinawa, Japan	71	
Biaketide	Miscellaneous compound	Cytotoxic activity	*Dysidea sp*	Biak, West Papua, Indonesia	72	New
Debromoantazirine	Nitrogenous compound (azirine) derivative	Cytotoxic activity	*Dysidea sp*	Biak, West Papua, Indonesia	72	New
Antazirine	Nitrogenous compound (azirine) derivative	NA	*Dysidea sp*	Biak, West Papua, Indonesia	72	Known
Dysidazirine	Nitrogenous compound (azirine) derivative	NA	*Dysidea sp*	Biak, West Papua, Indonesia	72	Known

neoavarol	Sesquiterpene	NA	*Dysidea sp*	Biak, West Papua, Indonesia	72	Known
		Protein Tyrosine Phosphatase 1B Inhibitory Activity	*Dysidea sp*	Iriomote Island, Okinawa, Japan.	71	
Frondosin	Miscellaneous compound	NA	*Dysidea sp*	Biak, West Papua, Indonesia	72	Known
Dysidinoid A	Meroterpene	Antibacterial activity	*Dysidea sp.*	Yongxing Island, Xiasha	73	New
2-(20,40-Dibromophenoxy)-4,6-dibromophenol	Halogenated compound	Antibacterial activity	*Dysidea sp.*	not detected	74	Known
Aureol B	Meroterpene	Cytotoxic and antimicrobial activities and weak inhibition against Na+/K+-ATPase	*Dysidea sp.*	Federated States of Micronesia	75	New
Melemeleone C	Meroterpene	Cytotoxic and antimicrobial activities and weak inhibition against Na+/K+-ATPase	*Dysidea sp.*	Federated States of Micronesia	75	New
Melemeleone D	Meroterpene	Cytotoxic and antimicrobial activities and weak inhibition against Na+/K+-ATPase	*Dysidea sp.*	Federated States of Micronesia	75	New
Cycloaurenone A	Meroterpene	Cytotoxic and antimicrobial activities and weak inhibition against Na+/K+-ATPase	*Dysidea sp.*	Federated States of Micronesia	75	New

Cycloaurenone B	Meroterpene	Cytotoxic and antimicrobial activities and weak inhibition against Na+/K+-ATPase	*Dysidea sp.*	Federated States of Micronesia	[75]	New
Cycloaurenone C	Meroterpene	Cytotoxic and antimicrobial activities and weak inhibition against Na+/K+-ATPase	*Dysidea sp.*	Federated States of Micronesia	[75]	New
Arenarol	Drimane sesquiterpene	Cytotoxic and antimicrobial activities and weak inhibition against Na+/K+-ATPase	*Dysidea sp.*	Federated States of Micronesia	[75]	Known
Dysidphenol A	Sesquiterpene	Antibacterial	*Dysidea species*	The south China sea	[76]	New
Dysidphenol B	Sesquiterpene	NA	*Dysidea species*	The south China sea	[76]	New
Dysidphenol C	Sesquiterpene	Antibacterial	*Dysidea species*	The south China sea	[76]	New
Smenospongimine	Sesquiterpene	Antibacterial	*Dysidea species*	The south China sea	[76]	New
Smenospongine	Sesquiterpene	Antibacterial	*Dysidea species*	The south China sea	[76]	Known
Smenospongorine	Sesquiterpene	Antibacterial	*Dysidea species*	The south China sea	[76]	Known
Smenospongiarine	Sesquiterpene	Antibacterial	*Dysidea species*	The south China sea	[76]	Known
Smenospongidine	Sesquiterpene	Antibacterial	*Dysidea species*	The south China sea	[76]	Known

Avapyran	Sesquiterpene	Protein Tyrosine Phosphatase 1B Inhibitory Activity	*Dysidea sp.*	Iriomote Island, Okinawa, Japan.	71	New
17-*O*-Acetylavarol	Sesquiterpene	Protein Tyrosine Phosphatase 1B Inhibitory Activity	*Dysidea sp.*	Iriomote Island, Okinawa, Japan.	71	New
17-*O*-Acetylneoavarol	Sesquiterpene	Protein Tyrosine Phosphatase 1B Inhibitory Activity	*Dysidea sp.*	Iriomote Island, Okinawa, Japan.	71	New
20-*O*-Acetylavarol	Sesquiterpene	Protein Tyrosine Phosphatase 1B Inhibitory Activity	*Dysidea sp.*	Iriomote Island, Okinawa, Japan.	71	Known
20-*O*-Acetylneoavarol	Sesquiterpene	Protein Tyrosine Phosphatase 1B Inhibitory Activity	*Dysidea sp.*	Iriomote Island, Okinawa, Japan.	71	Known
3’-Aminoavarone	Sesquiterpene	Protein Tyrosine Phosphatase 1B Inhibitory Activity	*Dysidea sp.*	Iriomote Island, Okinawa, Japan.	71	Known
Dysiroid A	Sterol	Antibacterial activity	*Dysidea sp.*	Dongshan Island, Guangdong Province, PR China	77	New
Dysiroid B	Sterol	Antibacterial activity	*Dysidea sp.*	Dongshan Island, Guangdong Province, PR China	77	New
(5*R*)-3,15,27-Triacontatriene-1,29-diyn-5-ol	Miscellaneous compound	Antibacterial activity	*Dysidea sp.*	Dongshan Island, Guangdong Province, PR China	77	Known
(4*R*,5*R*)-Muurol-1(6),10(14)-diene-4,5-diol	Sesquiterpene	NA	*Dysidea cinerea*	Lang Co beach, Thua Thien Hue, Vietnam	78	New

(4*R*,5*R*)-Muurol-1(6)-ene-4,5-diol	Sesquiterpene	NA	*Dysidea cinerea*	Lang Co beach, Thua Thien Hue, Vietnam	[78]	New
(4*R*,5*R*,10*R*)-10-Methoxymuurol-1(6)-ene-4,5-diol	Sesquiterpene	NA	*Dysidea cinerea*	Lang Co beach, Thua Thien Hue, Vietnam	[78]	New
(4*S*)-4-Hydroxy-1,10-seco-muurol-5-ene-1,10-dione	Sesquiterpene	NA	*Dysidea cinerea*	Lang Co beach, Thua Thien Hue, Vietnam	[78]	New
(4*R*)-4-Hydroxy-1,10-seco-muurol-5-ene-1,10-dione	Sesquiterpene	NA	*Dysidea cinerea*	Lang Co beach, Thua Thien Hue, Vietnam	[78]	New
(6*S*,10*S*)-6,10-Dihydroxy-7,8-seco-2,8-cyclo-muurol-4(5),7(11)-diene-12-oic acid	Sesquiterpene	NA	*Dysidea cinerea*	Lang Co beach, Thua Thien Hue, Vietnam	[78]	New
(6*R*,10*S*)-6,10-Dihydroxy-7,8-seco-2,8-cyclo-muurol-4(5),7(11)-diene-12-oic acid	Sesquiterpene	NA	*Dysidea cinerea*	Lang Co beach, Thua Thien Hue, Vietnam	[78]	New
Dysideolide A	Miscellaneous compound	NA	*Dysidea cinerea*	Lang Co beach, Vietnam	[79]	New
Dysideolide B	Miscellaneous compound	NA	*Dysidea cinerea*	Lang Co beach, Vietnam	[79]	New
Dysidaminone A	Sesquiterpene	Cytotoxic & NF-kB inhibitory activities	*Dysidea fragilis*	Yongxing Island, South China Sea	[80]	New
Dysidaminone B	Sesquiterpene	Cytotoxic & NF-kB inhibitory activities	*Dysidea fragilis*	Yongxing Island, South China Sea	[80]	New
Dysidaminone C	Sesquiterpene	Cytotoxic & NF-kB inhibitory activities	*Dysidea fragilis*	Yongxing Island, South China Sea	[80]	New
Dysidaminone D	Sesquiterpene	Cytotoxic & NF-kB inhibitory activities	*Dysidea fragilis*	Yongxing Island, South China Sea	[80]	New

Dysidaminone E	Sesquiterpene	Cytotoxic & NF-kB inhibitory activities	*Dysidea fragilis*	Yongxing Island, South China Sea	[80]	New
Dysidaminone F	Sesquiterpene	Cytotoxic & NF-kB inhibitory activities	*Dysidea fragilis*	Yongxing Island, South China Sea	[80]	New
Dysidaminone G	Sesquiterpene	Cytotoxic & NF-kB inhibitory activities	*Dysidea fragilis*	Yongxing Island, South China Sea	[80]	New
Dysidaminone H	Sesquiterpene	Cytotoxic & NF-kB inhibitory activities	*Dysidea fragilis*	Yongxing Island, South China Sea	[80]	New
Dysidaminone I	Sesquiterpene	Cytotoxic & NF-kB inhibitory activities	*Dysidea fragilis*	Yongxing Island, South China Sea	[80]	New
Dysidaminone J	Sesquiterpene	Cytotoxic & NF-kB inhibitory activities	*Dysidea fragilis*	Yongxing Island, South China Sea	[80]	New
Dysidaminone K	Sesquiterpene	Cytotoxic & NF-kB inhibitory activities	*Dysidea fragilis*	Yongxing Island, South China Sea	[80]	New
Dysidaminone L	Sesquiterpene	Cytotoxic & NF-kB inhibitory activities	*Dysidea fragilis*	Yongxing Island, South China Sea	[80]	New
Dysidaminone M	Sesquiterpene	Cytotoxic & NF-kB inhibitory activities	*Dysidea fragilis*	Yongxing Island, South China Sea	[80]	New
18-Methylaminoavarone	Sesquiterpene	Cytotoxic & NF-kB inhibitory activities	*Dysidea fragilis*	Yongxing Island, South China Sea	[80]	Known
19-Methylaminoavarone	Sesquiterpene	Cytotoxic & NF-kB inhibitory activities	*Dysidea fragilis*	Yongxing Island, South China Sea	[80]	Known
18-Aminoavarone	Sesquiterpene	Cytotoxic & NF-kB inhibitory activities	*Dysidea fragilis*	Yongxing Island, South China Sea	[80]	Known
19-Aminoavarone	Sesquiterpene	Cytotoxic & NF-kB inhibitory activities	*Dysidea fragilis*	Yongxing Island, South China Sea	[80]	Known
18-Phenethylaminoavarone	Sesquiterpene	Cytotoxic & NF-kB inhibitory activities	*Dysidea fragilis*	Yongxing Island, South China Sea	[80]	Known

Popolohuanone D	Sesquiterpene	Cytotoxic & NF-kB inhibitory activities	*Dysidea fragilis*	Yongxing Island, South China Sea	[80]	Known
Dysinidin A	Sesquiterpene	NA	*Dysidea fragilis*	Vandon, Quangninh	[81]	New
Dysinidin B	Sesquiterpene	NA	*Dysidea fragilis*	Vandon, Quangninh	[81]	New
Furodysinin lactone	Sesquiterpene	NA	*Dysidea fragilis*	Vandon, Quangninh	[81]	Known
O-Methyl furodysinin lactone	Sesquiterpene	NA	*Dysidea fragilis*	Vandon, Quangninh	[81]	Known
Dysinidin C	Sesquiterpene	NA	*Dysidea fragilis*	Vandon, Quangninh	[82]	New
Dysinidin D	Sesquiterpene	NA	*Dysidea fragilis*	Vandon, Quangninh	[82]	New
Dysinidin E	Sesquiterpene	NA	*Dysidea fragilis*	Vandon, Quangninh	[82]	New
Dysideasterol F	Sterol	Cytotoxic activity	*Dysidea fragilis*	Vandon, Quangninh	[82]	Known
9α,11α-Epoxycholest-7-en-3β,5α,6α-triol	Sterol	Cytotoxic activity	*Dysidea fragilis*	Vandon, Quangninh	[82]	Known
9α,11α-Epoxycholest-7-en-3β,5α,6α,19-tetrol 6-acetate	Sterol	Cytotoxic activity	*Dysidea fragilis*	Vandon, Quangninh	[82]	Known
Dysifragilone A	Sesquiterpene	Anti-inflammatory activity	*Dysidea fragilis*	Yongxing Island in the South China Sea	[83]	Known
Dysivillosin A	Meroterpene	Antiallergic- Down regulation of production of lipids	*Dysidea villosa*	The south China sea	[84]	New

Dysivillosin B	Meroterpene	Antiallergic- Down regulation of production of lipids	*Dysidea villosa*	The south China sea	84	New
Dysivillosin C	Meroterpene	Antiallergic- Down regulation of production of lipids	*Dysidea villosa*	The south China sea	84	New
Dysivillosin D	Meroterpene	Antiallergic- Down regulation of production of lipids	*Dysidea villosa*	The south China sea	84	New
2-(20,40-Dibromophenoxy)-3,5-dibromophenol	Halogenated compound	Antibacterial activity	*Dysidea granulosa*	Not detected	74	Known
2-(20,40-Dibromophenoxy)- 3,4,5-tribromophenol	Halogenated compound	Antibacterial activity	*Dysidea granulosa*	Not detected	74	Known
3,5-Dibromo-2-(2,4-dibromophenoxy)phenol	Halogenated compound	Antimicrobial	*Dysidea granulosa*	Papua New Guinea	85	known
		Cytotoxic activity	*Dysidea sp.*	Palau Islands-Maumere, Indonesia	86	
2,4-dibromo-6-(2,4-dibromophenoxy)phenol	Halogenated compound	Antimicrobial	*Dysidea granulosa*	Papua New Guinea - Palau Islands	85	known
3,4,5-Tribromo-2-(2,4-dibromophenoxy)phenol	Halogenated compound	Antimicrobial	*Dysidea granulosa*	Papua New Guinea - Palau Islands	85	known
2,3,4-Tribromo-6-(2,4-dibromophenoxy)phenol	Halogenated compound	Antimicrobial	*Dysidea granulosa*	Papua New Guinea - Palau Islands	85	known
2,3,4,5-Tetrabromo-6-(2,4-dibromophenoxy)phenol	Halogenated compound	Antimicrobial	*Dysidea granulosa*	Papua New Guinea - Palau Islands	85	known

1,5-Dibromo-3-(2,4-dibromophenoxy)-2-methylbenzene	Halogenated compound	NA	*Dysidea granulosa*	Papua New Guinea - Palau Islands	[85]	known
((3a*S*,3b*R*,5a*S*,9a*S*,9b*R*,10*S*)-10-Hydroxy-6,6,9a-trimethyl-1-oxo-1,3,3a,3b,4,5,5a,6,7,8,9,9a,9b,10-tetradecahydrophenanthro[1,2-c]furan-3b-yl)methyl 3-methylbutanoate	Diterpene	Cytotoxic activity	*Dysidea cf. arenaria*	Irabu Island, Okinawa	[87]	New
((3a*R*,3b*R*,5a*S*,9a*S*,9b*R*,10*S*,11a*R*)-10-Hydroxy-6,6,9a-trimethyl-1-oxohexadecahydrophenanthro[1,2-c]furan-3b-yl)methyl 3-methylbutanoate	Diterpene	Cytotoxic activity	*Dysidea cf. arenaria*	Irabu Island, Okinawa	[87]	New
((4a*S*,4b*R*,5*S*,8*S*,8a*S*,10a*S*)-7,8-Diformyl-5-hydroxy-1,1,4a-trimethyl-1,2,3,4,4a,4b,5,8,8a,9,10,10a-dodecahydrophenanthren-8a-yl)methyl 3-methylbutanoate	Diterpene	Cytotoxic activity	*Dysidea cf. arenaria*	Irabu Island, Okinawa	[87]	New
(1*R*,3*R*,4*S*,5*R*,5a*S*,7a*S*,11a*S*,11b*R*,12*R*)-5-Formyl-8,8,11a-trimethyl-5a-(((3-methylbutanoyl)oxy)methyl)tetradecahydro-1,4-methanonaphtho[1,2-c]oxepine-3,12-diyl diacetate	Diterpene	Cytotoxic activity	*Dysidea cf. arenaria*	Irabu Island, Okinawa	[87]	New
Septosone A	Meroterpene	Anti-inflammatory activity	*Dysidea septosa*	The south China sea	[88]	New
Septosone B	Meroterpene	NA	*Dysidea septosa*	The south China sea	[88]	New
Septosone C	Meroterpene	Anti-inflammatory activity (weak)	*Dysidea septosa*	The south China sea	[88]	New

2-(30,50-Dibromo-20-methoxyphenoxy)-3,5-dibromophenol	Halogenated compound	Inhibit Protein tyrosine phosphatase 1B activity - Moderate cytotoxic activity	*Lamellodysidea herbacea*	Manado, Indonesia	89	Known
Lamellodysidine A	Sesquiterpene	NA	*Lamellodysidea herbacea*	Manadotua Island, Indonesia	90	New
Lamellodysidine B	Sesquiterpene	NA	*Lamellodysidea herbacea*	Manadotua Island, Indonesia	90	New
O,Odimethyllingshuiolide A	Sesquiterpene	NA	*Lamellodysidea herbacea*	Manadotua Island, Indonesia	90	New
11-epi-O,O-dimethyllingshuiolide A	Sesquiterpene	NA	*Lamellodysidea herbacea*	Manadotua Island, Indonesia	90	New
3,5-Dibromobenzene-1,2-diol	Halogenated compound	NA	*Lamellodysidea sp.*	Papua New Guinea	85	Known
3,5-Dibromo-2-(3,5-dibromo-2-hydroxyphenoxy)phenol	Halogenated compound	Antimicrobial	*Lamellodysidea sp.*	Papua New Guinea	85	Known
3,4,6-Tribromo-2-(3,5-dibromo-2-hydroxyphenoxy)phenol	Halogenated compound	Antimicrobial	*Lamellodysidea sp.*	Papua New Guinea	85	Known
2,3,5-Tribromo-6-(3,5-dibromo-2-hydroxyphenoxy)phenol	Halogenated compound	Antimicrobial	*Lamellodysidea sp.*	Papua New Guinea	85	Known
2,3,4-Tribromo-6-(3,5-dibromo-2-hydroxyphenoxy)phenol	Halogenated compound	Antimicrobial	*Lamellodysidea sp.*	Papua New Guinea	85	Known

3,4,5-Tribromo-2-(3,5-dibromo-2-hydroxyphenoxy)phenol	Halogenated compound	Antimicrobial	*Lamellodysidea sp.*	Papua New Guinea	[85]	Known
		Cytotoxic activity	*Dysidea sp.*	Maumere, Indonesia	[86]	
2,3,4,5-Tetrabromo-6-(3,5-dibromo-2-hydroxyphenoxy)phenol	Halogenated compound	Antimicrobial	*Lamellodysidea sp.*	Papua New Guinea	[85]	Known
1-(3',5',6'-Tribromo-4'-methoxy-1'-hydroxyphenoxy)-3,5-dibromo-2-phenol	Halogenated compound	Antimicrobial	*Lamellodysidea sp.*	Papua New Guinea	[85]	New
**C- Family: Spongiidae**						
(+)-Sponalisolide A	Miscellaneous compound	Antibacterial against Pseudomonas aeruginosa	*Spongia officinalis*	The coast of Weizhou island, China	[91]	New
(−)-Sponalisolide A	Miscellaneous compound	Antibacterial against Pseudomonas aeruginosa	*Spongia officinalis*	The coast of Weizhou island, China	[91]	New
(+)-Sponalisolide B	Miscellaneous compound	Antibacterial against Pseudomonas aeruginosa	*Spongia officinalis*	The coast of Weizhou island, China	[91]	New
(−)-Sponalisolide B	Miscellaneous compound	Antibacterial against Pseudomonas aeruginosa	*Spongia officinalis*	The coast of Weizhou island, China	[91]	New
3-Nor-spongiolide A	Diterpene	NA	*Spongia officinalis*	South China Sea	[92]	New
Spongiolide A	Diterpene	NA	*Spongia officinalis*	South China Sea	[92]	New
Spongiolide B	Diterpene	NA	*Spongia officinalis*	South China Sea	[92]	New

Spongia-13(16),14-diene	Diterpene	NA	*Spongia officinalis*	South China Sea	92	Known
18-nor-3,17- Dihydroxyspongia-3,13(16),14-trien-2-one	Diterpene	NA	*Spongia officinalis*	South China Sea	92	Known
Spongiatriol	Diterpene	NA	*Spongia officinalis*	South China Sea	92	Known
		Antiviral activity & Cytotoxic activity	*Hyattella aff. Intestinalis*	Iriomote Island, Okinawa	93	
Isospongiatriol	Diterpene	NA	*Spongia officinalis*	South China Sea	92	Known
		Antiviral activity & Cytotoxic activity	*Hyattella aff. Intestinalis*	Iriomote Island, Okinawa	93	
Epispongiatriol	Diterpene	NA	*Spongia officinalis*	South China Sea	92	Known
		Antiviral activity & Cytotoxic activity	*Hyattella aff. Intestinalis*	Iriomote Island, Okinawa	93	
17,19-Dihydroxyspongia-13(16),14-dien-2,3-dione	Diterpene	NA	*Spongia officinalis*	South China Sea	92	Known
18-nor-3,17-dihydroxyspongia-3,13(16),14-trien-2-one	Diterpene	Inhibition of TNF-α-activated NF-κB activity - Inhibition of aromatase	*Spongia sp*	Sulawesi, Indonesia	94	New
18-nor-3,5,17-trihydroxyspongia-3,13(16),14-trien-2-one	Diterpene	Inhibition of TNF-α-activated NF-κB activity	*Spongia sp*	Sulawesi, Indonesia	94	New

Spongiapyridine	Diterpene	Inhibition of TNF-α-activated NF-κB activity	*Spongia sp*	Sulawesi, Indonesia	94	New
17-Hydroxy-4-epi-spongialactone A	Diterpene	Inhibition of TNF-α-activated NF-κB activity	*Spongia sp*	Sulawesi, Indonesia	94	Known
(1*R*,5b*R*,11a*S*,13a*S*,13b*R*)-1-Methoxy-5b,8,8,11a,13a-pentamethyl-1,5,5a,6,7,7a,8,9,10,11,11a,11b,12,13,13a,13b-hexadecahydrochryseno[1,2-c]furan-3(5bH)-one	Sesterterpene	NA	*Spongia sp*	Geoje Island, South Sea of Korea	95	New
(1*R*,4b*R*,10a*S*,12*R*,12a*S*)-Methyl 1-formyl-12-hydroxy-4b,7,7,10a,12a-pentamethyl-1,4,4a,4b,5,6,6a,7,8,9,10,10a,10b,11,12,12a-hexadecahydrochrysene-2-carboxylate	Sesterterpene	NA	*Spongia sp*	Geoje Island, South Sea of Korea	95	New
Furospongin-1	Sesterterpene	Protein tyrosine phosphatase (PTP) 1B inhibitor	*Spongia sp.*	Manado, Indonesia	96	New
Langcoquinone D	Sesquiterpene	Antibacterial & cytotoxic activity	*Spongia sp.*	Thua Thien-Hue city, Vietnam	97	New
Langcoquinone E	Sesquiterpene	NA	*Spongia sp.*	Thua Thien-Hue city, Vietnam	97	New
Langcoquinone F	Sesquiterpene	NA	*Spongia sp.*	Thua Thien-Hue city, Vietnam	97	New
Scalalactam A	Sesterterpene	NA	*Spongia sp.*	The South Sea near Tong-Yong City, Korea	98	New
Scalalactam B	Sesterterpene	NA	*Spongia sp.*	The South Sea near Tong-Yong City, Korea	98	New

Scalalactam C	Sesterterpene	NA	*Spongia sp.*	The South Sea near Tong-Yong City, Korea	[98]	New
Scalalactam D	Sesterterpene	NA	*Spongia sp.*	The South Sea near Tong-Yong City, Korea	[98]	New
18-Deoxy-18-formamidodictyoceratin B	Sesquiterpene	Cytotoxic activity	*Spongia pertusa Esper*	Yongxing Island	[99]	New
18-Deoxy-18-(2-hydroxyacetyl)aminodictyoceratin B	Sesquiterpene	Cytotoxic activity	*Spongia pertusa Esper*	Yongxing Island	[99]	New
Dictyoceratin D	Sesquiterpene	Cytotoxic activity	*Spongia pertusa Esper*	Yongxing Island	[99]	New
N-Methyl-ent-smenospongine	Sesquiterpene	Cytotoxic activity	*Spongia pertusa Esper*	Yongxing Island	[99]	New
N-Methyl-5-epi-smenospongine	Sesquiterpene	Cytotoxic activity	*Spongia pertusa Esper*	Yongxing Island	[99]	New
20-Demethoxy-20-methylaminodactyloquinone D	Sesquiterpene	Cytotoxic activity	*Spongia pertusa Esper*	Yongxing Island	[99]	New
20-Demethoxy-20-methylamino-5-epi-dactyloquinone D	Sesquiterpene	Cytotoxic activity	*Spongia pertusa Esper*	Yongxing Island	[99]	New
20-Demethoxy-20-methylaminodactyloquinone B	Sesquiterpene	Cytotoxic activity	*Spongia pertusa Esper*	Yongxing Island	[99]	New
Yahazunol B	Sesquiterpene	Cytotoxic activity	*Spongia pertusa Esper*	Yongxing Island	[99]	New
5-*epi*-smenospongine	Sesquiterpene	Cytotoxic activity	*Spongia pertusa Esper*	Yongxing Island	[99]	Known
Smenospongiadine	Sesquiterpene	Cytotoxic activity	*Spongia pertusa Esper*	Yongxing Island	[99]	Known
Ceylonamide A	Diterpene	Inhibition of RANKL induced osteoclastogenesis	*Spongia ceylonensis*	Indonesia	[100]	New
Ceylonamide B	Diterpene	Inhibition of RANKL induced osteoclastogenesis	*Spongia ceylonensis*	Indonesia	[100]	New
Ceylonamide C	Diterpene	Inhibition of RANKL induced osteoclastogenesis	*Spongia ceylonensis*	Indonesia	[100]	New
Ceylonamide D	Diterpene	Inhibition of RANKL induced osteoclastogenesis	*Spongia ceylonensis*	Indonesia	[100]	New
Ceylonamide E	Diterpene	Inhibition of RANKL induced osteoclastogenesis	*Spongia ceylonensis*	Indonesia	[100]	New

Ceylonamide F	Diterpene	Inhibition of RANKL induced osteoclastogenesis	*Spongia ceylonensis*	Indonesia	[100]	New
15α,16-dimethoxyspongi-13-en-19-oic acid	Diterpene	Inhibition of RANKL induced osteoclastogenesis	*Spongia ceylonensis*	Indonesia	[100]	New
Haumanamide	Diterpene	Inhibition of RANKL induced osteoclastogenesis	*Spongia ceylonensis*	Indonesia	[100]	Known
Spongia-13(16),14-dien-19-oic acid	Diterpene	Inhibition of RANKL induced osteoclastogenesis	*Spongia ceylonensis*	Indonesia	[100]	Known
Spongiadiol	Diterpene	Inhibition of RANKL induced osteoclastogenesis	*Spongia ceylonensis*	Indonesia	[100]	Known
Isospongiadiol	Diterpene	Inhibition of RANKL induced osteoclastogenesis	*Spongia ceylonensis*	Indonesia	[100]	Known
Ceylonin A	Diterpene	Inhibition of RANKL induced osteoclastogenesis	*Spongia ceylonensis*	Indonesia	[101]	New
Ceylonin B	Diterpene	Inhibition of RANKL induced osteoclastogenesis	*Spongia ceylonensis*	Indonesia	[101]	New
Ceylonin C	Diterpene	Inhibition of RANKL induced osteoclastogenesis	*Spongia ceylonensis*	Indonesia	[101]	New
Ceylonin D	Diterpene	Inhibition of RANKL induced osteoclastogenesis	*Spongia ceylonensis*	Indonesia	[101]	New
Ceylonin E	Diterpene	Inhibition of RANKL induced osteoclastogenesis	*Spongia ceylonensis*	Indonesia	[101]	New

Ceylonin F	Diterpene	Inhibition of RANKL induced osteoclastogenesis	*Spongia ceylonensis*	Indonesia	101	New
Spongia- 13(16),14-dien-19-oic acid	Diterpene	NA	*Spongia ceylonensis*	Indonesia	101	Known
Ceylonin G	Diterpene	NA	*Spongia ceylonensis*	Indonesia	102	New
Ceylonin H	Diterpene	NA	*Spongia ceylonensis*	Indonesia	102	New
Ceylonin I	Diterpene	NA	*Spongia ceylonensis*	Indonesia	102	New
ent-13-norisocopalen-15-al-18-oic acid	Diterpene	Ubiquitin-specific protease 7 (USP7) inhibitor (anticancer)	*Spongia ceylonensis*	Indonesia	102	Known
15-oxospongi-13-en-19-oic acid	Diterpene	NA	*Spongia ceylonensis*	Indonesia	102	Known
		NA	*Spongia ceylonensis*	Indonesia	100	
16-Oxospongi-13-en-19-oic acid	Diterpene	NA	*Spongia ceylonensis*	Indonesia	102	Known
			Spongia ceylonensis	Indonesia	100	
Spongiabutenolide A	Diterpene	NA	*Spongia ceylonensis*	Indonesia	102	Known
			Spongia ceylonensis	Indonesia	100	
Spongiabutenolide B	Diterpene	NA	*Spongia ceylonensis*	Indonesia	102	Known
			Spongia ceylonensis	Indonesia	100	
Epi-ilimaquinone	Sesquiterpene	Cytotoxic activity - Antimicrobial activity	*Hippospongia sp*	Fiji island, Melanesia	103	Known
Smenospongine	Sesquiterpene	Cytotoxic activity	*Hippospongia sp*	Fiji island, Melanesia	103	Known
			Spongia pertusa Esper	Yongxing Island	99	

Glycinylilimaquinone	Sesquiterpene	Cytotoxic activity	*Hippospongia sp*	Fiji island, Melanesia	103	Known
Hippospongide C	Sesterterpene	Cytotoxic activity	*Hippospongia sp.*	The coast of Tai-tung, Taiwan	104	New
12-Deacetyl-12-epi-scalaradial	Sesterterpene	Cytotoxic activity	*Hippospongia sp.*	The coast of Tai-tung, Taiwan	104	Known
12-*epi*-scalaradial	Sesterterpene	Cytotoxic activity	*Hippospongia sp.*	The coast of Tai-tung, Taiwan	104	Known
12-Deacetyl-12,18-diepi-scalaradial	Sesterterpene	Cytotoxic activity	*Hippospongia sp.*	The coast of Tai-tung, Taiwan	104	Known
12,18-Diepiscalaradial	Sesterterpene	Cytotoxic activity	*Hippospongia sp.*	The coast of Tai-tung, Taiwan	104	Known
Rhopaloic acid H	Sesterterpene	Cytotoxic activity	*Hippospongia sp.*	The coast of Pingtung county	105	New
Rhopaloic acid A	Sesterterpene	Cytotoxic activity	*Hippospongia sp.*	The coast of Pingtung county	105	Known
Rhopaloic acid C	Sesterterpene	Cytotoxic activity	*Hippospongia sp.*	The coast of Pingtung county	105	Known
Heteronemin	Sesterterpene	Cytotoxic activity	*Hippospongia sp.*	Tai-tung coast, Taiwan	106	Known
Hippolachnin A	Polyketide	Antifungal activity	*Hippospongia lachne*	The south China sea, Xisha Islands	107	New
(1*S*,5*R*)-8-((3*E*,7*E*)-4,8,12-Trimethyltrideca-3,7,11-trien-1-yl)-9-oxa-2-azabicyclo[3.3.1]nona-3,7-diene-4-carbaldehyde	Sesterterpene	Protein tyrosine phosphatase 1B (PTP1B) inhibitory activity	*Hippospongia lachne*	The south China sea	108	New

(3a*S*,4*R*,7a*S*)-4-Hydroxy-2-oxo-7-((3*E*,7*E*)-4,8,12-trimethyltrideca-3,7,11-trien-1-yl)-2,3,3a,4,5,7a-hexahydrobenzofuran-3a-carboxylic acid	Sesterterpene	Protein tyrosine phosphatase 1B (PTP1B) inhibitory activity	*Hippospongia lachne*	The south China sea	108	New
(3a*R*,4*R*,7a*S*)-4-Hydroxy-2-oxo-7-((3*E*,7*E*)-4,8,12-trimethyltrideca-3,7,11-trien-1-yl)-2,3,3a,4,5,7a-hexahydro-1H-indole-3a-carboxylic acid	Sesterterpene	Protein tyrosine phosphatase 1B (PTP1B) inhibitory activity	*Hippospongia lachne*	The south China sea	108	New
2-(2-Hydroxy-5-((3*E*,7*E*)-4,8,12-trimethyltrideca-3,7,11-trien-1-yl)phenyl)acetic acid	Sesterterpene	Protein tyrosine phosphatase 1B (PTP1B) inhibitory activity	*Hippospongia lachne*	The south China sea	108	New
(2*E*,4*E*,6*Z*)-6-(Aminomethylene)-2-((3*E*,7*E*)-4,8,12-trimethyltrideca-3,7,11-trien-1-yl)hepta-2,4-dienedial	Sesterterpene	Protein tyrosine phosphatase 1B (PTP1B) inhibitory activity	*Hippospongia lachne*	The south China sea	108	New
(-)-Hippolide J	Sesterterpene	Antifungal activity	*Hippospongia lachne*	The south China sea, Yongxing Island	109	New
(+)-Hippolide J	Sesterterpene	Antifungal activity	*Hippospongia lachne*	The south China sea, Yongxing Island	109	New
Hipposponlachnin A	Diterpene	Antiallergic activity	*Hippospongia lachne*	The south China sea, Xisha Islands	110	New
Hipposponlachnin B	Diterpene	Antiallergic activity	*Hippospongia lachne*	The south China sea, Xisha Islands	110	New
Hyattellactone A	Sesterterpene	Protein tyrosine phosphatase 1B inhibitor	*Hyattella sp.*	North Sulawesi, Indonesia	111	New
Hyattellactone B	Sesterterpene	Protein tyrosine phosphatase 1B inhibitor	*Hyattella sp.*	North Sulawesi, Indonesia	111	New
Phyllofolactone F	Sesterterpene	Protein tyrosine phosphatase 1B inhibitor	*Hyattella sp.*	North Sulawesi, Indonesia	111	Known

Phyllofolactone G	Sesterterpene	Protein tyrosine phosphatase 1B inhibitor	*Hyattella sp.*	North Sulawesi, Indonesia	111	Known
2α-Hydroxyspongia- 13(16),14-diene-3-one	Diterpene	Cytotoxic activity	*Hyattella aff. intestinalis*	Iriomote Island, Okinawa	93	New
(3b*R*,5a*R*,6*S*,9a*R*,9b*R*)-6-(Hydroxymethyl)-3b,6,9a-trimethyl-4,5,5a,6,8,9,9a,9b,10,11-decahydrophenanthro[1,2-c]furan-7(3bH)-one	Diterpene	Cytotoxic activity	*Hyattella aff. intestinalis*	Iriomote Island, Okinawa	93	Known
(3b*R*,5a*R*,7*S*,9a*R*,9b*R*)-7-Hydroxy-3b,6,6,9a-tetramethyl-3b,4,5,5a,6,7,9,9a,10,11-decahydrophenanthro[1,2-c]furan-8(9bH)-one	Diterpene	Cytotoxic activity	*Hyattella aff. intestinalis*	Iriomote Island, Okinawa	93	Known
3*β*-Hydroxyspongia-13(16),14-diene-2-one	Diterpene	Cytotoxic activity	*Hyattella aff. intestinalis*	Iriomote Island, Okinawa	93	New
2*α*,3*α*- Diacetoxy-17,19-dihydroxyspongia-13(16),14-diene	Diterpene	Cytotoxic activity	*Hyattella aff. intestinalis*	Iriomote Island, Okinawa	93	New
(3b*S*,5a*R*,6*S*,7*R*,8*S*,9a*R*,9b*R*)-3b,6-Bis(hydroxymethyl)-6,9a-dimethyl-3b,4,5,5a,6,7,8,9,9a,9b,10,11-dodecahydrophenanthro[1,2-c]furan-7,8-diol	Diterpene	Cytotoxic activity	*Hyattella aff. intestinalis*	Iriomote Island, Okinawa	93	Known
Suvanine N,N-dimethylguanidium salt	Sesterterpene	Cytotoxic activity	*Coscinoderma sp.*	Chuuk Island, Micronesia	112	Known
N,N-Dimethyl-1,3- dimethylherbipoline salt	Sesterterpene	Cytotoxic activity - antibacterial activity	*Coscinoderma sp.*	Chuuk Island, Micronesia`	112	New
Coscinolactam A	Sesterterpene	Cytotoxic activity	*Coscinoderma sp.*	Chuuk Island, Micronesia	112	Known

Coscinolactam C	Sesterterpene	Cytotoxic activity	*Coscinoderma sp.*	Chuuk Island, Micronesia	112	New
Coscinolactam D	Sesterterpene	Cytotoxic activity	*Coscinoderma sp.*	Chuuk Island, Micronesia	112	New
Coscinolactam E	Sesterterpene	Cytotoxic activity	*Coscinoderma sp.*	Chuuk Island, Micronesia	112	New
Coscinolactam F	Sesterterpene	Cytotoxic activity	*Coscinoderma sp.*	Chuuk Island, Micronesia	112	New
Coscinolactam G	Sesterterpene	Cytotoxic activity	*Coscinoderma sp.*	Chuuk Island, Micronesia	112	New
Deacyl irciniasulfonic acid C	Miscellaneous compound	NA	*Coscinoderma sp.*	Chuuk Island, Micronesia	112	New
Sodium deacyl irciniasulfonate D	Miscellaneous compound	NA	*Coscinoderma sp.*	Chuuk Island, Micronesia	112	New
**D- Family: Irciniidae**						
4-(2-((*S*)-2,2-Dimethyl-6-methylenecyclohexyl)ethyl)-5-hydroxyfuran-2(5H)-one	Sesquiterpene	PPARδ activity	*Ircinia sp.*	Korea	113	Known
(*S*)-4-(2-(2,2-Dimethyl-6-methylenecyclohexyl)ethyl)-1-isopentyl-1H-pyrrol-2(5H)-one	Meroterpene	PPARδ activity	*Ircinia sp.*	Korea	113	New
4-(2-((*S*)-2,2-Dimethyl-6-methylenecyclohexyl)ethyl)-1-(2-methylbutyl)-1H-pyrrol-2(5H)-one	Meroterpene	PPARδ activity	*Ircinia sp.*	Korea	113	New
(*S*)-4-(2-(2,2-Dimethyl-6-methylenecyclohexyl)ethyl)-1-phenethyl-1H-pyrrol-2(5H)-one	Meroterpene	PPARδ activity	*Ircinia sp.*	Korea	113	New

(*S*)-3-(2-(2,2-Dimethyl-6-methylenecyclohexyl)ethyl)-1-phenethyl-1H-pyrrol-2(5H)-one	Meroterpene	PPARδ activity (not active)	*Ircinia sp.*	Korea	113	New
3-(2-((*S*)-2,2-Dimethyl-6-methylenecyclohexyl)ethyl)-5-hydroxyfuran-2(5H)-one	Sesquiterpene	PPARδ activity (not active)	*Ircinia sp.*	Korea	113	Known
(4a*R*,6*S*,8a*S*)-4a,6-Dihydroxy-2-((1*R*,2*R*,3*R*)-2-(2-hydroxyethyl)-2-methyl-3-((*R*)-6-methylheptan-2-yl)cyclopentyl)-8a-methyl-4a,5,6,7,8,8a-hexahydronaphthalene-1,4-dione	Secosterol	Antibacterial	*Ircinia sp.*	Yeongdeok-gun in the East Sea	114	New
5-Hydroxy-1Hindole-3-carboxylic acid ethyl ester	Nitrogenous compound (Indole derivative)	NA	*Ircinia sp.*	Iriomote Island	115	New
5-Hydroxy-1Hindole-3-glyoxylate ethyl ester	Nitrogenous compound (Indole derivative)	NA	*Ircinia sp.*	Iriomote Island	115	New
Dragmacidonamine B	Nitrogenous compound (Indole derivative)	NA	*Ircinia sp.*	Iriomote Island	115	Known
Gesashidine A	Nitrogenous compound (Indole derivative)	NA	*Ircinia sp.*	Iriomote Island	115	Known
Hyrtiosulawesine	Nitrogenous compound (Indole derivative)	NA	*Ircinia sp.*	Iriomote Island	115	Known
Hyrtiomanzamine	Nitrogenous compound (Indole derivative)	NA	*Ircinia sp.*	Iriomote Island	115	Known
Hyrtimomine D	Nitrogenous compound (Indole derivative)	NA	*Ircinia sp.*	Iriomote Island	115	Known
Hyrtiosine A	Nitrogenous compound (Indole derivative)	NA	*Ircinia sp.*	Iriomote Island	115	Known
5-Hydroxy-1H-indole-3-carbaldehyde	Nitrogenous compound (Indole derivative)	NA	*Ircinia sp.*	Iriomote Island	115	Known
(4a*R*,6*S*,8a*S*)-2-((1*R*,2*R*,3*R*)-3-((2*R*,Z)-5,6-Dimethylhept-3-en-2-yl)-2-(2-hydroxyethyl)-2-methylcyclopentyl)-4a,6-dihydroxy-8a-methyl-4a,5,6,7,8,8a-hexahydronaphthalene-1,4-dione	Secosterol	Antibacterial	*Ircinia sp.*	Yeongdeok-gun in the East Sea	116	New

(7*E*, 12*E*, 20*Z*, 18*S*)-Variabilin	Sesterterpene	Protein tyrosine phosphatase (PTP) 1B inhibitor	*Ircinia sp.*	Manado, Indonesia	96	Known
(12*E*, 20*Z*, 18*S*)-8-Hydroxyvariabilin	Sesterterpene	Protein tyrosine phosphatase (PTP) 1B inhibitor	*Ircinia sp.*	Manado, Indonesia	96	Known
Felixin A	Sesterterpene	Cytotoxic activity	*Ircinia felix*	The coast of the Southern Taiwan	117	New
Felixin B	Sesterterpene	Cytotoxic activity	*Ircinia felix*	The coast of the Southern Taiwan	117	New
Felixin C	Sesterterpene	Cytotoxic activity	*Ircinia felix*	The coast of the Southern Taiwan	117	New
Felixin D	Sesterterpene	Cytotoxic activity	*Ircinia felix*	The coast of the Southern Taiwan	117	New
Felixin E	Sesterterpene	Cytotoxic activity	*Ircinia felix*	The coast of the Southern Taiwan	117	New
Felixin F	Sesterterpene	Cytotoxic activity	*Ircinia felix*	The coast of the Southern Taiwan	118	New
Felixin G	Sesterterpene	Cytotoxic activity	*Ircinia felix*	The coast of the Southern Taiwan	118	New
Ircinialactam E	Sesterterpene	Antiprotozoal activity	*Ircinia oros*	Northern Aegean Sea, Turkey	119	New

Ircinialactam F	Sesterterpene	Antiprotozoal activity	*Ircinia oros*	Northern Aegean Sea, Turkey	119	New
(7*E*,12*E*,20*Z*,18*β*)-Variabilin	Sesterterpene	NA	*Ircinia echinata*	Coto Island, Quangninh, Vietnam	120	Known
(12*E*,20*Z*,18*β*)-8-Hydroxyvariabilin	Sesterterpene	NA	*Ircinia echinata*	Coto Island, Quangninh, Vietnam	120	Known
(7*E*,11*E*,3*β*)-3,7,11-Trimethyl-14-(furan-3-yl)tetradec-7,11-dienoic acid	Sesterterpene	NA	*Ircinia echinata*	Coto Island, Quangninh, Vietnam	120	Known
Furoscalarol	Sesterterpene	NA	*Ircinia echinata*	Coto Island, Quangninh, Vietnam	120	Known
3*β*-Hydroxycholest-5-en-7-one	Sterol	NA	*Ircinia echinata*	Coto Island, Quangninh, Vietnam	120	Known
5 ,6 -Epoxycholesta-7,22(*E*)-dien-3 ,9 -diol	Epoxysterol	NA	*Ircinia echinata*	Bai Tu Long (Quang Ninh, Vietnam)	121	New

5 ,6 -Epoxycholesta-7,24(28)-dien-3 ,9 -diol	Epoxysterol	NA	*Ircinia echinata*	Bai Tu Long (Quang Ninh, Vietnam)	121	New
(24*R*)-5 ,6 -Epoxy-24-ethyl-cholesta-7-en-3 ,9 -diol	Epoxysterol	Cytotoxic activity	*Ircinia echinata*	Bai Tu Long (Quang Ninh, Vietnam)	121	New
5 ,6 -Epoxycholesta-7-en-3 ,9 -diol	Epoxysterol	Cytotoxic activity	*Ircinia echinata*	Bai Tu Long (Quang Ninh, Vietnam)	121	New
(24*S*)-5 ,6 -Epoxyergosta-7,22-dien-3 ,9 -diol	Epoxysterol	NA	*Ircinia echinata*	Bai Tu Long (Quang Ninh, Vietnam)	121	New
(24*R*)-5 ,6 -Epoxy-24-methyl-cholesta-7-en- 3 ,9 -diol	Epoxysterol	NA	*Ircinia echinata*	Bai Tu Long (Quang Ninh, Vietnam)	121	New
5 ,6 -Epoxystigmasta-7-en-3 -ol	Epoxysterol	NA	*Ircinia echinata*	Bai Tu Long (Quang Ninh, Vietnam)	121	Known
5 ,6 -Epoxystigmasta-7,22-dien-3 -ol	Epoxysterol	NA	*Ircinia echinata*	Bai Tu Long (Quang Ninh, Vietnam)	121	Known
5 ,6 -Epoxyergosta-7-en-3 -ol	Epoxysterol	NA	*Ircinia echinata*	Bai Tu Long (Quang Ninh, Vietnam)	121	Known
Sarcotragin C	Sesterterpene	Cytotoxic activity	*Sarcotragus sp.*	Chuja Island, Korea	122	New

Ircinin 1	Sesterterpene	Anticancer Activity	*Sarcotragus spinosulus*	Tabarka, Tunisia -	123	Known
		Anticancer Activity	*Psammocinia sp.*	North Sulawesi, Indonesia	124	
		Antiprotozoal activity	*Ircinia oros*	Northern Aegean Sea, Turkey	119	
Sarcotin A	Sesterterpene	Anticancer Activity.	*Sarcotragus fasciculatus*	Monastir, Tunisia	123	Known
Variabilin	Sesterterpene	Anticancer Activity.	*Sarcotragus fasciculatus*	Monastir, Tunisia	123	Known
2-(*(2E,6E,10E,14E,18E,22E*)-3,7,11,15,19,23,27-Heptamethyloctacosa-2,6,10,14,18,22,26-heptaen-1-yl)benzene-1,4-diol	Polyketide	Anticancer Activity.	*Sarcotragus spinosulus*	Monastir, Tunisia	123	Known
2-(*(2E,6E,10E,14E,18E,22E,26E*)-3,7,11,15,19,23,27,31-Octamethyldotriaconta-2,6,10,14,18,22,26,30-octaen-1-yl)benzene-1,4-diol	Polyketide	Anticancer Activity.	*Sarcotragus spinosulus*	Tabarka, Tunisia	123	Known
2′-[38-Hydroxy]nonaprenyl-1′,4′-hydroquinone	Polyketide	Anticancer Activity.	*Sarcotragus foetidus*	Bizerte, Tunisia	123	Known
12-Deacetoxy-23-hydroxyscalaradial	Sesterterpene	Cytotoxic activity	*Psammocinia sp.*	The South Sea of Korea	125	New
12-Dehydroxy- 23-hydroxyhyrtiolide	Sesterterpene	Cytotoxic activity	*Psammocinia sp.*	The South Sea of Korea	125	New
12-*O*-Acetyl-16-deacetoxy-23-acetoxyscalarafuran	Sesterterpene	Cytotoxic activity	*Psammocinia sp.*	The South Sea of Korea	125	New
12-Deacetoxy-23-hydroxyheteronemin	Sesterterpene	Cytotoxic activity	*Psammocinia sp.*	The South Sea of Korea	125	Known
12-Deacetoxy-23-acetoxy-19-O-acetylscalarin	Sesterterpene	Cytotoxic activity	*Psammocinia sp.*	The South Sea of Korea	125	Known
12-Deacetoxy-23-O-acetoxyheteronemin	Sesterterpene	Cytotoxic activity	*Psammocinia sp.*	The South Sea of Korea	125	Known
12-Deacetoxyscalaradial	Sesterterpene	Cytotoxic activity	*Psammocinia sp.*	The South Sea of Korea	125	Known

(−)-Ircinianin	Sesterterpene	Modulation of glycine-gated chloride channel receptors (GlyRs) (modest potentiator)	*Psammocinia sp.*	Southern Australia	126	Known
(−)-Ircinianin sulfate	Sesterterpene	Modulation of glycine-gated chloride channel receptors (GlyRs) (modest antagonist)	*Psammocinia sp.*	Southern Australia	126	Known
(−)-Ircinianin lactam A	Sesterterpene	Modulation of glycine-gated chloride channel receptors (GlyRs) (no significant effect)	*Psammocinia sp.*	Southern Australia	126	New
(−)-Ircinianin lactam A sulfate	Sesterterpene	Modulation of glycine-gated chloride channel receptors (GlyRs) (modest potentiator)	*Psammocinia sp.*	Southern Australia	126	New
(−)-Oxoircinianin	Sesterterpene	Modulation of glycine-gated chloride channel receptors (GlyRs) (no significant effect)	*Psammocinia sp.*	Southern Australia	126	New

(−)-Oxoircinianin lactam A	Sesterterpene	Modulation of glycine-gated chloride channel receptors (GlyRs) (selective potentiator of α1 GlyRs)	*Psammocinia sp.*	Southern Australia	[126]	New
(−)-Icinianin lactone A	Sesterterpene	Modulation of glycine-gated chloride channel receptors (GlyRs) (no significant effect)	*Psammocinia sp.*	Southern Australia	[126]	New
Sulawesin A	Sesterterpene	Anticancer Activity.	*Psammocinia sp.*	North Sulawesi, Indonesia	[124]	New
Sulawesin B	Sesterterpene	Anticancer Activity.	*Psammocinia sp.*	North Sulawesi, Indonesia	[124]	New
Sulawesin C	Sesterterpene	Anticancer Activity.	*Psammocinia sp.*	North Sulawesi, Indonesia	[124]	New
Ircinin 2	Sesterterpene	Anticancer Activity	*Psammocinia sp.*	North Sulawesi, Indonesia	[124]	Known
		Antiprotozoal activity	*Ircinia oros*	Northern Aegean Sea, Turkey	[119]	

E- Artifacts from order Dictyoceratida:						
α16- Methoxyfuroscalarol	Sesterterpene	Cytotoxic activity	*Scalarispongia sp.*	Dokdo (island), Republic of Korea	[37]	Artifact
β16- Methoxyfuroscalarol	Sesterterpene	Cytotoxic activity	*Scalarispongia sp.*	Dokdo (island), Republic of Korea	[37]	Artifact
20-Demethoxy-20-ethoxydactyloquinone E	Sesquiterpene	Cytotoxic activity	*Spongia pertusa Esper*	Yongxing Island	[99]	Artifact
20-Demethoxy-20-ethoxydactyloquinone B	Sesquiterpene	Cytotoxic activity	*Spongia pertusa Esper*	Yongxing Island	[99]	Artifact
20-Demethoxy-20-ethoxycyclospongiaquinone-1	Sesquiterpene	Cytotoxic activity	*Spongia pertusa Esper*	Yongxing Island	[99]	Artifact
3,5-Dibromo-2-(30,50-dibromo-20-methoxyphenoxy)-1-methoxybenzene	Halogenated compound	Inhibit Protein tyrosine phosphatase 1B activity	*Lamellodysidea herbacea*	Manado, Indonesia	[89]	Prepared
3,5-Dibromo-2-(30,50-dibromo-20-methoxyphenoxy)phenyl ethanoate	Halogenated compound	Inhibit Protein tyrosine phosphatase 1B activity - cytotoxic	*Lamellodysidea herbacea*	Manado, Indonesia	[89]	Prepared
3,5-Dibromo-2-(30,50-dibromo-20-methoxyphenoxy)phenyl butanoate	Halogenated compound	Inhibit Protein tyrosine phosphatase 1B activity - cytotoxic	*Lamellodysidea herbacea*	Manado, Indonesia	[89]	Prepared
3,5-Dibromo-2-(30,50-dibromo-20-methoxyphenoxy)phenyl hexanoate	Halogenated compound	Inhibit Protein tyrosine phosphatase 1B activity - cytotoxic	*Lamellodysidea herbacea*	Manado, Indonesia	[89]	Prepared
3,5-Dibromo-2-(30,50-dibromo-20-methoxyphenoxy)phenyl benzoate	Halogenated compound	Inhibit Protein tyrosine phosphatase 1B activity - cytotoxic	*Lamellodysidea herbacea*	Manado, Indonesia	[89]	Prepared

(−)-Ircinianin acetate	Sesterterpene	Modulation of glycine-gated chloride channel receptors (GlyRs) (no significant effect)	*Psammocinia sp.*	Southern Australia	126	Acetylation of (−)-ircinianin
(2*R*,3*R*,4*S*,5*R*)-3-(2-((*S*)-2,2-Dimethyl-6-methylenecyclohexyl)ethyl)-2,5-dimethoxytetrahydrofuran-3,4-diol	Terpene	PPARδ agonistic activity	*Ircinia sp.*	Korea	113	artifact
(2*R*,4*S*,5*R*)-3-(2-((*S*)-2,2-Dimethyl-6-methylenecyclohexyl)ethyl)-2,5-dimethoxytetrahydrofuran-3,4-diol	Terpene	PPARδ agonistic activity	*Ircinia sp.*	Korea	113	artifact
Salarin A	Macrolide	Cytotoxic activity	*Fascaplysinopsis sp.*	Madagascar	127	New
Salarin C	Macrolide	Cytotoxic activity	*Fascaplysinopsis sp.*	Madagascar	127	New
Tulearin A	Macrolide	Cytotoxic activity	*Fascaplysinopsis sp.*	Madagascar	127	New

References:

1. D. Youssef, L. Shaala and H. Asfour,Bioactive compounds from the Red Sea marine sponge Hyrtios species, *Mar. Drugs*, 2013, 11, 1061-1070.22
2. R. Momose, N. Tanaka, J. Fromont and J. i. Kobayashi,Hyrtimomines A–C, New Heteroaromatic Alkaloids from a Sponge Hyrtios sp, *Organic Lett.*, 2013, 15, 2010-2013.
3. N. Tanaka, R. Momose, Y. Takahashi, T. Kubota, A. Takahashi-Nakaguchi, T. Gonoi, J. Fromont and J. i. Kobayashi,Hyrtimomines D and E, bisindole alkaloids from a marine sponge Hyrtios sp, *Tetrahedron Lett.*, 2013, 54, 4038-4040.
4. N. Tanaka, R. Momose, A. Takahashi-Nakaguchi, T. Gonoi, J. Fromont and J. i. Kobayashi,Hyrtimomines, indole alkaloids from Okinawan marine sponges Hyrtios spp, *Tetrahedron*, 2014, 70, 832-837.
5. F. Yang, J. H. Gan, X. Y .Liu and H. W. Lin,Scalarane sesterterpenes from the Paracel Islands marine sponge Hyrtios sp, *Nat. Prod. Commun.*, 2014, 9, 763-764.
6. C. Festa, C. Cassiano, M. V. D'Auria, C. Debitus, M. C. Monti and S. De Marino,Scalarane sesterterpenes from Thorectidae sponges as inhibitors of TDP-43 nuclear factor, *Org. Biomol. Chem.*, 2014, 12, 8646-8655.
7. W. Kaweetripob, C. Mahidol, P. Tuntiwachwuttikul, S. Ruchirawat and H. Prawat,Cytotoxic Sesterterpenes from Thai Marine Sponge Hyrtios erectus, *Mar. Drugs*, 2018, 16, 1-15.
8. W. M. Alarif, S. S. Al-Lihaibi, M. A. Ghandourah, M. I. Orif, S. A. Basaif and S.-E. N. Ayyad,Cytotoxic scalarane-type sesterterpenes from the Saudi Red Sea sponge Hyrtios erectus, *J. Asian Nat. Prod. Res.*, 2016, 18, 611-617.
9. A. Alahdal, H. Asfour, S. Ahmed, A. Noor, A. Al-Abd, M. Elfaky and S. Elhady,Anti-helicobacter, antitubercular and cytotoxic activities of scalaranes from the red sea sponge hyrtios erectus, *Molecules*, 2018, 23, 1-11.
10. S-.Y. Wu, P.-J. Sung, Y.-L. Chang, S.-L. Pan and C.-M. Teng,Heteronemin, a spongean sesterterpene, induces cell apoptosis and autophagy in human renal carcinoma cells, *Biomed. Res.Int.*, 2015, 2015 ,1-13.
11. S. Elhady, A. Al-Abd, A. El-Halawany, A. Alahdal, H. Hassanean and S. Ahmed,Antiproliferative scalarane-based metabolites from the Red Sea sponge Hyrtios erectus, *Mar. Drugs*, 2016, 14, 1-14.

12. S. Elhady, A. El-Halawany, A. Alahdal, H. Hassanean and S. Ahmed,A new bioactive metabolite isolated from the Red Sea marine sponge Hyrtios erectus, *Molecules*, 2016, 21, 1-9.
13. T. Kubota, K. Nakamura, K. Sakai, J. Fromont, T. Gonoi and J. i. Kobayashi,Hyrtinadines C and D, New Azepinoindole-Type Alkaloids from a Marine Sponge Hyrtios sp, *Chem. Pharm. Bull.*, 2016, 64, 975-978.
14. J. Wang, F.-R. Mu, W.-H. Jiao, J. Huang, L.-L. Hong, F. Yang, Y. Xu, S.-P. Wang, F. Sun and H.-W. Lin,Meroterpenoids with protein tyrosine phosphatase 1B inhibitory activity from a Hyrtios sp. marine sponge, *J. Nat. Prod.*, 2017, 80, 2509-2514.
15. T. S. Hilliard, G. Miklossy, C. Chock, P. Yue, P. Williams and J. Turkson,15α-Methoxypuupehenol induces antitumor effects in vitro and in vivo against human glioblastoma and breast cancer models, *Mol. CancerTher.*, 2017, 16, 601-613.
16. N. H. Shady, M. A. Fouad, S. Ahmed, S. M. Pimentel-Elardo, J. R. Nodwell, M. S. Kamel and U. R. Abdelmohsen,A new antitrypanosomal alkaloid from the Red Sea marine sponge Hyrtios sp, *J. Antibiot.*, 2018, 71, 1036–1039.
17. J. Li, L. Du, M. Kelly, Y.-D. Zhou and D. G. Nagle,Structures and potential antitumor activity of sesterterpenes from the marine sponge Hyrtios communis, *J. Nat. Prod.*, 2013, 76, 1492-1497.
18. W.-F. He, D.-Q. Xue, L-.G. Yao, J.-Y. Li, J. Li and Y.-W. Guo,Hainanerectamines A–C, alkaloids from the Hainan sponge Hyrtios erecta, *Mar. Drugs*, 2014, 12, 3982-3993.
19. E. Ju, A. Latif, C.-S. Kong, Y. Seo, Y.-J. Lee, S. R. Dalal, M. B. Cassera and D. G. Kingston,Antimalarial activity of the isolates from the marine sponge Hyrtios erectus against the chloroquine-resistant Dd2 strain of Plasmodium falciparum, *Z Naturforsch C*, 2018, 73, 397-400.
20. M. S. Majik, D. Shirodkar, C. Rodrigues, L. D'Souza and S. Tilvi,Evaluation of single and joint effect of metabolites isolated from marine sponges, Fasciospongia cavernosa and Axinella donnani on antimicrobial properties, *Bioorg. Med. Chem. Lett.*, 2014, 24, 2863-2866.
21. E. Sakai, H. Kato, H. Rotinsulu, F. Losung, R. E. Mangindaan, N. J. de Voogd, H. Yokosawa and S. Tsukamoto,Variabines A and B: New β-carboline alkaloids from the marine sponge Luffariella variabilis, *J. Nat. Med.*, 2014, 68, 215-219.
22. U. W. Hawas, L. T. Abou El-Kassem, M. S. Abdelfattah, M. I. Elmallah, M. A. G. Eid, M. M. Monier and N. Marimuthu,Cytotoxic activity of alkyl benzoate and fatty acids from the red sea sponge Hyrtios erectus, *Nat. Prod. Res.*, 2018, 32, 1369-1374.
23. H. Wahab, N. Pham, T. Muhammad, J. Hooper and R. Quinn,Merosesquiterpene congeners from the Australian Sponge Hyrtios digitatus as potential drug leads for atherosclerosis disease, *Mar. Drugs*, 2017, 15, 1-10.
24. R. Teta, E. Irollo, G. Della Sala, G. Pirozzi, A. Mangoni and V. Costantino,Smenamides A and B, chlorinated peptide/polyketide hybrids containing a dolapyrrolidinone unit from the Caribbean sponge Smenospongia aurea. Evaluation of their role as leads in antitumor drug research, *Mar. Drugs*, 2013, 11, 4451-4463.
25. G .Esposito, G. Della Sala, R. Teta, A. Caso, M. L. Bourguet-Kondracki, J. R. Pawlik, A. Mangoni and V. Costantino,Chlorinated Thiazole-Containing Polyketide-Peptides from the Caribbean Sponge Smenospongia conulosa: Structure Elucidation on Microgram Scale ,*Eur. J.Org. Chem.*, 2016, 2016, 2871-2875.
26. I. H. Hwang, J. Oh, A. Kochanowska-Karamyan, R. J. Doerksen, M. Na and M. T. Hamann,A novel natural phenyl alkene with cytotoxic activity, *Tetrahedron Lett.*, 2013, 54, 3872-3876.
27. G. Esposito, R. Teta, R. Miceli, L. Ceccarelli, G. Della Sala, R. Camerlingo, E. Irollo, A. Mangoni, G. Pirozzi and V. Costantino,Isolation and assessment of the in vitro anti-tumor activity of smenothiazole A and B, chlorinated thiazole-containing peptide/polyketides from the Caribbean sponge, Smenospongia aurea, *Mar. Drugs*, 2015, 13, 444-459.
28. I. H. Hwang, J. Oh, W. Zhou, S. Park, J.-H. Kim, A. G. Chittiboyina, D. Ferreira, G. Y. Song, S. Oh and M. Na,Cytotoxic activity of rearranged drimane meroterpenoids against colon cancer cells via down-regulation of β-catenin expression, *J. Nat. Prod.*, 2015, 78, 453-461.
29. G. Esposito, R. Teta, G. Della Sala, J. Pawlik, A. Mangoni and V. Costantino,Isolation of smenopyrone, a Bis-γ-pyrone polypropionate from the Caribbean sponge Smenospongia aurea, *Mar. Drugs*, 2018, 16, 1-9.
30. R. Teta, G. Della Sala, G. Esposito, C. W. Via, C. Mazzoccoli, C. Piccoli, M. J. Bertin, V. Costantino and A. Mangoni,A joint molecular networking study of a Smenospongia sponge and a cyanobacterial bloom revealed new antiproliferative chlorinated polyketides, *Org. Chem.Front.*, 2019, 6, 1762-1774.
31. T. H. Le, D. T. T. Hang, N. X. Nhiem, P. H. Yen, H. L. T. Anh, T. H. Quang, B. H. Tai, N. Van Dau and P. Van Kiem,Naphtoquinones and sesquiterpene cyclopentenones from the sponge Smenospongia cerebriformis with their cytotoxic activity, *Chem. Pharm. Bull.*, 2017, 65, 589-592.
32. P. Van Kiem, D. T. Hang, N. X. Nhiem, B. H. Tai, H. L. T. Anh, P. Van Cuong, T. H. Quang, C. Van Minh, N. Van Dau and Y.-A. Kim,Sesquiterpene derivatives from marine sponge Smenospongia cerebriformis and their anti-inflammatory activity, *Bioorg. Med. Chem. Lett.*, 2017, 27, 1525-1529.
33. L. T. Huyen, D .T. Hang, N. X. Nhiem, B. H. Tai, H. L. T. Anh, T. H. Quang, P. H. Yen, C. Van Minh, N. Van Dau and P. Van Kiem,Sesquiterpene quinones and diterpenes from Smenospongia cerebriformis and their cytotoxic activity, *Nat. Prod. Commun.*, 2017, 12, 477-478.
34. Z.-B. Zhao, J.-Z. Sun, S.-C. Mao and Y.-W. Guo,Fasciospyrinadine, a novel sesquiterpene pyridine alkaloid from a Guangxi sponge Fasciospongia sp, *J. Asian Nat. Prod. Res.*, 2013, 15, 198-202.

35. Y. J. Lee, D. G. Lee, H. S. Rho, V. B. Krasokhin, H. J. Shin, J. S. Lee and H. S. Lee,Cytotoxic 5-Hydroxyindole Alkaloids from the Marine Sponge Scalarispongia sp, *J. Heterocycl.Chem.*, 2013, 50, 1400-1404.
36. Y.-J. Lee, J.-W. Lee, D.-G. Lee, H.-S. Lee, J. Kang and J. Yun,Cytotoxic sesterterpenoids isolated from the marine sponge Scalarispongia sp, *Int. J. Mol. Sci.*, 2014, 15, 20045-20053.
37. Y.-J. Lee, S. H. Kim, H. Choi, H.-S. Lee, J. S. Lee, H. J. Shin and J. Lee,Cytotoxic Furan-and Pyrrole-Containing Scalarane Sesterterpenoids Isolated from the Sponge Scalarispongia sp, *Molecules*, 2019, 24, 1-9.
38. J. G. Starkus, P. Poerzgen, K. Layugan, K. G. Kawabata, J.-I. Goto, S. Suzuki, G. Myers, M. Kelly, R. Penner and A. Fleig,Scalaradial is a potent inhibitor of transient receptor potential melastatin 2 (TRPM2) ion channels, *J. Nat. Prod.*, 2017, 80, 2741-2750.
39. S. Khushi, L. Nahar, A. Salim and R. Capon,Cacolides: Sesterterpene Butenolides from a Southern Australian Marine Sponge, Cacospongia sp, *Mar. Drugs*, 2018, 16, 1-17.
40. X. Zhang, P.-L. Li, G.-F. Qin, S. Li, N. de Voogd, X.-L. Tang and G.-Q. Li,Isolation and Absolute Configurations of Diversiform C17, C21 and C25 Terpenoids from the Marine Sponge Cacospongia sp ,*Mar. Drugs*, 2019, 17, 1-14.
41. T. Taufa, A. J. Singh, C. R. Harland, V. Patel, B. Jones, T. i. Halafihi, J. H. Miller, R. A. Keyzers and P. T. Northcote,Zampanolides B–E from the Marine Sponge Cacospongia mycofijiensis: Potent Cytotoxic Macrolides with Microtubule-Stabilizing Activity, *J. Nat. Prod.*, 2018, 81, 2539-2544.
42. A. D. Przeslak, M. Inman, W. Lewis and C. J. Moody,Origin of the Thiopyrone CTP-431 “Unexpectedly” Isolated from the Marine Sponge Cacospongia mycofijiensis, *J. Org. Chem.*, 2018, 83, 10595-10601.
43. M. H. Hassan, M. E. Rateb, M. Hetta, T. A. Abdelaziz, M. A. Sleim, M. Jaspars and R. Mohammed,Scalarane sesterterpenes from the Egyptian Red Sea sponge Phyllospongia lamellosa, *Tetrahedron*, 2015, 71, 577-583.
44. S. Sun, C. Canning, K. Wang, W. Zhu, F. Yang, Y. Zhang and K. Zhou,Antibacterial Activity of 2-(3',5'-Dibromo-2'-methoxyphenoxy)-3,5- dibromophenol Isolated from Phyllospongia papyracea, *Nat. Prod. Commun.*, 2017, 12, 567-569.
45. H .Zhang, P. Crews, K. Tenney and F. A Valeriote,Cytotoxic phyllactone analogs from the marine sponge Phyllospongia papyrecea, *Med. Chem.*, 2017, 13, 295-300.
46. L. Harinantenaina, P. J. Brodie, J. Maharavo, G. Bakary, K. TenDyke, Y. Shen and D. G .Kingston,Antiproliferative homoscalarane sesterterpenes from two Madagascan sponges, *Bioorg. Med. Chem.*, 2013, 21, 2912-2917.
47. F. Cao, Z.-H. Wu, C.-L. Shao, S. Pang, X.-Y. Liang, N. J. de Voogd and C.-Y. Wang,Cytotoxic scalarane sesterterpenoids from the South China Sea sponge Carteriospongia foliascens, *Org. Biomol. Chem.*, 2015, 13, 4016-4024.
48. K.-H. Lai, Y.-C. Liu, J.-H. Su, M. El-Shazly, C.-F. Wu, Y.-C. Du, Y.-M. Hsu, J.-C. Yang, M.-K. Weng and C.-H. Chou,Antileukemic scalarane sesterterpenoids and meroditerpenoid from Carteriospongia (Phyllospongia) sp., induce apoptosis via dual inhibitory effects on topoisomerase II and Hsp90, *Sci. Rep.*, 2016, 6, 1-14.
49. K. Hagiwara, J. E. Garcia Hernandez, M. K. Harper, A. Carroll, C. A. Motti, J. Awaya, H.-Y. Nguyen and A. D. Wright,Puupehenol, a potent antioxidant antimicrobial meroterpenoid from a Hawaiian deep-water Dactylospongia sp. sponge, *J. Nat. Prod.*, 2015, 78, 325-329.
50. J. Li, F. Yang, Z. Wang, W. Wu, L. Liu, S.-P. Wang, B.-X. Zhao, W.-H. Jiao, S.-H. Xu and H.-W. Lin,Unusual anti-inflammatory meroterpenoids from the marine sponge Dactylospongia sp, *Org. Biomol. Chem.*, 2018, 16, 6773-6782.
51. S. S. Ebada, N. de Voogd, R. Kalscheuer, W. E. Müller and P. Proksch,Cytotoxic drimane meroterpenoids from the Indonesian marine sponge Dactylospongia elegans, *Phytochem. Lett.*, 2017, 22, 154-158.
52. G. Daletos, N. J. de Voogd, W. E. Müller, V. Wray, W. Lin, D. Feger, M. Kubbutat ,A. H. Aly and P. Proksch,Cytotoxic and protein kinase inhibiting nakijiquinones and nakijiquinols from the sponge Dactylospongia metachromia, *J. Nat. Prod.*, 2014, 77, 218-226.
53. L. Du, Y.-D. Zhou and D. G. Nagle,Inducers of hypoxic response: Marine sesquiterpene quinones activate HIF-1, *J. Nat. Prod.*, 2013, 76, 1175-1181.
54. W. Balansa, U. Mettal, Z. G. Wuisan, A. Plubrukarn, F. G. Ijong, Y. Liu and T. F. Schäberle,A New Sesquiterpenoid Aminoquinone from an Indonesian Marine Sponge, *Mar. Drugs*, 2019, 17, 1-7.
55. R. Zhong, C.-L. Shao, N. J. de Voogd and C.-Y. Wang,Sesquiterpene derivatives and steroids from the sponge Dactylospongia elegans collected from the south China sea, *Chem. Nat. Compd.*, 2014, 50, 1-3.
56. H.-B. Yu, B.-B. Gu, S.-P. Wang, C.-W. Cheng, F. Yang and H.-W. Lin,New diterpenoids from the marine sponge Dactylospongia elegans, *Tetrahedron*, 2017, 73, 6657-6661.
57. J. Li, Z. Wang, F. Yang, W.-H. Jiao, H.-W. Lin and S.-H. Xu,Two new steroids with cytotoxicity from the marine sponge Dactylospongia elegans collected from the South China Sea, *Nat. Prod. Res.*, 2019, 33, 1340-1344.
58. J. Li, W. Wu, F. Yang, L. Liu, S. P. Wang, W. H. Jiao, S. H. Xu and H. W. Lin,Popolohuanones G–I, Dimeric Sesquiterpene Quinones with IL-6 Inhibitory Activity from the Marine Sponge Dactylospongia elegans, *Chem*. *Biodivers*., 2018, 15, 1-7.

59. H.-B. Yu, Z.-F. Yin, B.-B. Gu, J.-P. Zhang, S.-P. Wang, F. Yang and H.-W. Lin,Cytotoxic meroterpenoids from the marine sponge Dactylospongia elegans, *Nat. Prod. Res.*, 2019, 24, 1-7.
60. Q. Wang, X. Tang, X. Luo, N. J. de Voogd, P. Li and G. Li,(+)- and (−)-Spiroreticulatine, A Pair of Unusual Spiro Bisheterocyclic Quinoline-imidazole Alkaloids from the South China Sea Sponge Fascaplysinopsis reticulata, *Org. Lett.*, 2015, 17, 3458-3461.
61. G.-F. Qin, X.-L. Tang, N. J. de Voogd, P.-L. Li and G.-Q. Li,Cytotoxic components from the Xisha sponge Fascaplysinopsis reticulata, *Nat. Prod. Res.*, 2018, DOI: 10.1080/14786419.2018.1502765, 1-7.
62. P.-E. Campos, E. Pichon, C. Moriou, P. Clerc, R. Trépos, M. Frederich, N. De Voogd, C. Hellio, A. Gauvin-Bialecki and A. Al-Mourabit,New Antimalarial and Antimicrobial Tryptamine Derivatives from the Marine Sponge Fascaplysinopsis reticulata, *Mar. Drugs*, 2019, 17, 1-10.
63. Q. Wang, X.-L. Tang, X.-C. Luo, N. J. de Voog, P.-L. Li and G.-Q. Li,Aplysinopsin-type and Bromotyrosine-derived Alkaloids from the South China Sea Sponge Fascaplysinopsis reticulata, *Sci. Rep.*, 2019, 9, 1-10.
64. T.-R. Su, Z.-J. Liao, M.-C. Lu, Y.-J. Wu and J.-H. Su,Cytotoxic Monocarbocyclic Sesterterpenoids from a Marine Sponge Luffariella sp, *Bull. Chem. Soc. Jpn.*, 2015,88,176-182.
65. A. Faricha, P. Ahmadi, N. J. Devoogd and J. Tanaka,Two isospongian diterpenes from the sponge luffariella sp, *Nat. Prod. Commun.*, 2017, 12, 1011-1012.
66. P. Ahmadi, M. Higashi, N. J. d. Voogd and J. Tanaka,Two Furanosesterterpenoids from the Sponge Luffariella variabilis, *Mar. Drugs*, 2017, 15, 1-8.
67. S. De Caralt, D. Bry, N. Bontemps, X. Turon, M.-J. Uriz and B. Banaigs,Sources of Secondary Metabolite Variation in Dysidea avara (Porifera: Demospongiae): The Importance of Having Good Neighbors, *Mar. Drugs*, 2013, 11,489-503.
68. A. Hamed, W. Wätjen, R. Schmitz, Y. Chovolou, R. Edrada-Ebel, D. Youssef, M. Kamel and C. Chaidir,A New Bioactive Sesquiterpenoid Quinone from the Mediterranean Sea Marine Sponge Dysidea avara, *Nat. Prod. Commun.*, 2013, 8, 289-292.
69. W.-H. Jiao, T.-T. Xu, H.-B. Yu, G.-D. Chen, X.-J. Huang, F. Yang, Y.-S. Li, B.-N. Han, X.-Y. Liu and H.-W. Lin,Dysideanones A–C, Unusual Sesquiterpene Quinones from the South China Sea Sponge Dysidea avara ,*J. Nat. Prod.*, 2014, 77, 346-350.
70. W.-H. Jiao, T.-T. Xu, B.-B. Gu, G.-H. Shi, Y. Zhu, F. Yang, B.-N. Han, S.-P. Wang, Y.-S. Li and W. Zhang,Bioactive sesquiterpene quinols and quinones from the marine sponge Dysidea avara, *RSC Adv.*, 2015,58,7730-.87738
71. D. B. Abdjul, H. Yamazaki, O. Takahashi, R. Kirikoshi, K. Ukai and M. Namikoshi,Sesquiterpene Hydroquinones with Protein Tyrosine Phosphatase 1B Inhibitory Activities from a Dysidea sp. Marine Sponge Collected in Okinawa, *J. Nat. Prod.*, 2016, 79, 1842-1847.
72. A. Trianto, N. J. de Voodg and J. Tanaka,Two new compounds from an Indonesian sponge Dysidea sp, *J. Asian Nat. Prod. Res.*, 2014, 16, 163-168.
73. W.-H. Jiao, J. Li, Q. Liu, T.-T. Xu, G.-H. Shi, H.-B. Yu, F. Yang, B.-N. Han, M. Li and H.-W. Lin,Dysidinoid A, an Unusual Meroterpenoid with Anti-MRSA Activity from the South China Sea Sponge Dysidea sp, *Molecules*, 2014, 19, 18025-18032.
74. S. Sun, C. B. Canning, K. Bhargava, X. Sun, W. Zhu, N. Zhou, Y. Zhang and K. Zhou,Polybrominated diphenyl ethers with potent and broad spectrum antimicrobial activity from the marine sponge Dysidea, *Bioorg. Med. Chem. Lett.*, 2015, 25, 2181-2183.
75. C.-K. Kim, J.-K. Woo, S.-H. Kim, E. Cho, Y.-J. Lee, H.-S .Lee, C. J. Sim, D.-C. Oh, K.-B. Oh and J. Shin,Meroterpenoids from a tropical Dysidea sp. sponge, *J. Nat. Prod.*, 2015, 78, 2814-2821.
76. X. Zhang, H.-Y. Xu, A.-M. Huang, L. Wang, Q. Wang, P.-Y. Cao and P.-M. Yang,Antibacterial Meroterpenoids from the South China Sea Sponge <i>Dysidea</i> sp, *Chem. Pharm. Bull.*, 2016, 64, 1036-1042.
77. Y. Lu and M. Zhao,Two highly acetylated sterols from the marine sponge Dysidea sp, *Z Naturforsch B*, 2017, 72, 49-52.
78. P. V. Kiem, C. V. Minh, N. X. Nhiem, N. T. Cuc, N. V. Quang, H. L. Tuan Anh, B. H. Tai, P. H. Yen, N. T. Hoai, K. Y. Ho, N. Kim, S. Park and S. H. Kim,Muurolane-type sesquiterpenes from marine sponge Dysidea cinerea, *Magn. Reson. Chem.*, 2014,52,51-56.
79. P. Van Kiem, N. X. Nhiem, N. Van Quang, C. Van Minh, N. H. Nam, N. T. Cuc, H. T. Le Anh, B. H. Tai, P. H. Yen, N. X. Cuong, N. P. Thao, N. T. Hoai, N. Y. Kim, S. J. Park and K. S. Hyun,New butenolide and pentenolide from Dysidea cinerea, *Nat. Prod. Commun.*, 2013, 8, 1751-1752.
80. W.-H. Jiao, T.-T. Xu, H.-B. Yu, F.-R. Mu, J. Li, Y.-S. Li, F. Yang, B.-N. Han and H.-W. Lin,Dysidaminones A–M, cytotoxic and NF-κB inhibitory sesquiterpene aminoquinones from the South China Sea sponge Dysidea fragilis, *Rsc Adv.*, 2014, 4, 9236-9246.
81. N. T. Cue, T. Anh Hle, D. T. Hang, N. X. Nhiem, N. H. Dang, N. H. Nama, P. H. Yen, C. Thung do, V. K. Thuc, C. V. Minh and P. V. Kiema,Sesquiterpenes from the Vietnamese Marine Sponge Dysidea fragilis, *Nat. Prod. Commun.*, 2015, 10, 1341-1342.
82. P. V. Kiem, N. X. Nhiem, B. H. Tai, T. Anh Hle, D. T. Hang, N. T. Cuc, T. Huyen le, N. H. Nam, P. H. Yen, C. Thung do and C. V. Minh,Bis-sesquiterpene from the Marine Sponge Dysidea fragilis, *Nat. Prod. Commun.*, 2016, 11, 439-441.

83. H. Li, Q. Zhang, X. Jin, X. Zou, Y. Wang, D. Hao, F. Fu, W. Jiao, C. Zhang and H. Lin,Dysifragilone A inhibits LPS‑induced RAW264. 7 macrophage activation by blocking the p38 MAPK signaling pathway, *Mol. Med. Rep.*, 2018, 17, 674-682.
84. W.-H. Jiao, B.-H. Cheng, G.-H. Shi, G.-D. Chen, B.-B. Gu, Y.-J. Zhou, L.-L. Hong, F. Yang, Z.-Q. Liu, S.-Q. Qiu, Z.-G. Liu, P.-C. Yang and H.-W. Lin,Dysivillosins A–D, Unusual Anti-allergic Meroterpenoids from the Marine Sponge Dysidea villosa, *Sci. Rep.*, 2017, 7, 1-10.
85. H. Liu, K. Lohith, M. Rosario, T. H. Pulliam, R. D. O'Connor, L. J. Bell and C. A. Bewley,Polybrominated diphenyl ethers: structure determination and trends in antibacterial activity, *J. Nat. Prod.*, 2016, 79, 1872-1876.
86. M. Arai, D. Shin, K. Kamiya, R. Ishida, A. Setiawan, N. Kotoku and M. Kobayashi,Marine spongean polybrominated diphenyl ethers, selective growth inhibitors against the cancer cells adapted to glucose starvation, inhibits mitochondrial complex II, *J. Nat. Med.*, 2017, 71, 44-49.
87. M. Shingaki, T. Wauke, P. Ahmadi and J. Tanaka,Four cytotoxic spongian diterpenes from the sponge Dysidea cf. arenaria, *Chem. Pharm. Bull.*, 2016, 64, 272-275.
88. Y.-H. Gui, W.-H. Jiao, M. Zhou, Y. Zhang, D.-Q. Zeng, H.-R. Zhu, K.-C. Liu, F. Sun, H.-F. Chen and H.-W. Lin,Septosones A–C, in Vivo Anti-inflammatory Meroterpenoids with Rearranged Carbon Skeletons from the Marine Sponge Dysidea septosa, *Org. Lett.*, 2019, 21, 767-770.
89. H. Yamazaki, D. A. Sumilat, S.-i. Kanno, K. Ukai, H. Rotinsulu, D. S. Wewengkang, M. Ishikawa, R. E. Mangindaan and M. Namikoshi,A polybromodiphenyl ether from an Indonesian marine sponge Lamellodysideaherbacea and its chemical derivatives inhibit protein tyrosine phosphatase 1B, an important target for diabetes treatment, *J. Nat. Med.*, 2013, 67, 730-735.
90. M. Torii, H. Kato, Y. Hitora, E. D. Angkouw, R. E. P. Mangindaan, N. J. de Voogd and S. Tsukamoto,Lamellodysidines A and B, Sesquiterpenes Isolated from the Marine Sponge Lamellodysidea herbacea, *J. Nat. Prod.*, 2017, 80, 2536-2541.
91. D.-Y. Sun, G.-Y. Han, N.-N. Yang, L.-F. Lan, X.-W. Li and Y.-W. Guo,Racemic trinorsesquiterpenoids from the Beihai sponge Spongia officinalis: structure and biomimetic total synthesis, *Org. Chem.Front.*, 2018, 5, 1022-1027.
92. G.-Y. Han, D.-Y. Sun, L.-F. Liang, L.-G. Yao, K.-X. Chen and Y.-W. Guo,Spongian diterpenes from Chinese marine sponge Spongia officinalis, *Fitoterapia*, 2018, 127, 159-165.
93. P. Ahmadi, T. Haruyama, N. Kobayashi, N. J. de Voogd and J. Tanaka,Spongian Diterpenes from the Sponge <i>Hyattella</i> aff. <i>intestinalis</i>, *Chem. Pharm. Bull.*, 2017, 65, 874-877.
94. S. M. Parrish, W. Y. Yoshida, T. P. Kondratyuk, E.-J. Park, J. M. Pezzuto, M. Kelly and P. G. Williams,Spongiapyridine and Related Spongians Isolated from an Indonesian Spongia sp, *J. Nat. Prod.*, 2014, 77, 1644-16.49
95. I. Yang, S.-J. Nam and H. Kang,Two New Scalaranes from a Korean Marine Sponge Spongia sp, *Nat. Prod. Sci.*, 2015, 21, 289-292.
96. D. B. Abdjul, H. Yamazaki, S.-i. Kanno, D. S. Wewengkang, H. Rotinsulu, D. A. Sumilat, K. Ukai, M. M. Kapojos and M. Namikoshi,Furanoterpenes, new types of protein tyrosine phosphatase 1B inhibitors, from two Indonesian marine sponges, Ircinia and Spongia spp, *Bioorg. Med. Chem. Lett.*, 2017, 27, 1159-1161.
97. T. Ito, H. M. Nguyen, N. N. Win, H. Q. Vo, H .T. Nguyen and H. Morita,Three new sesquiterpene aminoquinones from a Vietnamese Spongia sp. and their biological activities, *J. Nat. Med.*, 2018, 72, 298-303.
98. I. Yang, J. Lee, J. Lee, D. Hahn, J. Chin, H. D. Won, J. Ko, H. Choi, A. Hong, S.-J. Nam and H. Kang,Scalalactams A–D, Scalarane Sesterterpenes with a γ-Lactam Moiety from a Korean Spongia Sp. Marine Sponge, *Molecules*, 2018, 23,1-10.
99. J. Li, B.-B. Gu, F. Sun, J.-R. Xu, W.-H. Jiao, H.-B. Yu, B.-N. Han, F. Yang, X.-C. Zhang and H.-W .Lin,Sesquiterpene quinones/hydroquinones from the marine sponge Spongia pertusa Esper, *J. Nat. Prod.*, 2017, 80, 1436-1445.
100. A. H. El-Desoky, H. Kato, E. D. Angkouw, R. E. P. Mangindaan, N. J. de Voogd and S. Tsukamoto,Ceylonamides A–F ,Nitrogenous Spongian Diterpenes That Inhibit RANKL-Induced Osteoclastogenesis, from the Marine Sponge Spongia ceylonensis, *J. Nat. Prod.*, 2016, 79, 1922-1928.
101. A. H. El-Desoky, H. Kato, I. Kagiyama, Y. Hitora, F. Losung, R. E. P. Mangindaan, N. J. de Voogd and S. Tsukamoto,Ceylonins A–F, Spongian Diterpene Derivatives That Inhibit RANKL-Induced Formation of Multinuclear Osteoclasts, from the Marine Sponge Spongia ceylonensis, *J. Nat. Prod.*, 2017, 80, 90-95.
102. A. H. El-Desoky, H. Kato and S. Tsukamoto,Ceylonins G–I: spongian diterpenes from the marine sponge Spongia ceylonensis, *J. Nat. Med.*, 2017, 71, 765-769.
103. R. Kumar, R. Subramani and W. Aalbersberg,Three bioactive sesquiterpene quinones from the Fijian marine sponge of the genus Hippospongia, *Nat. Prod. Res.*, 2013, 27, 1488-1491.
104. Y.-M. Fuh, M.-C. Lu, C.-H. Lee and J.-H. Su,Cytotoxic scalarane sesterterpenoids from a marine sponge Hippospongia sp, *Nat. Prod. Commun.*,2013,8,571-572.
105. C. C. Su, H. J. Su, K. J. Liang, S. J. Tsaif and J. H. Su,A Norsesterterpene Peroxide from a Marine Sponge Hippospongia sp, *Nat. Prod. Commun.*, 2016, 11, 445-446.
106. Y.-C. Chen, M.-C. Lu, M. El-Shazly, K.-H. Lai, T.-Y. Wu ,Y.-M. Hsu, Y.-L. Lee and Y.-C. Liu,Breaking down Leukemia Walls: Heteronemin, a Sesterterpene Derivative, Induces Apoptosis in Leukemia Molt4 Cells through Oxidative Stress, Mitochondrial Dysfunction and Induction of Talin Expression, *Mar. Drugs*, 2018,16,1-22.

107. S.-J. Piao, Y.-L. Song, W.-H. Jiao, F. Yang, X.-F. Liu, W.-S. Chen, B.-N. Han and H.-W. Lin,Hippolachnin A, a new antifungal polyketide from the South China sea sponge Hippospongia lachne, *Org. Lett.*, 2013, 15, 3526-3529.
108. S.-J. Piao, W-.H. Jiao, F. Yang, Y.-H. Yi, Y.-T. Di, B.-N. Han and H.-W. Lin,New Hippolide Derivatives with Protein Tyrosine Phosphatase 1B Inhibitory Activity from the Marine Sponge Hippospongia lachne, *Mar. Drugs*, 2014, 12, 4096-4109.
109. W. H. Jiao, L. L. Hong, J. B. Sun, S .J. Piao, G. D. Chen, H. Deng, S. P. Wang, F. Yang and H. W. Lin,(±)-Hippolide J–A Pair of Unusual Antifungal Enantiomeric Sesterterpenoids from the Marine Sponge Hippospongia lachne, *Eur. J.Org. Chem.*, 2017, 2017, 3421-3426.
110. L-.L. Hong, H.-B. Yu, J. Wang, W.-H. Jiao, B.-H. Cheng, F. Yang, Y.-J. Zhou, B.-B. Gu, S.-J. Song and H.-W. Lin,Unusual Anti-allergic Diterpenoids from the Marine Sponge Hippospongia lachne, *Sci. Rep.*, 2017, 7, 1-7.
111. D. B. Abdjul, H. Yamazaki ,O. Takahashi, R. Kirikoshi, R. E. P. Mangindaan and M. Namikoshi,Two new protein tyrosine phosphatase 1B inhibitors, hyattellactones A and B, from the Indonesian marine sponge Hyattella sp, *Bioorg. Med. Chem. Lett.*, 2015, 25, 904-907.
112. C.-K. Kim, I.-H. Song, H. Y. Park, Y.-J. Lee, H.-S. Lee, C. J. Sim, D.-C. Oh, K.-B. Oh and J. Shin,Suvanine sesterterpenes and deacyl irciniasulfonic acids from a tropical Coscinoderma sp. sponge, *J. Nat. Prod.*, 2014, 77, 1396-1403.
113. D. Hahn, J. Chin, H. Kim, I. Yang, D. H. Won, M. Ekins, H. Choi, S.-J. Nam and H. Kang,Sesquiterpenoids with PPARδ agonistic effect from a Korean marine sponge Ircinia sp, *Tetrahedron Lett.*, 2014, 55, 4716-4719.
114. I. Yang, H. Choi, D. H. Won, S.-J .Nam and H. Kang,An antibacterial 9, 11-secosterol from a marine sponge Ircinia sp, *Bull. Korean Chem. Soc.*, 2014, 35, 3360-3362.
115. D. B. Abdjul, H. Yamazaki, K. Ukai and M. Namikoshi,Two new indole derivatives from a marine sponge Ircinia sp. collected at Iriomote Island, *J. Nat. Med.*, 2015, 69, 416-420.
116. I. Yang, H. Choi, S.-J. Nam and H. Kang,A new 9,11-secosterol with a 1,4-quinone from a Korean marine sponge Ircinia sp, *Arch. Pharm. Res.*, 2015, 38, 1970-1974.
117. Y.-Y. Lai, M.-C. Lu, L.-H. Wang, J.-J. Chen, L.-S. Fang, Y.-C. Wu and P.-J. Sung,New Scalarane Sesterterpenoids from the Formosan Sponge Ircinia felix, *Mar. Drugs*, 2015, 13, 4296-4309.
118. Y.-Y. Lai, L.-C. Chen, C.-F. Wu, M.-C. Lu, Z.-H. Wen, T.-Y .Wu, L.-S. Fang, L.-H. Wang, Y.-C. Wu and P.-J. Sung,New cytotoxic 24-homoscalarane sesterterpenoids from the sponge Ircinia felix, *Int. J. Mol. Sci.*, 2015, 16, 21950-21958.
119. G. Chianese, J. Silber, P. Luciano, C. Merten, D .Erpenbeck, B. Topaloglu, M. Kaiser and D. Tasdemir,Antiprotozoal Linear Furanosesterterpenoids from the Marine Sponge Ircinia oros, *J. Nat. Prod.*, 2017, 80, 2566-2571.
120. D. T. Dung, N. X. Nhiem, B. H. Tai, P. H. Yen, H. L. T. Anh, D. C. Thung, C. Van Minh and P. Van Kiem,Furanosesterterpenes from the marine sponge Ircinia echinata (Keller, 1889), *Vietnam J. Chem.*, 2016, 54, 477-482.
121. T. T. Trinh, N. B. Truong, A. Longeon, M. T. Doan, A. Deville, M. V. Chau, C. V. Pham and M.-L .Bourguet-Kondracki,New 9α-Hydroxy-5α,6α-epoxysterols from the Vietnamese Marine Sponge Ircinia echinata, *Mar. Drugs*, 2018, 16,1-10.
122. J.-K. Woo, J.-e. Jeon, B. Kim, C. J. Sim, D.-C. Oh, K.-B. Oh and J. Shin,A New Sesterterpene from the Korean Sarcotragus sp. Sponge, *Nat. Prod. Sci.*, 2015, 21, 237-239.
123. Y. BenRedjem Romdhane, M. Elbour, M. Carbone, M. L. Ciavatta, M. Gavagnin, V. Mathieu, #xe9, ronique, F. Lefranc, L. Ktari, K. Ben Mustapha, A. Boudabous, R. Kiss and E. Mollo,In Vitro Growth Inhibitory Activities of Natural Products from Irciniid Sponges against Cancer Cells: A Comparative Study, *Biomed Res. Int.*, 2016, 2016, 1-6.
124. A. H. Afifi, I. Kagiyama, A. H. El-Desoky, H. Kato, R. E. Mangindaan, N. J. de Voogd, N. M. Ammar, M. S. Hifnawy and S. Tsukamoto,Sulawesins A–C, furanosesterterpene tetronic acids that inhibit USP7, from a Psammocinia sp. marine sponge, *J. Nat. Prod.*, 2017, 80, 2045-2050.
125. D. Hahn, D. H. Won, B. Mun, H. Kim, C. Han, W. Wang, T. Chun, S. Park ,D. Yoon, H. Choi, S.-J. Nam, M. Ekins, J. Chin and H. Kang,Cytotoxic scalarane sesterterpenes from a Korean marine sponge Psammocinia sp, *Bioorg. Med. Chem. Lett.*, 2013, 23, 2336-2339.
126. W. Balansa, R. Islam, F. Fontaine, A. M. Piggott, H. Zhang, X. Xiao, T. I. Webb, D. F. Gilbert, J. W. Lynch and R. J. Capon,Sesterterpene glycinyl-lactams: a new class of glycine receptor modulator from Australian marine sponges of the genus Psammocinia, *Org. Biomol. Chem.*, 2013, 11, 4695-.4701
127. L. Zur, A. Bishara, M. Aknin, D. Neumann, N. Ben-Califa and Y. Kashman,Derivatives of Salarin A, Salarin C and Tulearin A—Fascaplysinopsis sp. Metabolites, *Mar. Drugs*, 2013, 11, 4487-4509.
